# Wide-Band Interference Mitigation in GNSS Receivers Using Sub-Band Automatic Gain Control †

**DOI:** 10.3390/s22020679

**Published:** 2022-01-16

**Authors:** Johannes Rossouw van der Merwe, Fabio Garzia, Alexander Rügamer, Santiago Urquijo, David Contreras Franco, Wolfgang Felber

**Affiliations:** Satellite-Based Positioning Systems Department, Fraunhofer IIS, Nordostpark 84, 90411 Nuremberg, Germany; alexander.ruegamer@iis.fraunhofer.de (A.R.); santiago.urquijo@iis.fraunhofer.de (S.U.); contredd@iis.fraunhofer.de (D.C.F.); wolfgang.felber@iis.fraunhofer.de (W.F.)

**Keywords:** global navigation satellite system (GNSS), automatic gain control (AGC), interference mitigation (IM), high-rate DFT-based data manipulator (HDDM), sub-band processing

## Abstract

The performance of global navigation satellite system (GNSS) receivers is significantly affected by interference signals. For this reason, several research groups have proposed methods to mitigate the effect of different kinds of jammers. One effective method for wide-band interference mitigation (IM) is the high-rate DFT-based data manipulator (HDDM) pulse blanker (PB). It provides good performance to pulsed and frequency sparse interference. However, it and many other methods have poor performance against wide-band noise signals, which are not frequency-sparse. This article proposes to include automatic gain control (AGC) in the HDDM structure to attenuate the signal instead of removing it: the HDDM-AGC. It overcomes the wide-band noise limitation for IM at the cost of limiting mitigation capability to other signals. Previous studies with this approach were limited to only measuring the carrier-to-noise density ratio ($C/N_{0}$) performance of tracking, but this article extends the analysis to include the impact of the HDDM-AGC algorithm on the position, velocity, and time (PVT) solution. It allows an end-to-end evaluation and impact assessment of mitigation to a GNSS receiver. This study compares two commercial receivers: one high-end and one low-cost, with and without HDDM IM against laboratory-generated interference signals. The results show that the HDDM-AGC provides a PVT availability and precision comparable to high-end commercial receivers with integrated mitigation for most interference types. For pulse interferences, its performance is superior. Further, it is shown that degradation is minimized against wide-band noise interferences. Regarding low-cost receivers, the PVT availability can be increased up to 40% by applying an external HDDM-AGC.

## 1. Introduction

Interference mitigation is gaining importance in the research and development of GNSS receivers since incidents related to jammers are more and more frequent and are also widely documented in the literature [1,2,3,4,5]. Simpler mitigation methods like a PB or a notch filter are ineffective against complex interference signals. These include pulsed noise, frequency-modulated continuous-wave (FMCW) (also referred to as “chirp” or “swept-frequency” signals), frequency hopping, matched spectrum, or a combination of any of these [5]. Additionally, new GNSS signals present a higher complexity, typically associated with a larger bandwidth. It makes the development of IM methods even more challenging.

The HDDM algorithm provides higher adaptability and wide-band IM [6]. The HDDM is similar to the frequency-domain adaptive filtering (FDAF) method [7,8,9]. However, the fast Fourier transform (FFT) is calculated for every newly-received digital sample of the signal instead of a block of samples. As a result, this oversampling increases the time selectivity and limit ringing and distortion effects.

Initially, the HDDM was proposed as a software implementation [6], using a simple PB per channel to remove interference signals. Later, the first hardware (HW) implementation [10] for a GNSS receiver was implemented and tested against wide-band noise interferences [11]. However, since then, the HDDM has been proven useful for general signal conditioning methods beyond IM, including equalization, spectrum compression, and signal corrections [12].

A limitation of the HDDM-PB in IM is that it—like many other IM methods—completely removes the signal in the presence of wide-band noise interferences. Thereby completely disrupting the GNSS receiver. Using an AGC instead of a PB overcomes this limitation [11], and was proposed with an initial HW prototype at the International Conference on Localization and GNSS (ICL-GNSS) 2021. This article extends the conference article by providing an in-depth performance analysis of the HDDM-AGC. It includes an evaluation of the PVT solution as impacted by the interference signal and mitigation methods, determining the position errors and availability. Further, the HDDM-AGC is evaluated using high-end and low-cost commercial-off-the-shelf (COTS) commercial receivers to facilitate a fair performance comparison. The HDDM-AGC mitigates the interference signal in the digital domain on a stand-alone platform before the signal is sent to the COTS receivers.

This article aims to present an end-to-end analysis of the mitigation performance of the HDDM-AGC against an array of interferences. Therefore, establishing a benchmark to the current state-of-the-art IM capabilities and identifying which interference signals remain challenges for future research. Results confirm that wide-band noise interferences no longer disrupt the entire signal during mitigation, proving the versatility of the HDDM-AGC. However, it also alters the performance against other interference signals. A limitation of this study was the digital-to-analog converter (DAC) of the HDDM-AGC mitigation platform, which, due to limited bit-lengths, resulted in a $C/N_{0}$ loss of 6 dB, making a direct comparison to the state-of-the-art challenging. Nevertheless, the HDDM-AGC could outperform the state-of-the-art for select interferences, like pulsed interferences, despite this handicap.

The remainder of the article is structured as follows: Section 2 provides a background to the HDDM and related mitigation methods, and Section 3 details the HW design and implementation. Section 4 describes the test setup and the evaluation methodology. The results are shown in Section 5, and discussed in Section 6. Finally, the conclusions are drawn in Section 7.

## 2. Background

**Figure 1:**
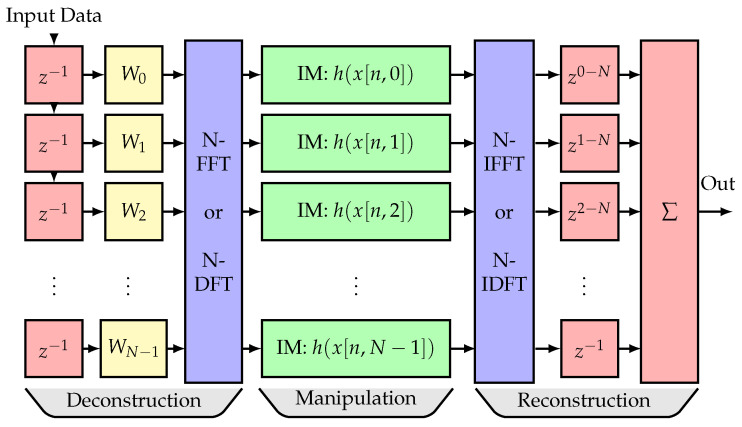
HDDM block diagram. ©IEEE. Reprinted, with permission, from [11].

The HDDM algorithm has shown sound performance IM [6,13]. Figure 1 shows the general block diagram of the HDDM for IM. It uses a discrete Fourier transform (DFT)—or the more efficient FFT—to de-interleave a signal into multiple sub-bands. The sub-bands allow for time-frequency manipulation of the signals [12]. The signal manipulation could follow several goals, like restructuring the spectrum [14], equalizing the spectrum [12], or mitigating an interference signal [13]. The classic approach uses a simple PB to remove interference signals exceeding a power threshold [6,13]. The PB provides temporal isolation and removal, and the DFT interleaving of the HDDM provides spectral isolation of an interference. Therefore, this approach is ideal for removing FMCW, which are sparse in the time-frequency domain. The HDDM improves temporal resolution compared to other Fourier-based techniques, e.g., FDAF [15,16]. It is the reason for the superior performance against pulsed signals [13].

A challenge in IM is to develop versatile algorithms that can mitigate a range of different interference types. Therefore, a common limitation of mitigation algorithms is the limitation to a single type of interference. For example, a PB is good at removing wide-band pulsed signals [17] but is ineffective against any continuous-wave (CW) signals. An opposing method is adaptive notch filtering (ANF), which excels at frequency-sparse FMCW [18,19,20,21], but it is ineffective against multi-spectral signals [22] or pulsed signals. Multi-spectral methods, which use a DFT, discrete wavelet transform (DWT), or Karhunen-Loève transform (KLT) to de-interleave the signal into different components, provide more versatility to mitigation [23], but often are not feasible for efficient HW implementation. Finally, a wide-band GNSS receiver requires algorithms with low latency to allow real-time processing at high rates, often requiring some form of parallelization to meet these requirements. Therefore, practical and versatile methods are desired, but few algorithms meet these requirements.

Previous studies [13] used simple PB to remove large spectral components for IM. A PB is simple and requires low resources, making it ideal for HW implementations. However, with wide-band noise signals, the inherent interference-suppression properties of the code-division multiple access (CDMA) based signals may yield better results than removing large sections of the spectrum. Therefore, PB would cause more harm than benefit to the IM method. One approach uses non-linear functions [24] instead of completely blanking a signal. However, such non-linear functions are complex and impractical to implement in HW, often disregarding fixed-point limitations of high-speed digital systems. Using a simple AGC could provide a similar effect but significantly lower processing requirements.

## 3. Design and Implementation

The HDDM-AGC algorithm uses the HDDM [6] to deconstruct an input signal into multiple frequency bands. It is achieved through a shift register, a window function, an *N*-Point FFT. Each frequency band has a separate IM AGC module. At the end of the process, the HDDM reconstructs the frequency bands back together in a single output data stream. This is realized using an inverse fast Fourier transform (IFFT), a triangular delay register, and a combiner.

**Figure 2:**
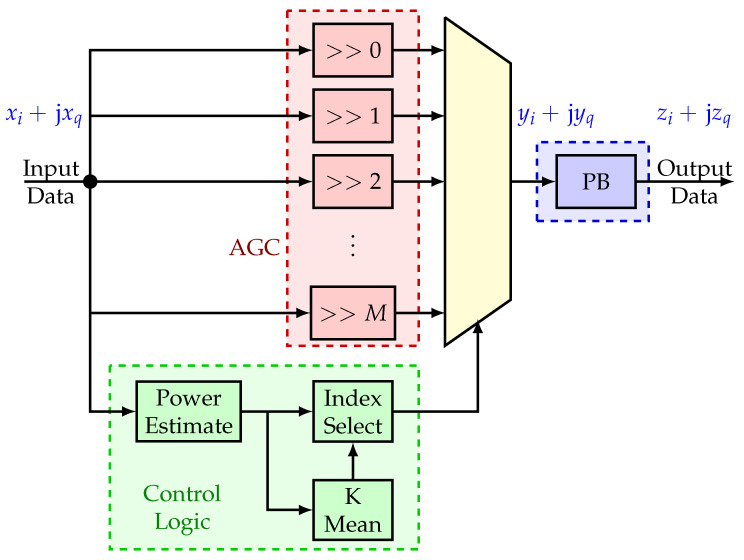
AGC module block diagram. ©IEEE. Reprinted, with permission, from [11].

The AGC module performs the IM and spectrum regulation. Figure 2 shows the block diagram of the AGC module. It consists of four sections. First, a resource-efficient bit-shift AGC is implemented (red box, top left). The benefit is that no multiplication is used–saving significantly on digital signal processor (DSP) slices for firmware implementations. The number of shifts *M* depends on the implementation. Second, a control logic circuit (green box, bottom left) determines the average signal amplitude in the frequency band. It approximates the mean signal amplitude over *K*-Samples:

(1) $$c\left[n,l\right]=\frac{1}{K}\sum_{m=0}^{K-1}\left|x_{i}\left[n-m,l\right]\right|$$

where $x_{i}\left[n,l\right]$ is the in-phase component of the signal at the *n*–th sample for the *l*–th channel of the HDDM, $\left|\cdot \right|$ is the absolute operator, and c[n,l] is the control value for the bit-shifting AGC. An empirical evaluation determined that using both the in-phase $x_{i}\left[n,l\right]$ and the quadrature-phase $x_{q}\left[n,l\right]$ of the signal for this implementation did not yield a significant improvement and was omitted to limit resource use. A larger value of *K* implies a slower but more stable AGC response. The control signal is then mapped to the number of bit-shifts:

(2) $$\hat{p}\left[n,l\right]=\left\lfloor \log_{2}\left(\lambda_{a}\times c\left[n,l\right]\right)\right\rceil$$

where $\lambda_{a}$ is a scaling factor, $\left\lfloor \cdot \right\rceil$ rounds the value to the nearest integer, and $\hat{p}\left[n,l\right]$ is the number of bits for the signal to shift. Lastly, the shifting bits are limited to the AGC range:

(3) $$p\left[n,l\right]=\left\{\begin{array}{cc} 0 & \;\mathrm{if}\;\hat{p}\left[n,l\right]<0\\ M & \;\mathrm{if}\;\hat{p}\left[n,l\right]>M\\ \hat{p}\left[n,l\right] & \;\mathrm{otherwise} \end{array}\right.$$

where p[n,l] is the final bit-shifts. An alternative approach would be to use the energy (i.e., $x_{i}^{2}\left[n-m,l\right]+x_{q}^{2}\left[n-m,l\right]$) for the control signal (theoretically, this would be the better approach). However, it requires an additional multiplication which increases field-programmable gate array (FPGA) complexity unnecessary with an insignificant improvement to the AGC adaption capability. This optimization is a key trade-off in the FPGA design.

Third, the control logic drives a multiplexer (yellow multiplexer, center) which selects the appropriate AGC channel. The in-phase $y_{i}\left[n\right]$ and quadrature-phase $y_{q}\left[n\right]$ output of the AGC stage is then defined as

(4) $$y_{i}\left[n\right]=x_{i}\left[n\right]\times 2^{-p[n,l]}$$

(5) $$y_{q}\left[n\right]=x_{q}\left[n\right]\times 2^{-p[n,l]}$$

Finally, a PB is included (blue box, right) to blank any large values to which the AGC could not respond in time. The blanking is defined as

(6) $$z_{i}\left[n\right]=\left\{\begin{array}{cc} y_{i}\left[n\right] & \;\mathrm{if}\;y_{i}\left[n\right]<\lambda_{p}\;\mathrm{or}\;y_{q}\left[n\right]<\lambda_{p}\\ 0 & \;\mathrm{otherwise} \end{array}\right.$$

(7) $$z_{q}\left[n\right]=\left\{\begin{array}{cc} y_{q}\left[n\right] & \;\mathrm{if}\;y_{i}\left[n\right]<\lambda_{p}\;\mathrm{or}\;y_{q}\left[n\right]<\lambda_{p}\\ 0 & \;\mathrm{otherwise} \end{array}\right.$$

where $\lambda_{p}$ is the PB threshold, and $z_{i}\left[n\right]$ and $z_{q}\left[n\right]$ are the in-phase and quadrature-phase outputs, respectively. The full mitigation can be described as:

(8) $$z_{i}\left[n\right]+\;jz_{q}\left[n\right]=h\left(x_{i}\left[n\right]+\;jx_{q}\left[n\right],l\right)$$

where $h\left(\cdot ,l\right)$ is a non-linear function with memory to map the inputs to the outputs of the IM for the *l*-th channel of the HDDM.

The HW implementation is done in register-transfer level (RTL) code (i.e., very-high-speed integrated circuit hardware description language (VHDL)), allowing the deployment on different FPGA technologies or even as an application-specific integrated circuit (ASIC) intellectual property (IP). The central processing blocks are the DFT and inverse discrete Fourier transform (IDFT)—both based on a standard Cooley-Tukey radix-2 FFT core. The HDDM FFT bit width is driven by the input signal dynamic range and constrained by resource utilization and timing requirements. In addition to the FFT, the windowing and the reconstruction modules are also optimized for digital HW implementation. The windowing is based on a combination of bit shifts, to avoid instantiating multiplier blocks. The reconstruction module adder is implemented using a transposed structure, which puts the adder in the pipeline [13].

The AGC implementation is described in detail in [11]. This article avoids multipliers and dividers through the left and right shifting. It constrains the size of the window of samples to a power of two, which does not introduce any relevant limitation.

The HDDM hardware module has been implemented on two FPGA-based GNSS dual-band receivers, having different costs, form factors, and target applications. A summary of the resource utilization on different Xilinx devices is given in [11].

## 4. Test Setup

### 4.1. Physical Setup

**Figure 3:**
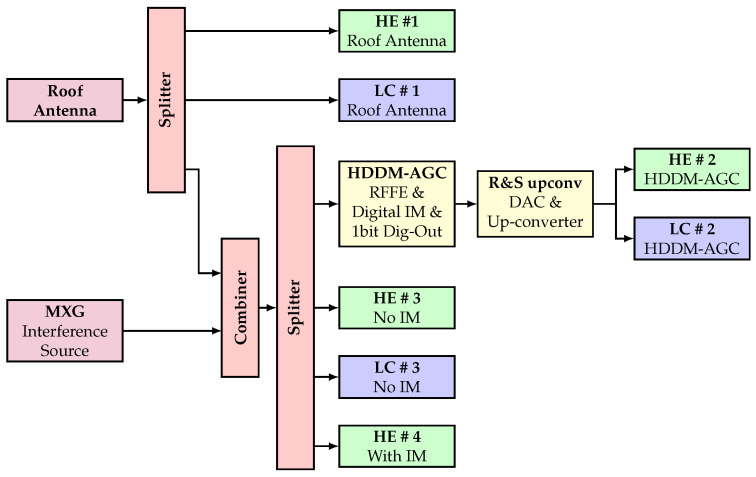
Experimental setup.

Figure 3 shows the experimental setup. The setup has two signal sources. First, a stationary geodetic-grade antenna placed on the roof provides GNSS signals for the test setup. The signal is also adequately amplified to accommodate for losses expected by the setup and the connection to the roof. Second, interference signals are generated in the laboratory with an Agilent MXG-series vector signal generator. The output power is varied over time to analyze different interference-to-noise ratios (INRs).

COTS GNSS receivers are used for analysis, as this focuses the test setup on the IM capabilities and does not limit it to the differences in GNSS processing. Two types of COTS receivers are evaluated to provide a broader range of analyses. First, a mass-market low-cost (LC) receiver (blue blocks in Figure 3) with limited built-in IM is used. This receiver is configured to use GPS L1 CA and Galileo E1BC. Second, a high-end (HE) geodetic grade receiver (green blocks in Figure 3) with built-in IM is used. Receivers HE #2, #3, and #4 are configured to only use GPS L1 CA and Galileo E1BC, but receiver HE #1 is configured to be multi-band and multi-system to provide maximum precision. A radio-frequency (RF) network connects the two signal sources to various receivers. These fall into four categories:

1. Roof Antenna: Receivers HE #1 and LC #1 are connected to the roof antenna without interference. These provide the interference-free ground truth signals.
2. HDDM-AGC: Receivers HE #2 and LC #2 get GNSS and interference signals. The signals are received with an radio-frequency front-end (RFFE), where the HDDM-AGC mitigation is implemented in firmware as described in Section 3. Unfortunately, this platform does not have an onboard DAC. Therefore, the most significant bit of the I and Q components of the signal after mitigation is up-converted back to the L1 band using a Rohde&Schwarz signal generator. The mitigated signal is then passed to the two receivers. Note that as this process only uses a 1-bit DAC, significant quantization loss is introduced [25].
3. No IM: Receivers HE #3 and LC #3 both have GNSS signals and interference signals, but no mitigation is enabled. The HE #3 receiver is explicitly configured to bypass all IM.
4. With IM: In the HE #4 receiver wide-band IM capabilities are enabled. It allows a direct comparison of the HDDM-AGC to the state-of-the-art IM.

### 4.2. Test Procedure

Several interference waveforms are tested: a single waveform per test. The output power of the Agilent signal generator starts at −70 dBm, increases in 1 dB steps to 0 dBm, then decreases in 1 dB steps back to −70 dBm. The dwell time on each power step is 30 s to provide the receiver with sufficient time to stabilize. Consequently, a single test takes 1 h and 10 min. These tests are long, resulting in a significant risk that several satellites are not visible for the entire duration of the test.

The interference signals are composed of nine generated interferences, which are replayed using an Agilent MXG-series vector signal generator. The generated interferences include single-tone, wide-band chirp signals, frequency hopping signals, noise signals, and pulsed signals. These interferences are chosen as they represent different scenarios, stressing the resilience capabilities of all receivers. The list of interference with a summary of their properties:

- Interference #1—CW: single-tone interference at 1.57542 GHz,
- Interference #2—fast chirp: wide-band linear chirp with 10 MHz bandwidth and a chirp repetition rate of 10 µs,
- Interference #3—slow chirp: wide-band linear chirp with 10 MHz bandwidth and a chirp repetition rate of 100 µs,
- Interference #4—slow hopper: frequency hopper with a dwell time of 100 µs and a frequency range of 35 MHz,
- Interference #5—fast hopper: frequency hopper with a dwell time of 1 µs and a frequency range of 35 MHz,
- Interference #6—noise: filtered noise with 4 MHz bandwidth,
- Interference #7—noise: filtered noise with 35 MHz bandwidth,
- Interference #8—slow pulse: filtered pulsed noise with 35 MHz bandwidth, 50% duty cycle, and 1 ms pulse width,
- Interference #9—fast pulse: filtered pulsed noise with 35 MHz bandwidth, 50% duty cycle, and 100 µs pulse width.

Note that only a subset of the interference signals is presented and discussed in Section 5. However, all interference types are available in the Appendix A for the interested reader, and the results for all satellites are published as Supplementary Material to the article.

The $C/N_{0}$ for all satellites and the PVT solution reported by each receiver for each interference is recorded and analyzed. First, the $C/N_{0}$ is evaluated to determine the stand-alone benefit of the IM. This is the classical interference analysis approach. In Section 5, only selected GPS L1 C/A satellites with selected interference signals are presented and discussed. However, the results of both GPS L1 C/A and Galileo E1B/C are available in the Appendix B. Second, the position errors and the PVT availability is evaluated to determine the end-to-end impact of IM. This brings the analysis closer to system-level verification and testing.

Lastly, for additional information and insight, an interference detector is included. It uses machine learning (ML) and features extracted from a low-cost NeSDR digital video broadcasting–terrestrial (DVB-T) dongle. The detector is still under development and will be showcased in a future publication. Nevertheless, it still provides additional insight into mitigation performance.

### 4.3. Estimation of Interference to Noise Ratio

The output power of the Agilent MXG-series signal generator is selected to be between −70 dBm and 0 dBm. However, this is not a meaningful measure of the interference power. The interference-to-signal ratio (ISR) is often used to characterize interference tests, but the problem is that each satellite is received at a different $C/N_{0}$. Therefore, the ISR is not a practical measure when comparing the PVT solutions with each other or different satellites in the same scenario. The INR compares the interference power to the thermal noise of each receiver. It is independent of the satellite signals and is consequently the same for all satellites. The ISR can be converted to the INR for a given satellite if the $C/N_{0}$ and the bandwidth of the receiver *B* are known:

(9) $$\mathrm{INR}=\mathrm{ISR}\cdot \frac{C/N_{0}}{B}$$

(10) $${\mathrm{INR}}_{\mathrm{dB}}={\mathrm{ISR}}_{\mathrm{dB}}+{\left(C/N_{0}\right)}_{\mathrm{dB}}-10\log_{10}\left(B\right)\;\left[\mathrm{dB}\right]$$

Although the INR is a better comparison on a single receiver, it is a function of the receiver bandwidth. It makes a comparison invalid, as different receivers have different analog bandwidths. Therefore, the interference-to-noise density ratio ($I/N_{0}$), which normalizes the INR to the receiver bandwidth, is the only fair metric regardless of the receiver analog bandwidth and the received signal. The $I/N_{0}$ is defined as

(11) $$I/N_{0}=\mathrm{ISR}\cdot C/N_{0}=\mathrm{INR}\cdot B$$

(12) $${\left(I/N_{0}\right)}_{\mathrm{dB}}={\mathrm{ISR}}_{\mathrm{dB}}+{\left(C/N_{0}\right)}_{\mathrm{dB}}={\mathrm{INR}}_{\mathrm{dB}}+10\log_{10}\left(B\right)\;\left[\mathrm{dBHz}\right]$$

The $I/N_{0}$ is less intuitive, but it is analogous to the $C/N_{0}$, familiarizing it to the GNSS community. The $I/N_{0}$ is displayed in the results for consistency, but it is fairly simple to convert to the INR or ISR, as demonstrated in Equation (12).

**Figure 4:**
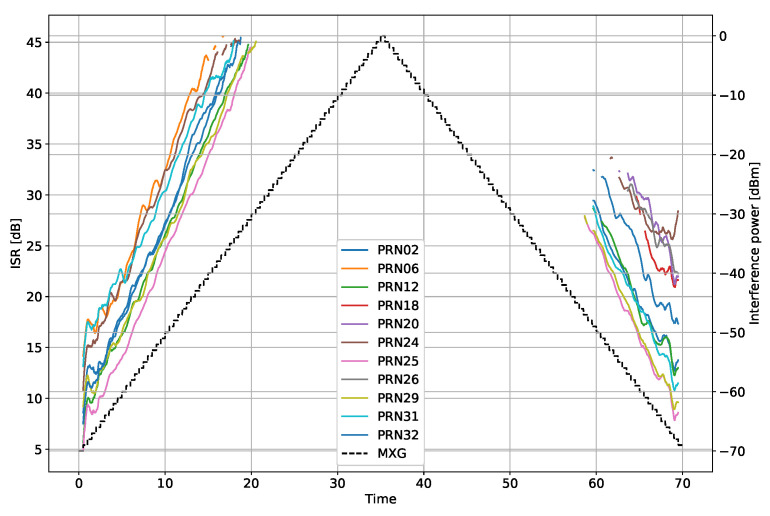
Estimated ISR from the 4 MHz bandwidth filtered noise interference scenario.

The $I/N_{0}$ is estimated based on the signal generator power and the spectral separation coefficient (SSC). The effect of the SSC on the $C/N_{0}$ is the ${\left(C/N_{0}\right)}_{\mathrm{eff}}$. This is defined as [26,27]:

(13) $${\left(C/N_{0}\right)}_{\mathrm{eff}}=\frac{1}{\frac{1}{C/N_{0}}+\frac{\mathrm{ISR}}{Q_{j}\cdot R_{c}}}$$

where $C/N_{0}$ is the interference-free, $Q_{j}$ the jamming resistive quality factor, which measures how efficient the interference is, and $R_{c}$ the chipping rate of the GNSS signal. The jamming resistive quality factor $Q_{j}$ is a function of the GNSS signal PSD and the interference signal PSD. First, the $Q_{j}$ is determined for a 4 MHz filtered noise interference signal, and a GPS L1 C/A BPSK(1) is determined to be $Q_{j}=4.11$. Second, the ISR for GPS L1 C/A satellites for the 4 MHz filtered noise interference is determined:

(14) $${\mathrm{ISR}}_{\mathrm{est}}\left[n,m\right]=\left(\frac{1}{{\left(C/N_{0}\right)}_{\mathrm{NoIM}}\left[n,m\right]}-\frac{1}{{\left(C/N_{0}\right)}_{\mathrm{Roof}}\left[n,m\right]}\right)\cdot Q_{j}\cdot R_{c}$$

where ${\mathrm{ISR}}_{\mathrm{est}}\left[n,m\right]$ is the estimate for the *n*–th observation for the *m*–th satellite, ${\left(C/N_{0}\right)}_{\mathrm{Roof}}$ is the measurement from the HE roof antenna, and ${\left(C/N_{0}\right)}_{\mathrm{NoIM}}$ is the measurements from the HE with the IM switched off. Figure 4 shows the estimated ISR compared to the transmit power. Note the biases between the various satellites.

Third, the $I/N_{0}$ is estimated from the ISR using the roof $C/N_{0}$ values:

(15) $${\left(I/N_{0}\right)}_{\mathrm{est}}\left[n,m\right]={\mathrm{ISR}}_{\mathrm{est}}\left[n,m\right]\cdot {\left(C/N_{0}\right)}_{\mathrm{Roof}}\left[n,m\right]$$

**Figure 5:**
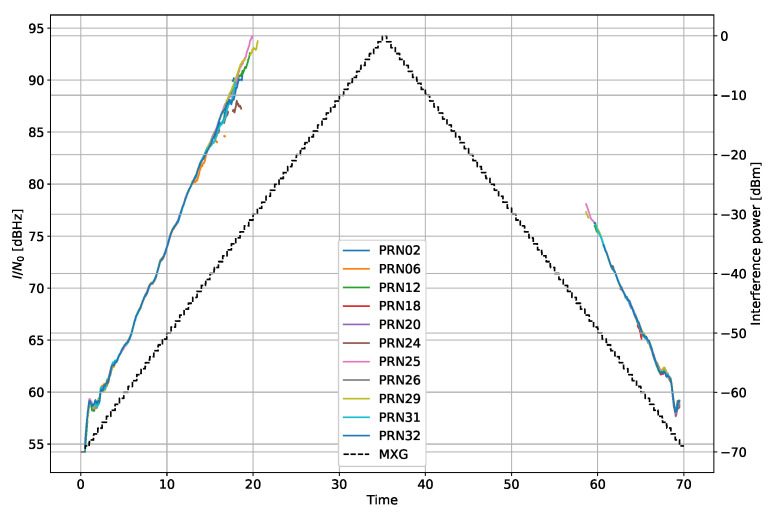
Estimated $I/N_{0}$ from the 4 MHz bandwidth filtered noise interference scenario.

Figure 5 shows the estimates. It is clear that the values are similar between the satellites, and the biases from the ISR are removed.

Fourth, a calibration value is determined to translate from the Agilent MXG signal generator’s output power $P_{t}$ to the $I/N_{0}$. A mean value of all valid calibrations (i.e., invalid tracking data is extracted) determines the final scalar value:

(16) $$\mathrm{CAL}\left[n,m\right]=\frac{{\left(I/N_{0}\right)}_{\mathrm{est}}\left[n,m\right]}{P_{t}\left[n\right]}$$

(17) $$\mathrm{CAL}=\frac{1}{MN}\sum_{n}\sum_{m}\mathrm{CAL}\left[n,m\right]$$

**Figure 6:**
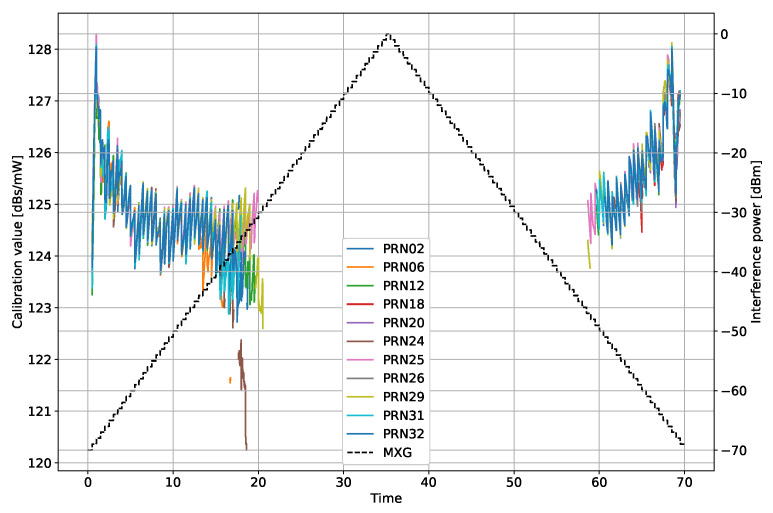
Estimated $I/N_{0}$ from the 4 MHz bandwidth filtered noise interference scenario.

Figure 6 shows the values CAL[n,m] before the mean operation. The mean value is 125.14 dBs/mW and a standard deviation of 0.95 dB, indicating a reliable estimate.

Finally, the calibration CAL is used to translate the Agilent MXG power output $P_{t}$ to the $I/N_{0}$:

(18) $$I/N_{0}\left[n\right]=P_{t}\left[n\right]\cdot \mathrm{CAL}$$

These are the final values shown in the subsequent plots as a reference. The $I/N_{0}$ can be converted to the INR simply by using Equation (12). However, the receiver bandwidths should be known. The LC receivers are believed to have a bandwidth less than 10 MHz and the HE receiver above 50 MHz, but exact values cannot be obtained without the manufacturers disclosing this information. Nevertheless, the INR cannot differ by more than 7 dB depending on the receiver bandwidth, making comparisons challenging. Table 1 compares the output power, INR and $I/N_{0}$, as references.

## 5. Results

The results first show the tracking performance for select cases, then the PVT performance. Finally, the full PVT results are tabled for a detailed comparison.

### 5.1. Tracking Results: C/N0

**Figure 7:**
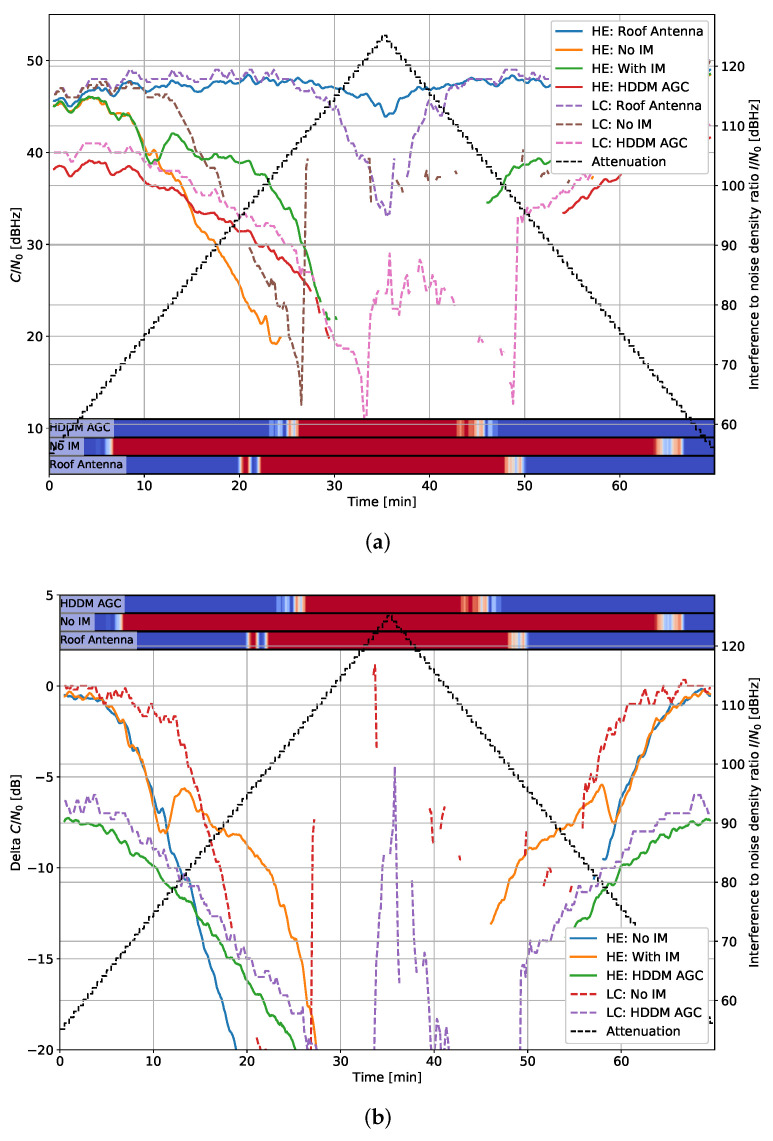
Interference #2 (fast chirp): 10 MHz bandwidth and chirp repetition rate of 10 µs. (**a**) $C/N_{0}$ for the highest elevation satellite. (**b**) Delta $C/N_{0}$ for the highest elevation satellite.

In each plot, the highest elevation satellite is selected to ensure stable tracking over the entire duration of the test. Figure 7 shows the $C/N_{0}$ and the difference of $C/N_{0}$ compared to the roof antenna for each receiver type for the fast chirp. All signals create some V-shape, which correspond to the increase and later decrease in $I/N_{0}$. The HE Roof antenna also has a dip at the highest interference power, indicating that the RF splitter setup described in Figure 3 has limited isolation. The isolation is experimentally determined to be about 45 dB: within the specified 20 to 24 dB values of the RF combiner and splitter. Nevertheless, such results are expected when a large range of $I/N_{0}$s is tested.

A bar indicates whether interference is detected per device in each plot. Blue indicates no detection, and red designates interference is present. Figure 7 detects interference from an $I/N_{0}$ of about 65 dBHz for the “No IM” case. It also detects interference signals in the roof antenna, highlighting the isolation issue again. The detection is less in the HDDM-AGC case. It can be due partly to the IM but also to the AGC that normalizes the spectrum: the detector—amongst other methods—employs an energy detection that requires tuning. The AGC partially counters it, resulting in fewer detections. Nevertheless, the HDDM-AGC still results in some interference detection at high $I/N_{0}$s.

The delta $C/N_{0}$ Figure 7b compares the roof antenna of each receiver type to the other receivers of the same type. Therefore, it shows the loss caused by the interference and mitigation during tracking. The HE with the HDDM-AGC has a loss of 6 dB at low $I/N_{0}$. It is attributed to the quantization noise introduced by the 1-bit DAC used by the R&S up-converter. Unfortunately, it makes the comparison unfair as the HDDM-AGC has a permanent handicap. However, despite this limitation, the HDDM-AGC has comparable or improved IM capabilities in some instances, e.g., between 10 and 14 min. The delta $C/N_{0}$ provides a straightforward receiver comparison, and only these plots are shown in the remainder of this section.

**Figure 8:**
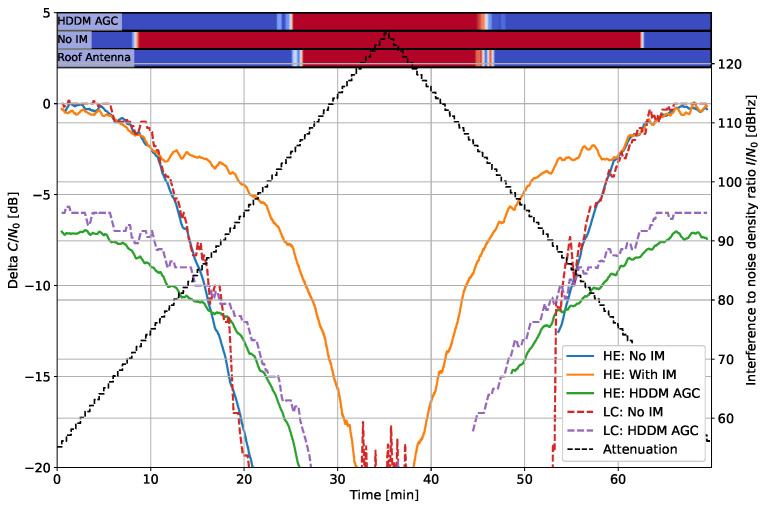
Interference #5: Delta $C/N_{0}$ for fast frequency hopper with 1 µs dwell time and 35 MHz bandwidth.

Figure 8 shows the delta $C/N_{0}$ for the frequency hopper signal. In this case, the HE “with IM” and the HDDM-AGC have a constant offset from the 1-bit DAC, indicating similar IM capabilities.

**Figure 9:**
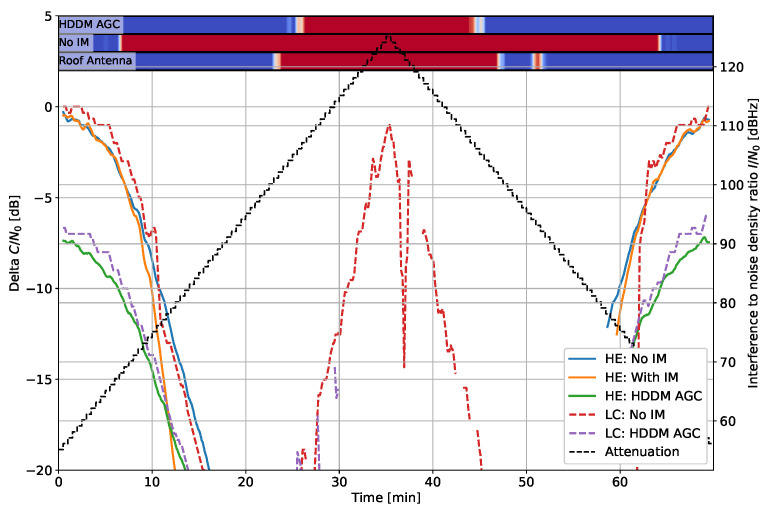
Interference #6: Delta $C/N_{0}$ for filtered noise with 4 MHz bandwidth.

**Figure 10:**
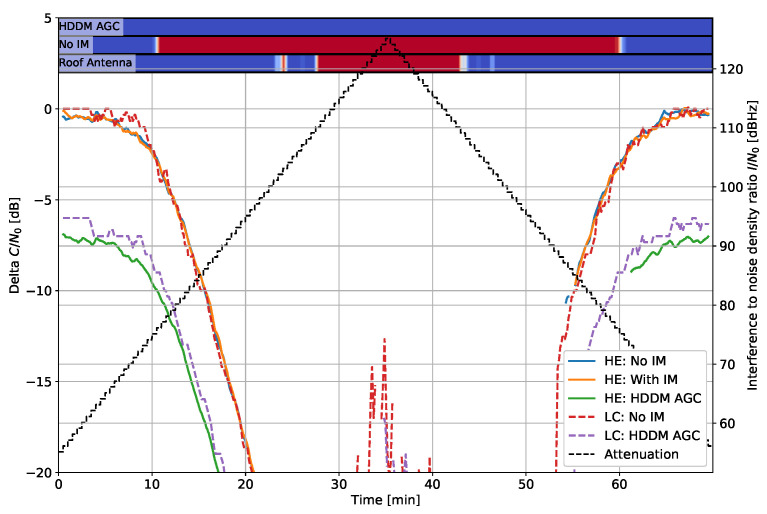
Interference #7: Delta $C/N_{0}$ for filtered noise with 35 MHz bandwidth.

Figure 9 shows the effect to narrow bandwidth noise and Figure 10 for wide-band noise. The interference cannot be mitigated in both cases as it is not sparse in any domain. Hence, any mitigation attempt only results in signal losses. In the narrow bandwidth case of Figure 9, the HDDM-AGC successfully notices the limitation and does not mitigate the signal, and tends to the unmitigated case. The HE “ with IM” tries to mitigate the signal and ultimately cuts out the GNSS signals as well, resulting in inferior performance. Contrarily, in the wide-bandwidth case of Figure 10, all methods recognize that the interference cannot be mitigated, and no losses are perceived. These two cases show that noise interferences are impossible to mitigate if they encompass the GNSS signal of interest, but identifying when to mitigate is the key to minimizing the losses. In Figure 9 the LC “No IM” has almost no loss at high $I/N_{0}$, which can be only explained as an error in the receiver output.

**Figure 11:**
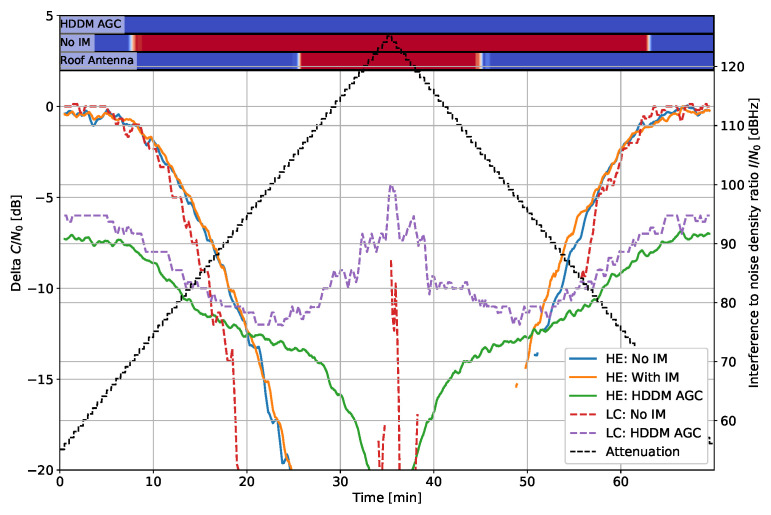
Interference #8: Delta $C/N_{0}$ for pulsed noise with 1 ms pulse width and 35 MHz bandwidth.

**Figure 12:**
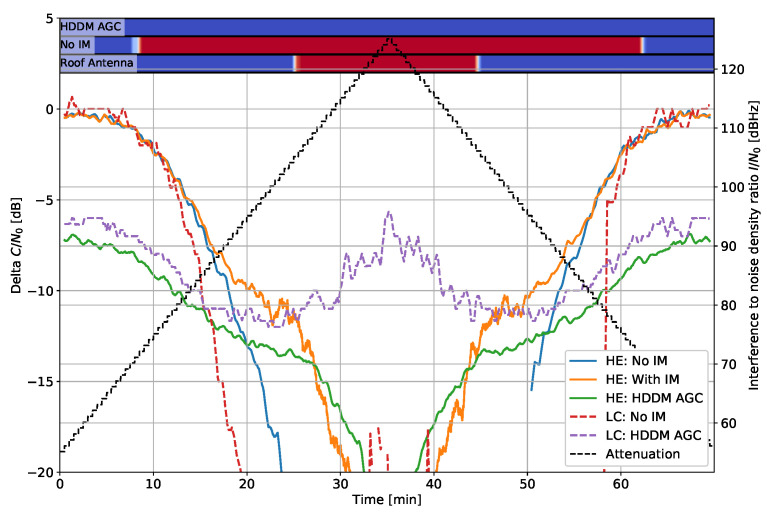
Interference #9: Delta $C/N_{0}$ for pulsed noise with 100 µs pulse width and 35 MHz bandwidth.

Figure 11 shows the response to a slow pulse and Figure 12 for a fast pulse. In both cases, the HDDM-AGC shows improved performance at high $I/N_{0}$. The HE “with IM” responds better for the faster pulse, indicating that it responds best with agile interferences. The pulsed results show that despite the 1-bit DAC, the HDDM-AGC outperforms the state-of-the-art at high $I/N_{0}$s.

### 5.2. Position Results

**Figure 13:**
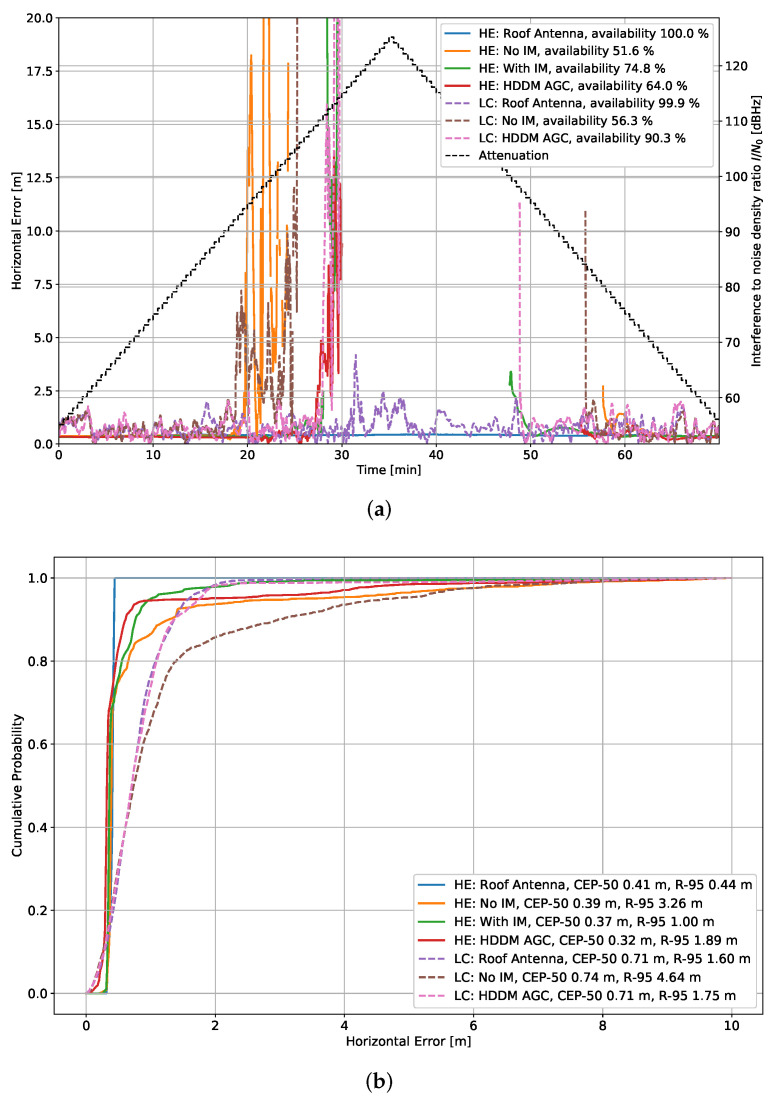
Interference #2 (fast chirp): 10 MHz bandwidth and chirp repetition rate of 10 µs. (**a**) Horizontal error over time. (**b**) Horizontal error CDF.

Figure 13 shows the horizontal position error for the fast chirp tests. First, in Figure 13a, the PVT over time is shown. As the interference power increases, several receivers either result in large position errors, stable error, or give no position output. The first two cases of large or stable errors relate to the internal navigation filter that either drifts away or keeps the last “known” position for as long as possible. However, if the receiver cannot calculate a position sufficiently long, it eventually outputs no PVT solution. Further, the legend in this figure also shows the availability percentage of a PVT, but note that the availability is heavily degraded by the lag introduced by the navigation filters, which output a PVT for several seconds or minutes even though no new PVT could be calculated. Nevertheless, it shows the position performance under different circumstances.

Second, Figure 13b shows the horizontal error cumulative distribution function (CDF) of the available PVT solutions. It indicates how accurate the different receivers are, whether the interference has an impact or not. The legend also displays the 50% circular error probable (CEP) and the R-95 [28].

In this case, the CEP-50 for all receivers is below 75 cm, and the R-95 is below 5 m, indicating that several receivers have problems with outliers resulting from the interference signals. As expected, the best performing receiver is the HE “Roof antenna” and shows an R-95 significantly below 50 cm (it does a stand-alone PVT without additional assistance or corrections save satellite-based augmentation system (SBAS) corrections). Generally, the HE receivers have superior accuracy to the LC ones, as is expected in a geodetic-grade receiver with a superior RFFE, advanced tracking methods, and position filters.

The position plots of the horizontal error over time and the CDF for all interference types are available in the Appendix C for additional reference. The availability percentage of a PVT is shown in Table 2. The HE and LC “Roof antenna” provide both almost 100% for all interference types (there is a single 99.9% availability exception for the LC), showing essentially no outages or issues during the tests. The HE “No IM” had a PVT availability between 30 % and 66 %, whereas the LC had between 50% and 82%. It could be that the LC receiver could keep the navigation filters active for a longer time, which adds some uncertainty to the results. Another reason could be the fact that the LC receiver is a narrow-band receiver, which suppresses out-of-band interference in the analog domain—significantly limiting saturation and degradation effects.

The HE “with IM” (35% to 100% availability) significantly improved the performance in most cases, but the 4 MHz noise decreased the performance. The HDDM-AGC (28% to 100% availability) improved the availability in some cases, e.g., it provided 100% availability for the pulsed and slow chirp signals for both HE and LC. However, in other cases, it degraded the availability, e.g., CW, 4 MHz noise and 35 MHz noise. The most extreme case is with the CW tone signal, but it is known that the HDDM-AGC has issues with CWs [13]. However, the degradation is expected due to the loss of 6 dB $C/N_{0}$ over the up-converter for the two noise interference, making the comparison unfair.

The LC “HDDM” (48% to 100% availability) significantly increased the performance compared to LC “No IM” (51% to 83% availability). However, for the CW and 4 MHz noise interference it degrades, similar to what is observed for the HC. In some cases, e.g., slow frequency hopper and pulsed interference, the HDDM increased the availability of more than 20%.

Table 3 shows the horizontal error probability below 95% R-95. The R-95 is only calculated over the output PVT values. Therefore, if a receiver stays longer in track with degraded tracking conditions, it would have inferior performance to a receiver that stops PVT reporting earlier. Consequently, the results are biased from a scientific perspective but are valid for a practical receiver evaluation.

The HC “Roof antenna” had a 95% error between 26 cm and 55 cm, which is approximately three to eight times better than the LC (1.39 m to 3.48 m). This is expected as it is a geodetic grade receiver with superior processing capabilities but it is also higher size, weight, and power (SWAP).

The HC “No IM” had a R-95 error between 0.42 m and 3.80 m, which is better than “With IM” (0.58 m to 5.12 m) and “HDDM” (1.10 m to 5.63 m). It is unexpected, as it is assumed that mitigation improves performance. A possibility is that the IM degrades the PVT, similar as shown by Borio and Gioia [29]. Another possibility is that the IM results in a larger fraction of PVT solutions being provided at lower $C/N_{0}$s, resulting in larger position errors [26]. A similar observation is found with the LC where the “No IM” (1.70 m to 5.01 m) has superior results to the HDDM (1.75 m to 8.22 m). The HC HDDM had inferior R-95 to the HC “with IM”, and is mainly contributed to the 6 dB loss of the 1-bit DAC: a 6 dB loss in $C/N_{0}$ results in a factor four reduction in PVT precision [26].

## 6. Discussion

The tracking results and PVT results showed that mitigation improved $C/N_{0}$ and availability, respectively. The HDDM has superior mitigation capabilities with pulsed interference, and the AGC corrections had the least degradation with noise interference, showing its benefits in comparison to the state-of-the-art mitigation methods. However, the HDDM has inferior results with single-tone CW signals, but this limitation can be overcome with a notch filter [13]. Therefore, HDDM is a competitive method for IM. Furthermore, the HDDM is implemented on an FPGA running in real-time on a wide-band receiver, indicating the practical application of the method.

The HDDM exhibited fair performance with FMCW signals, such as chirp signals and frequency hoppers, but the comparison is biased by the loss of the 1-bit DAC. Hence, strong conclusions cannot be made, and an improved test setup is proposed for future research. Nevertheless, the HDDM was not significantly worse than the state-of-the-art, even with the handicap. Theoretically, this loss should be about 2 dB from the 1-bit DAC [25]. However, a mean loss of 6 dB is measured. It could be attributed to oscillator leakage from the up-converter (i.e., a strong additional single-tone signal at the intermediate-frequency), which is not properly mitigated by the receivers. Further, the fact that the signal undergoes two RFFE could incur additional losses and deformations of the signal. Therefore, the total loss of 6 dB is within the expectations. However, using an improved DAC and up-converter would significantly improve the setup.

The use of the HDDM-AGC with the LC receiver is especially interesting, as it provides external IM for a GNSS receiver. Therefore, it could provide mitigation capabilities to potentially any interference that does not include IM capabilities.

All receivers were limited to only use GPS L1 C/A and Galileo E1BC signals. Therefore, improved performance may be observed in the PVT if other unaffected signals are also tracked, or if multi-frequency techniques are employed. However, this setup limits a one-to-one comparison of the different receivers and does not represent the full capabilities of either the LC or HC receivers. Other combinations of signals or multi-band interferences could be interesting extensions of the study.

## 7. Conclusions

This paper presents an analysis of the IM performance of the HDDM-AGC method in terms of $C/N_{0}$ and position solution based on high-end geodetic grade and low-cost commercial receivers. For each receiver type, different instances are used, either connected to a clean antenna signal for reference, to a signal with interference, or a signal processed by an HDDM-AGC. To provide a comparison with the state of the art, the built-in mitigation features of the high-end receiver are also enabled in the tests. The HDDM-AGC is implemented on FPGA. The most significant bit of its digital I&Q output is sent to a 1-bit DAC for up-conversion to the L1 band. This way it is used as input for the commercial receivers.

Several interference types are considered in the tests. The results show that the HDDM has competitive mitigation capabilities, but a fair comparison is limited by the physical setup of the study. The most significant limitation of this study is the 1-bit DAC, which resulted in an unfair comparison of the HDDM performance. Therefore, it is suggested to repeat this study with an improved DAC for external comparisons to limit unnecessary losses. Nevertheless, the HDDM showed competitive mitigation capabilities, despite this limitation. Furthermore, the results also showed degradation in PVT accuracy using IM (although availability improved), an aspect that requires more investigations.

## Figures and Tables

**Table 1:** Comparison of interference power metrics.

Metric	Unit	Min Value	Max Value
Signal generator output power $P_{t}$	dBm	−70	0
$I/N_{0}$	dBHz	51.54	121.54
INR for a 10 MHz bandwidth receiver	dB	−18.46	51.54
INR for a 50 MHz bandwidth receiver	dB	−25.45	44.55

**Table 2:** Percent availability.

Interference	High-End (HE)				Low-Cost (LC)		
Number	Roof	No IM	With IM	HDDM	Roof	No IM	HDDM
#1 CW	100	30.5	100	28.7	100	71.1	68.9
#2 Fast Chirp	100	51.6	74.8	64.0	99.9	56.3	90.3
#3 Slow Chirp	100	35.5	75.5	72.9	100	81.1	90.7
#4 Fast Hop	100	59.5	100	70.5	100	83.0	84.2
#5 Slow Hop	100	58.0	100	67.4	100	75.3	97.4
#6 4 MHz Noise	100	43.5	35.3	40.0	100	51.0	48.5
#7 35 MHz Noise	100	56.5	57.5	43.8	100	82.3	83.9
#8 Slow Pulse	100	65.6	66.8	100	100	77.5	100
#9 Fast Pulse	100	64.4	86.8	100	100	60.4	99.7
Min. #1 to #9	100	30.5	35.3	28.7	99.9	51.0	48.5
Mean #1 to #9	100	51.7	77.4	65.3	100	70.9	84.8
Max. #1 to #9	100	65.6	100	100	100	83.0	100

**Table 3:** 95% Horizontal position error R-95.

Interference	High-End (HE)				Low-Cost (LC)		
Number	Roof	No IM	With IM	HDDM	Roof	No IM	HDDM
#1 CW	0.43	0.42	0.58	1.49	1.56	2.42	8.22
#2 Fast Chirp	0.44	3.26	1.00	1.89	1.60	4.64	1.75
#3 Slow Chirp	0.47	1.01	0.67	1.10	2.37	2.12	3.22
#4 Fast Hop	0.26	2.07	3.76	2.14	1.68	2.12	3.71
#5 Slow Hop	0.26	1.48	5.12	2.10	1.82	2.38	3.01
#6 4 MHz Noise	0.55	3.80	0.73	2.61	3.48	5.01	4.07
#7 35 MHz Noise	0.30	1.37	1.91	3.44	1.73	3.78	3.61
#8 Slow Pulse	0.35	1.79	1.72	2.78	1.39	1.70	4.98
#9 Fast Pulse	0.42	2.50	1.14	5.63	1.61	2.49	3.99
Min. #1 to #9	0.26	0.42	0.58	1.10	1.39	1.70	1.75
Mean #1 to #9	0.39	1.97	1.85	2.58	1.92	2.96	4.06
Max. #1 to #9	0.55	3.80	5.12	5.63	3.48	5.01	8.22

**Figure A1:**
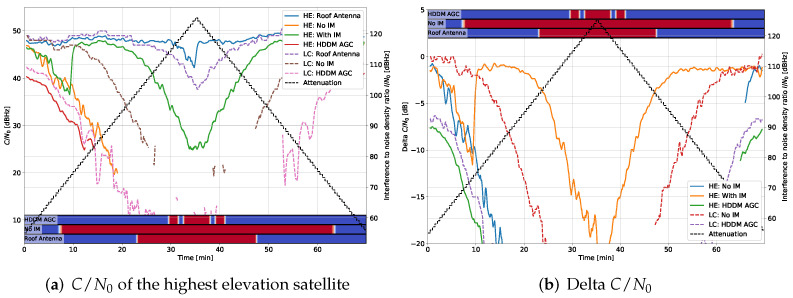
Interference #1: CW 1 575.42 MHz with GPS L1/CA.

**Figure A2:**
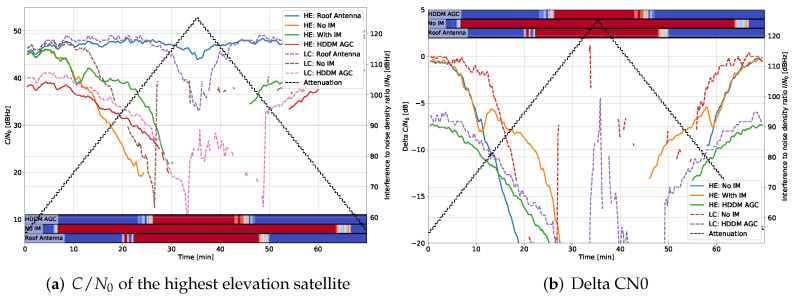
Interference #2: Fast Chirp 10 MHz with GPS L1/CA.

**Figure A3:**
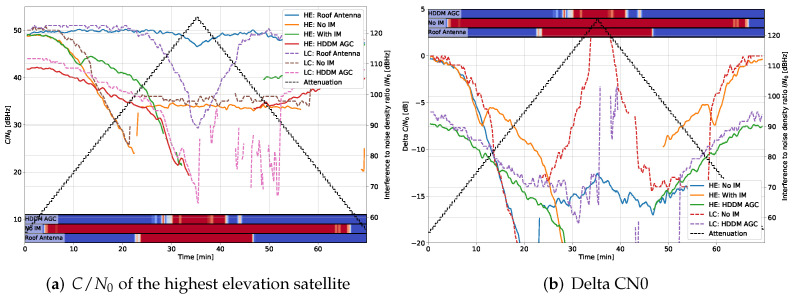
Interference #3: Slow Chirp 10 MHz with GPS L1/CA.

**Figure A4:**
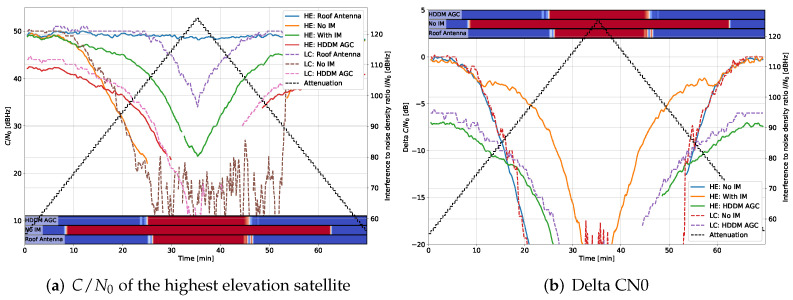
Interference #4: Fast frequency hopper with a dwell time of 1 us and a frequency range of 35 MHz with GPS L1/CA.

**Figure A5:**
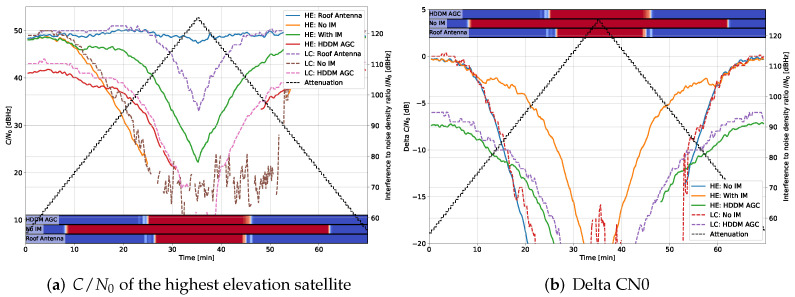
Interference #5: Slow frequency hopper with a dwell time of 100 us and a frequency range of 35 MHz with GPS L1/CA.

**Figure A6:**
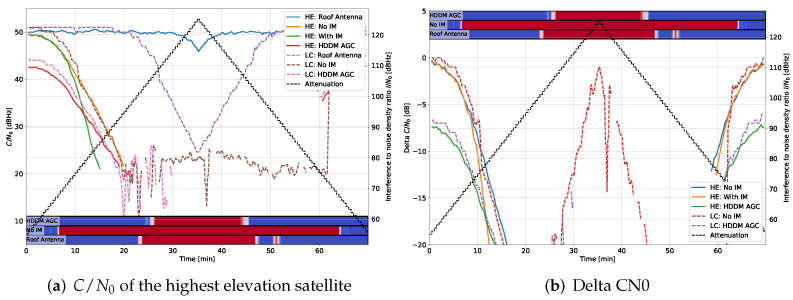
Interference #6: Filtered noise with 4 MHz bandwidth with GPS L1/CA.

**Figure A7:**
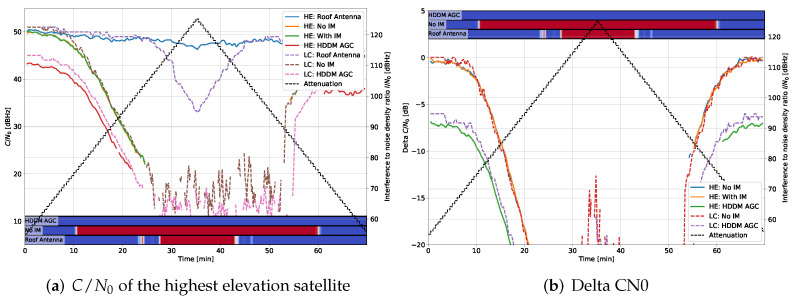
Interference #7: Filtered noise with 35 MHz bandwidth with GPS L1/CA.

**Figure A8:**
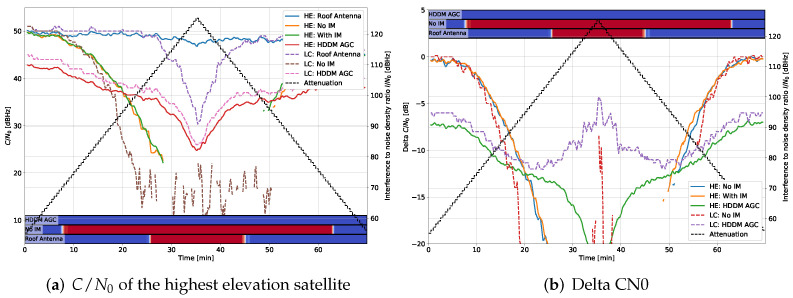
Interference #8: Filtered pulsed noise with 35 MHz bandwidth, 50% duty cycle, and 1 ms pulse width with GPS L1/CA.

**Figure A9:**
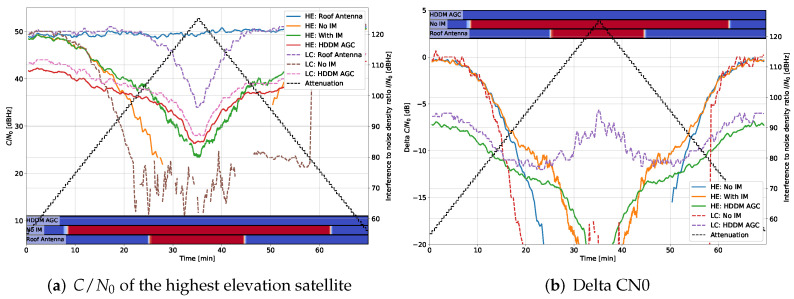
Interference #9: Filtered pulsed noise with 35 MHz bandwidth, 50% duty cycle, and 100 us pulse width with GPS L1/CA.

**Figure A10:**
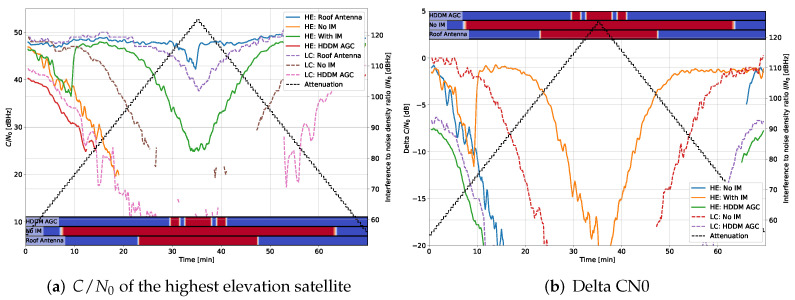
Interference #1: CW 1 575.42 MHz with Galileo E1BC.

**Figure A11:**
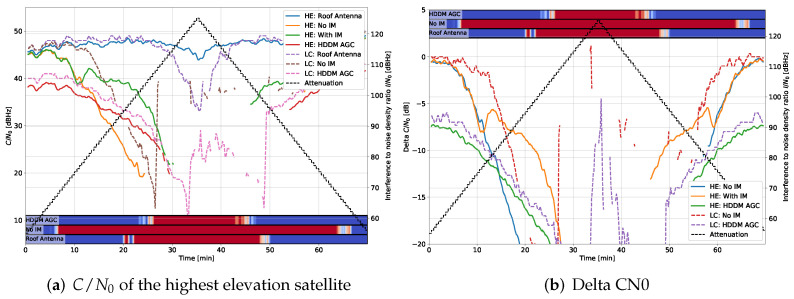
Interference #2: Fast Chirp 10 MHz with Galileo E1BC.

**Figure A12:**
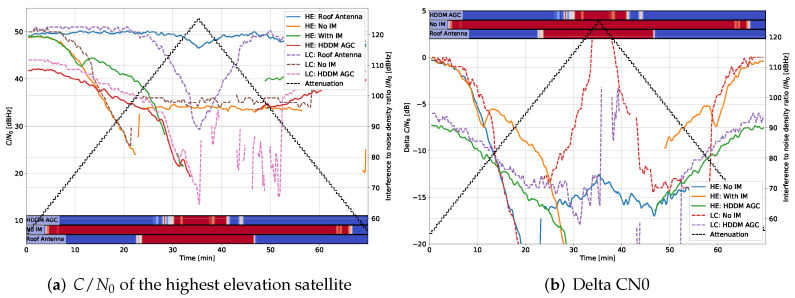
Interference #3: Slow Chirp 10 MHz with Galileo E1BC.

**Figure A13:**
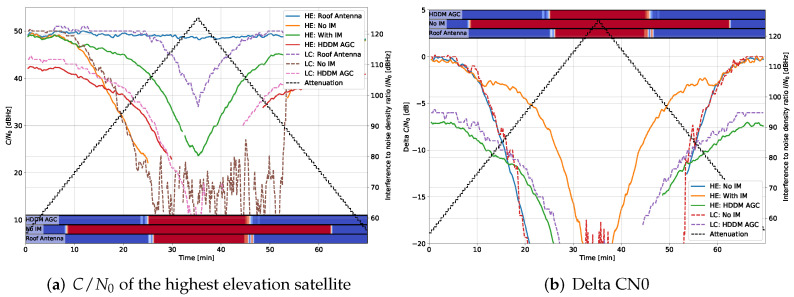
Interference #4: Fast frequency hopper with a dwell time of 1 us and a frequency range of 35 MHz with Galileo E1BC.

**Figure A14:**
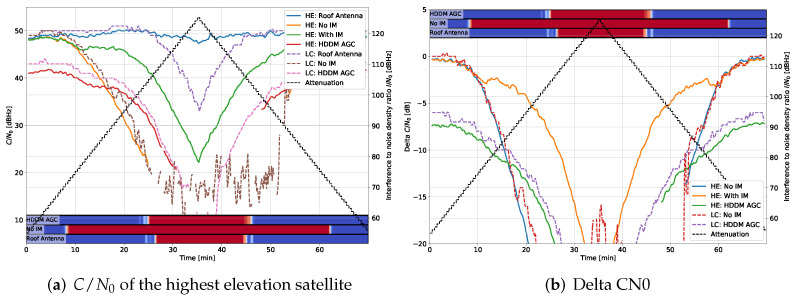
Interference #5: Slow frequency hopper with a dwell time of 100 us and a frequency range of 35 MHz with Galileo E1BC.

**Figure A15:**
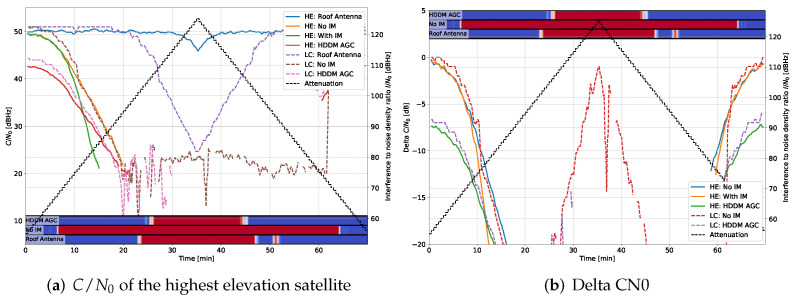
Interference #6: Filtered noise with 4 MHz bandwidth with Galileo E1BC.

**Figure A16:**
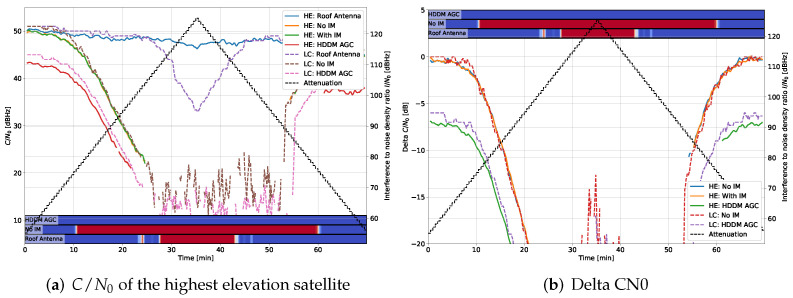
Interference #7: Filtered noise with 35 MHz bandwidth with Galileo E1BC.

**Figure A17:**
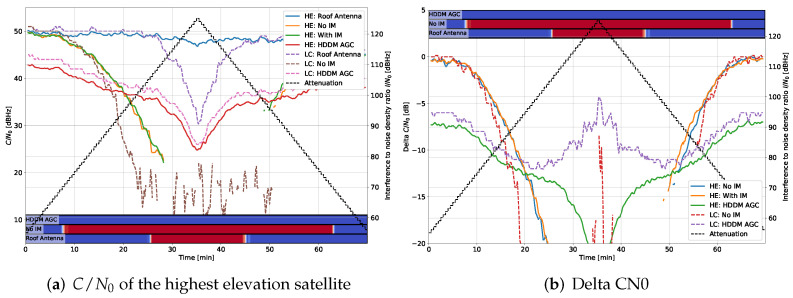
Interference #8: Filtered pulsed noise with 35 MHz bandwidth, 50% duty cycle, and 1 ms pulse width with Galileo E1BC.

**Figure A18:**
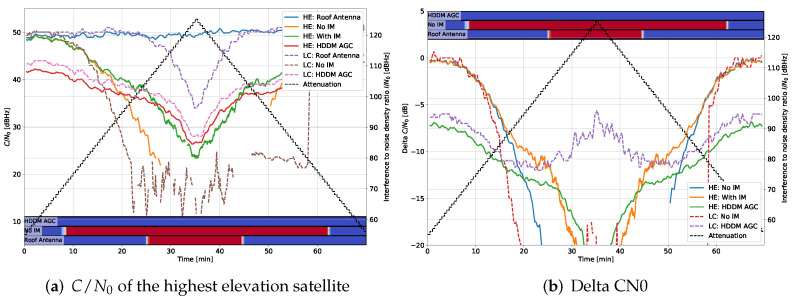
Interference #9: Filtered pulsed noise with 35 MHz bandwidth, 50% duty cycle, and 100 us pulse width with Galileo E1BC.

**Figure A19:**
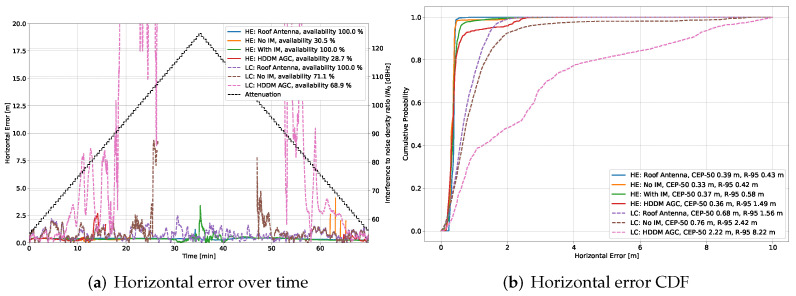
Interference #1: CW 1 575.42 MHz.

**Figure A20:**
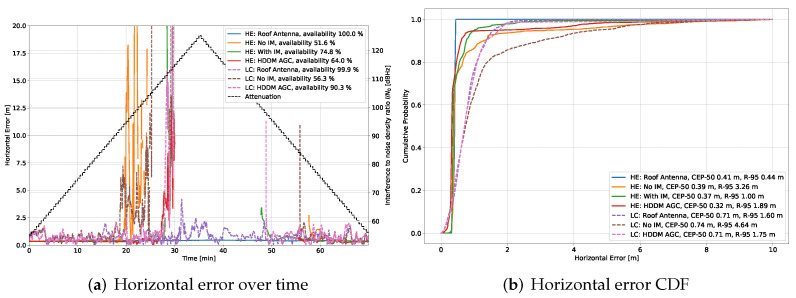
Interference #2: Fast Chirp 10 MHz.

**Figure A21:**
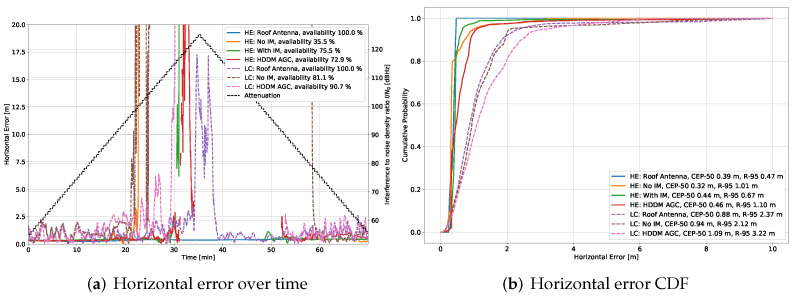
Interference #3: Slow Chirp 10 MHz.

**Figure A22:**
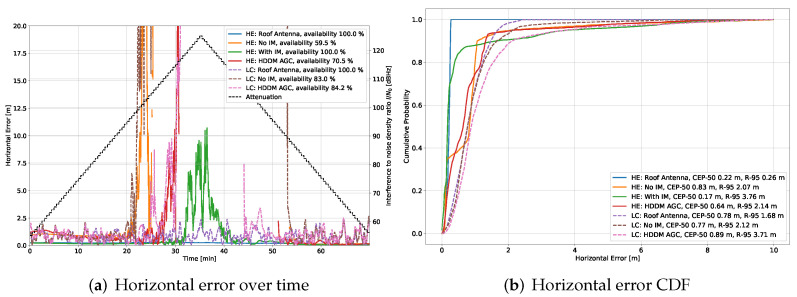
Interference #4: Fast frequency hopper with a dwell time of 1 us and a frequency range of 35 MHz.

**Figure A23:**
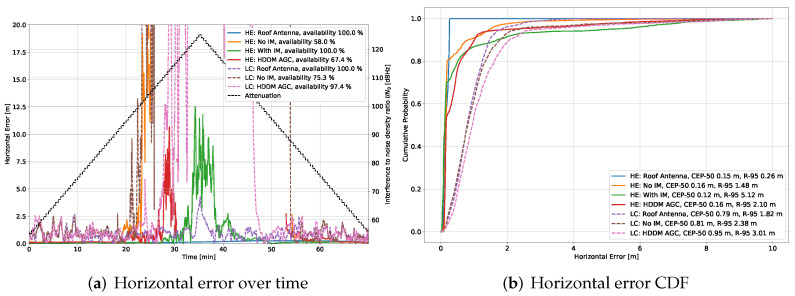
Interference #5: Slow frequency hopper with a dwell time of 100 us and a frequency range of 35 MHz.

**Figure A24:**
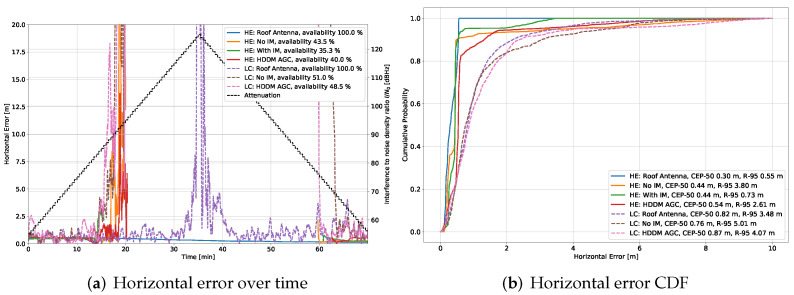
Interference #6: Filtered noise with 4 MHz bandwidth.

**Figure A25:**
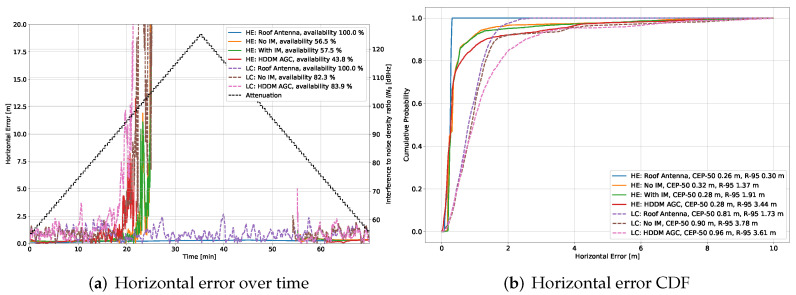
Interference #7: Filtered noise with 35 MHz bandwidth.

**Figure A26:**
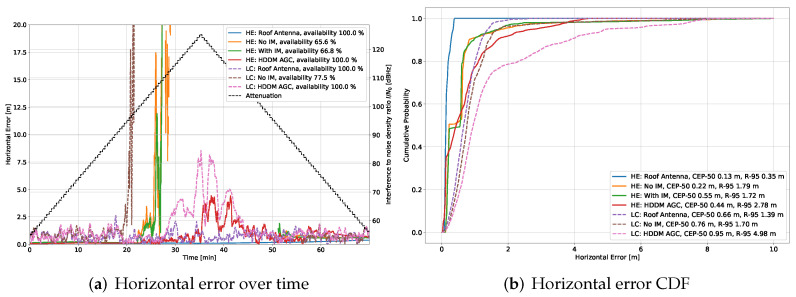
Interference #8: Filtered pulsed noise with 35 MHz bandwidth, 50% duty cycle, and 1 ms pulse width.

**Figure A27:**
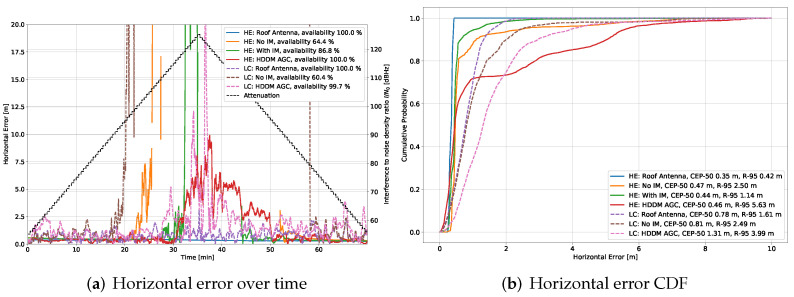
Interference #9: Filtered pulsed noise with 35 MHz bandwidth, 50% duty cycle, and 100 us pulse width.

## Data Availability

The full results of all SVIDs analyzed in this study are available as Supplementary Material to the article.

## Supplementary Materials

The following supporting information can be downloaded at: https://www.mdpi.com/article/10.3390/s22020679/s1.

## Abbreviations

The following abbreviations are used in this manuscript:

$C/N_{0}$	carrier-to-noise density ratio
$I/N_{0}$	interference-to-noise density ratio
AGC	automatic gain control
ANF	adaptive notch filtering
ASIC	application-specific integrated circuit
CDF	cumulative distribution function
CDMA	code-division multiple access
CEP	circular error probable
COTS	commercial-off-the-shelf
CW	continuous-wave
DAC	digital-to-analog converter
DFT	discrete Fourier transform
DSP	digital signal processor
DVB-T	digital video broadcasting – terrestrial
DWT	discrete wavelet transform
FDAF	frequency-domain adaptive filtering
FFT	fast Fourier transform
FMCW	frequency-modulated continuous-wave
FPGA	field-programmable gate array
GNSS	global navigation satellite system
HDDM	high-rate DFT-based data manipulator
HE	high-end
HW	hardware
IDFT	inverse discrete Fourier transform
IFFT	inverse fast Fourier transform
IM	interference mitigation
INR	interference-to-noise ratio
IP	intellectual property
ISR	interference-to-signal ratio
KLT	Karhunen-Loève transform
LC	low-cost
ML	machine learning
PB	pulse blanker
PSD	power spectral density
PVT	position, velocity, and time
RF	radio-frequency
RFFE	radio-frequency front-end
RTL	register-transfer level
SBAS	satellite-based augmentation system
SSC	spectral separation coefficient
SWAP	size, weight, and power
VHDL	very-high-speed integrated circuit hardware description language
