# Supplementary figures and images for: Wide-Band Interference Mitigation in GNSS Receivers Using Sub-Band Automatic Gain Control (part 1 of 2)
Source: Sensors (Basel). 2022 Jan 16;22(2):679. doi: 10.3390/s22020679 (PMC8779687; doi:10.3390/s22020679)

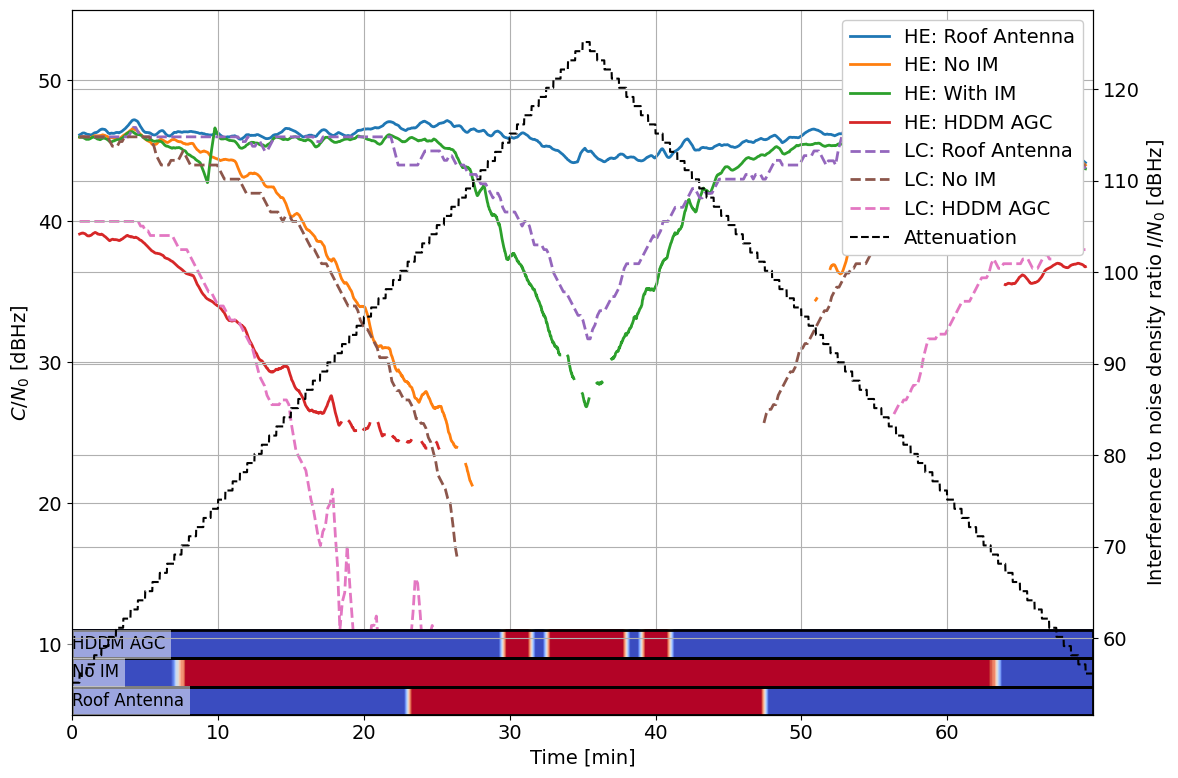

Supplement: Supplementary file 1 [file sensors-22-00679-s001.zip › results/Galileo/E1BC/15754200/HELC_Bar_SVID2_CN0.png]

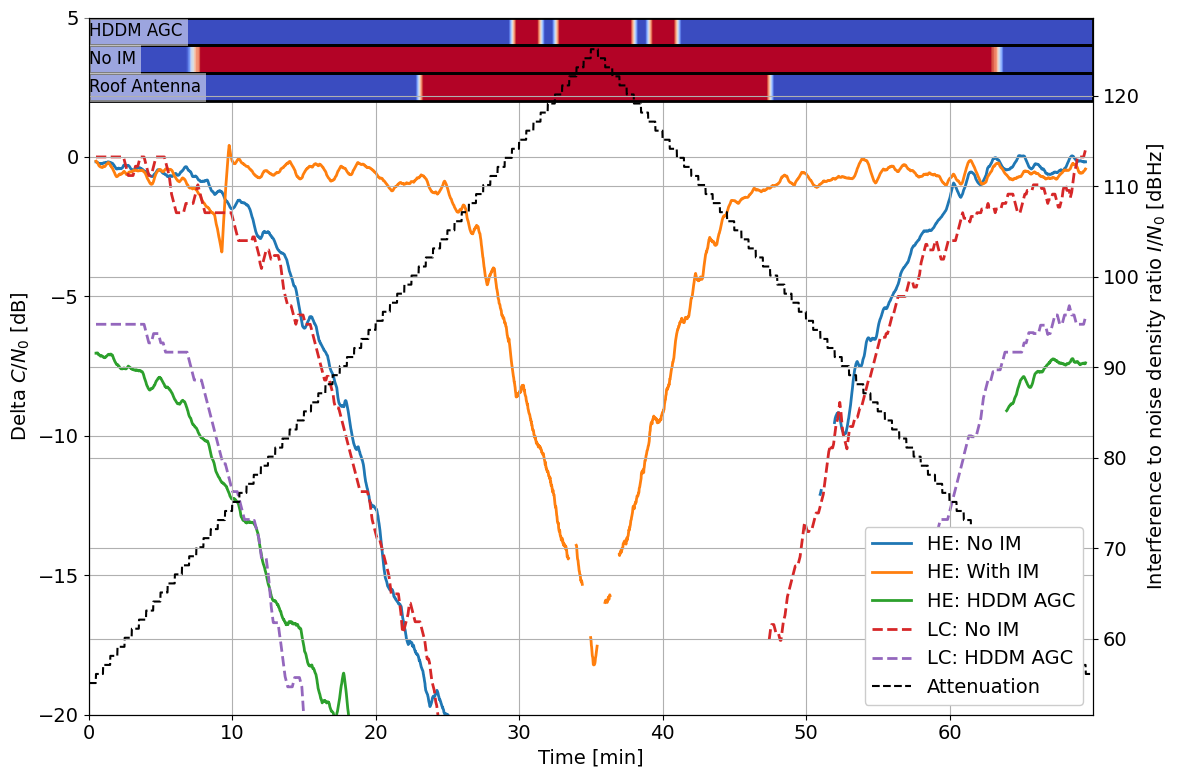

Supplement: Supplementary file 1 [file sensors-22-00679-s001.zip › results/Galileo/E1BC/15754200/HELC_Bar_SVID2_DeltaCN0.png]

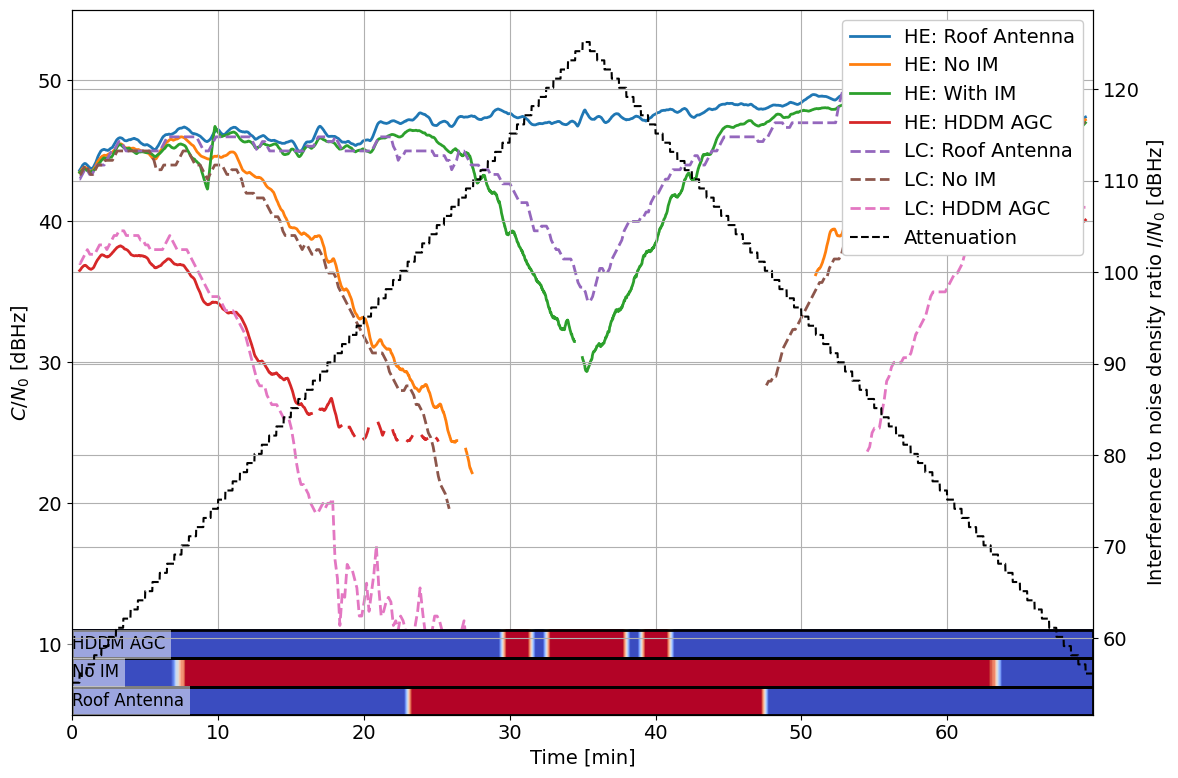

Supplement: Supplementary file 1 [file sensors-22-00679-s001.zip › results/Galileo/E1BC/15754200/HELC_Bar_SVID30_CN0.png]

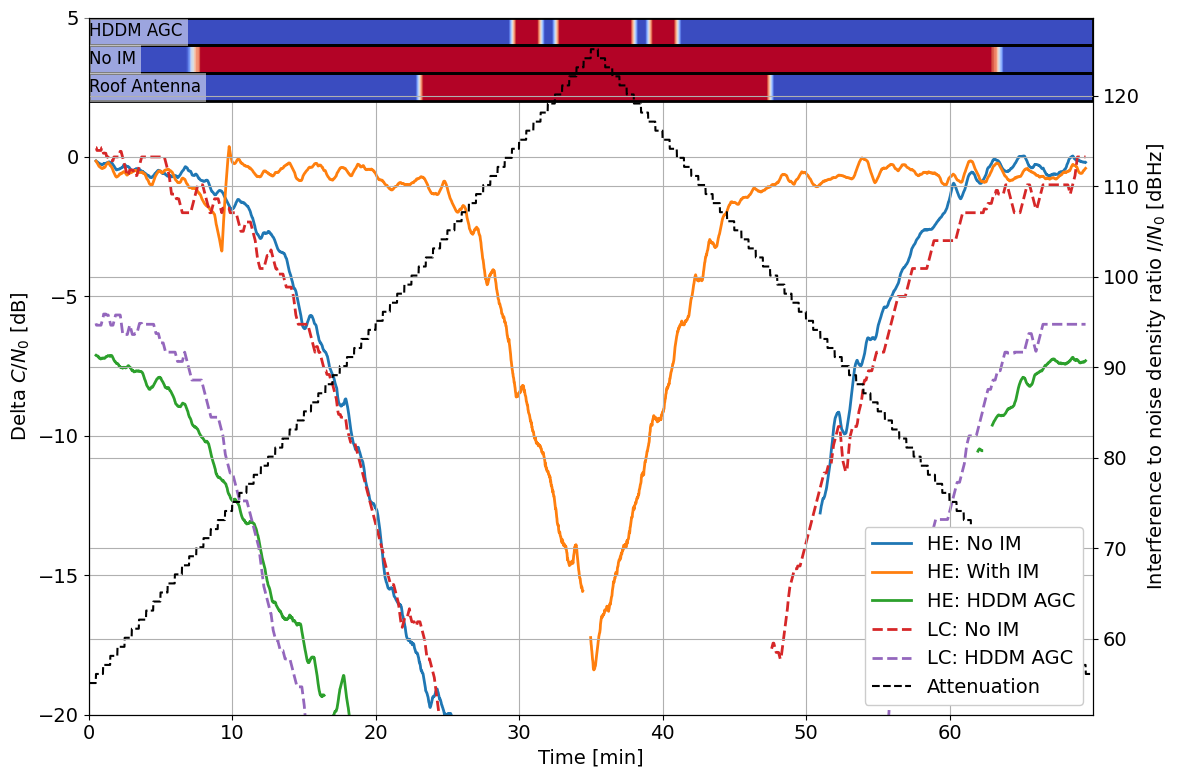

Supplement: Supplementary file 1 [file sensors-22-00679-s001.zip › results/Galileo/E1BC/15754200/HELC_Bar_SVID30_DeltaCN0.png]

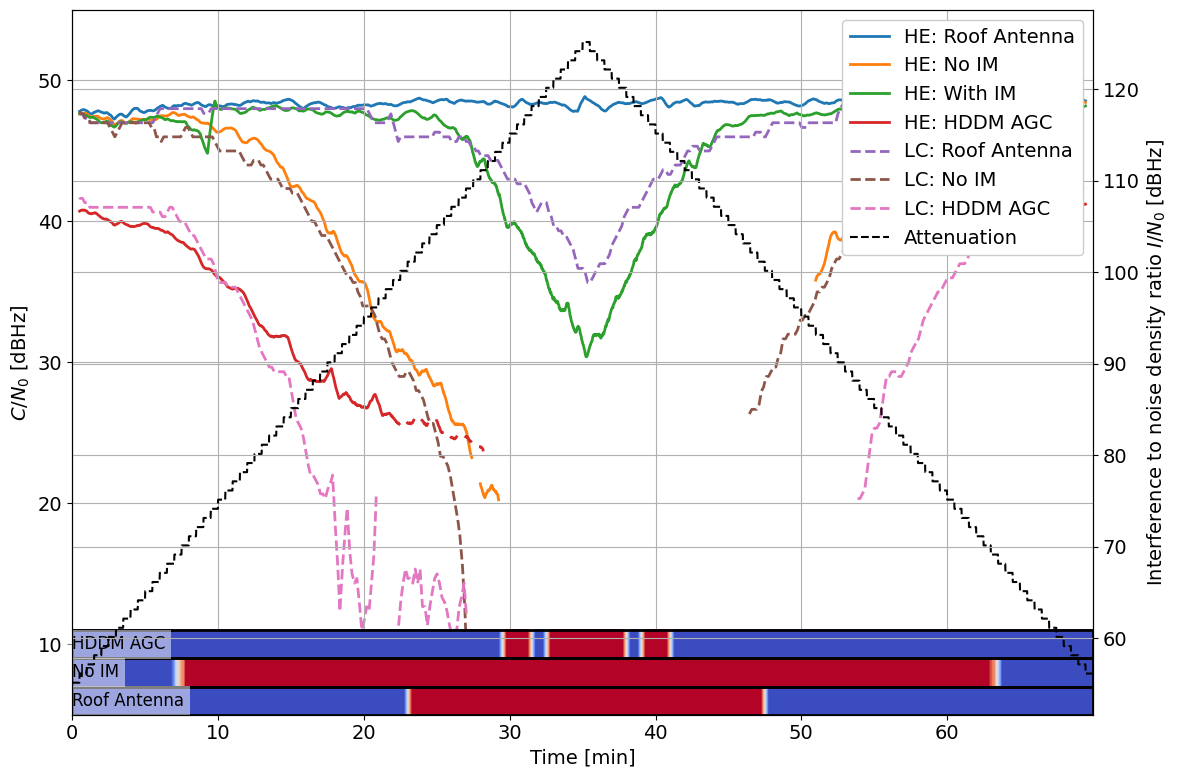

Supplement: Supplementary file 1 [file sensors-22-00679-s001.zip › results/Galileo/E1BC/15754200/HELC_Bar_SVID7_CN0.png]

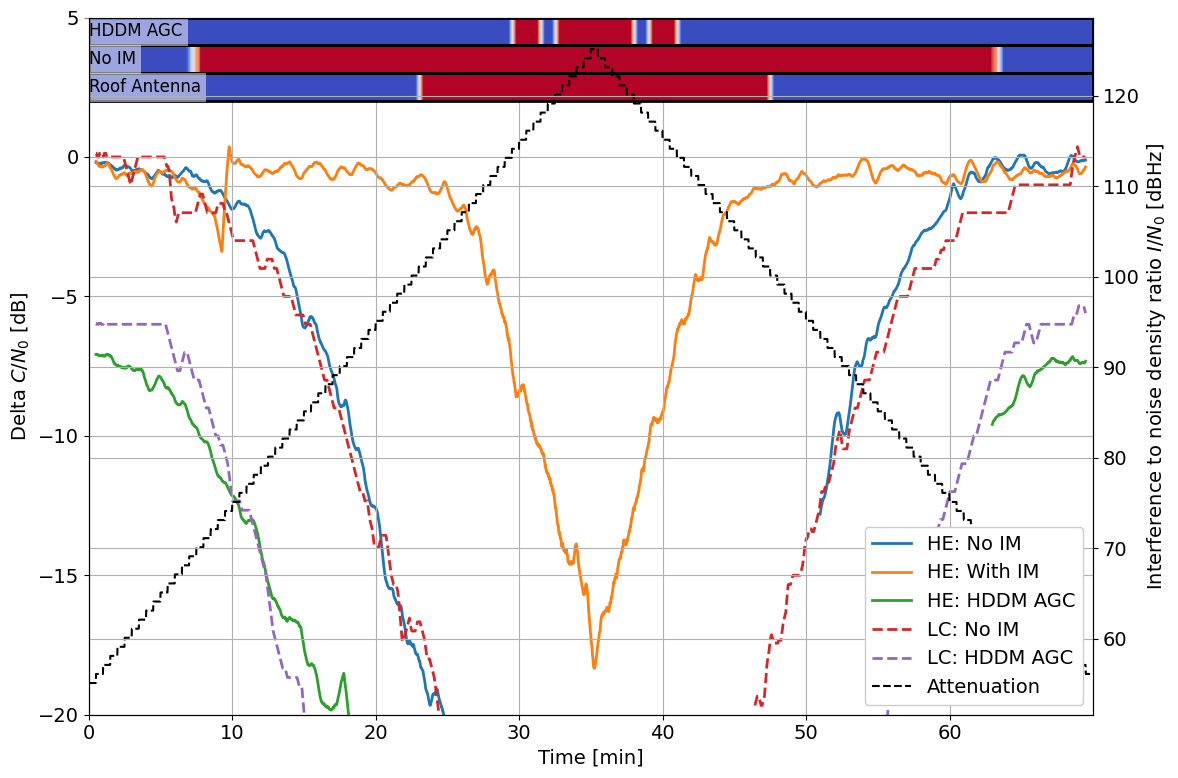

Supplement: Supplementary file 1 [file sensors-22-00679-s001.zip › results/Galileo/E1BC/15754200/HELC_Bar_SVID7_DeltaCN0.png]

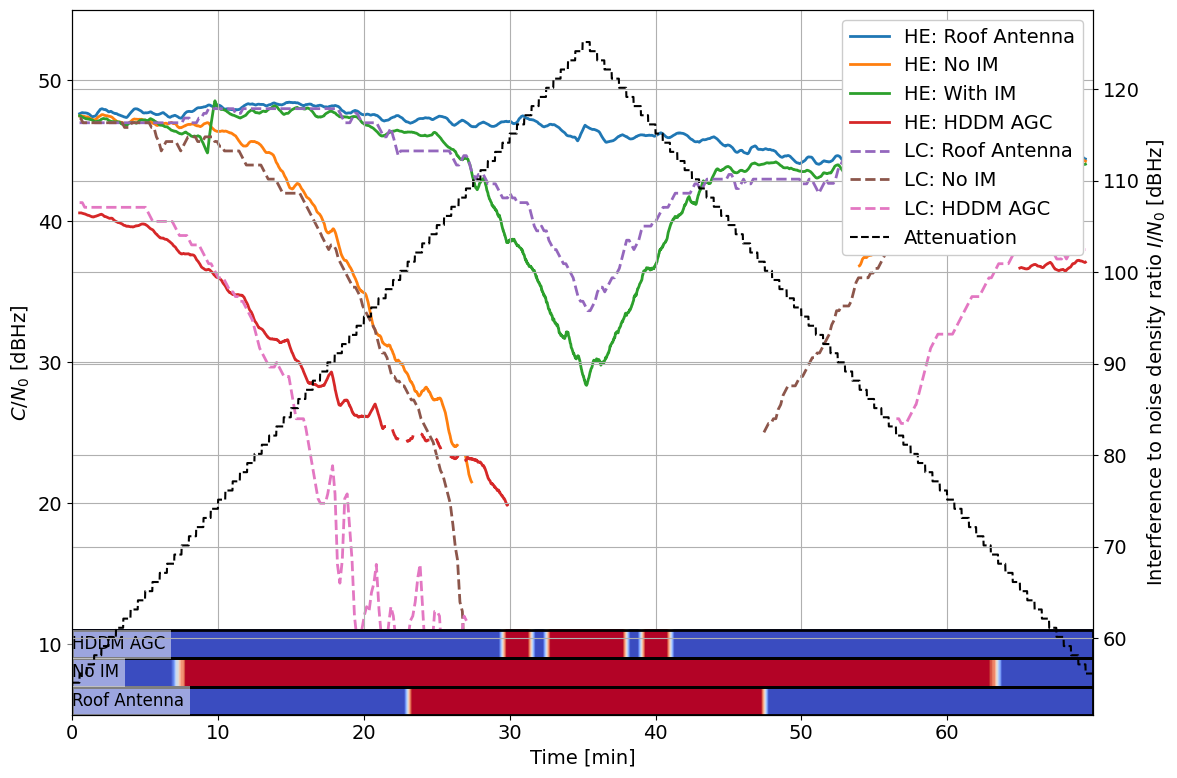

Supplement: Supplementary file 1 [file sensors-22-00679-s001.zip › results/Galileo/E1BC/15754200/HELC_Bar_SVID8_CN0.png]

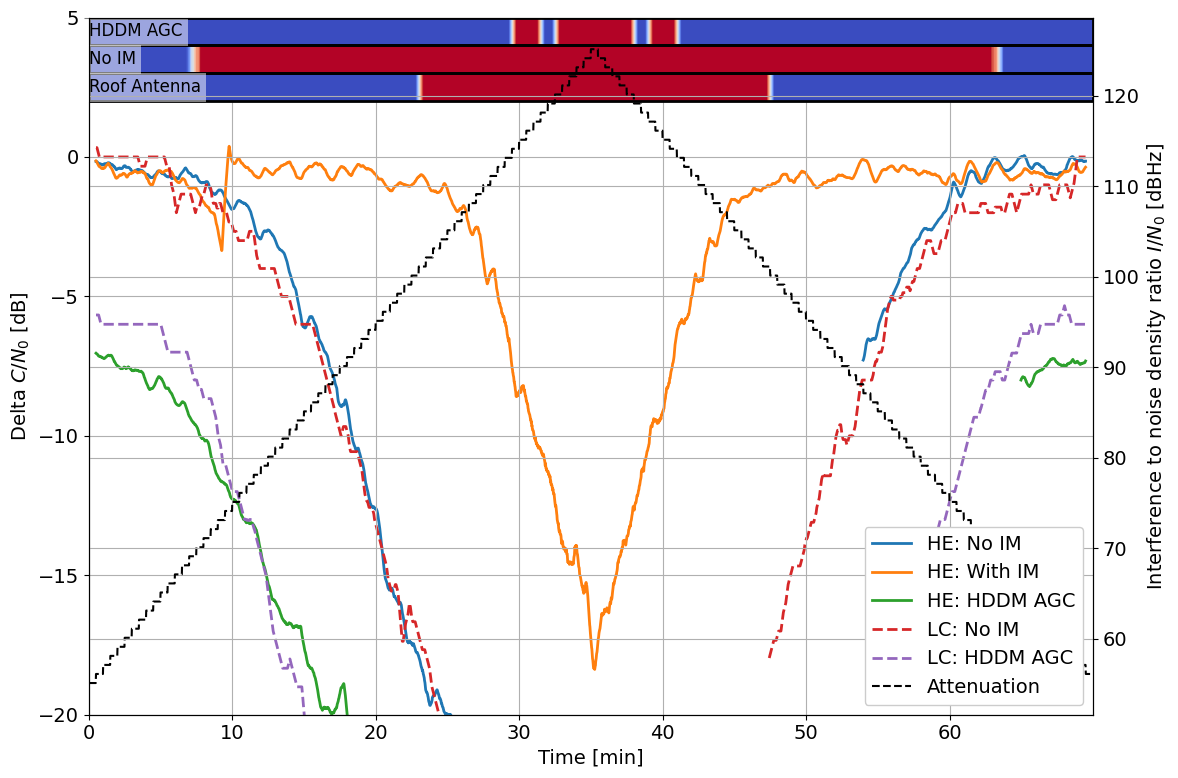

Supplement: Supplementary file 1 [file sensors-22-00679-s001.zip › results/Galileo/E1BC/15754200/HELC_Bar_SVID8_DeltaCN0.png]

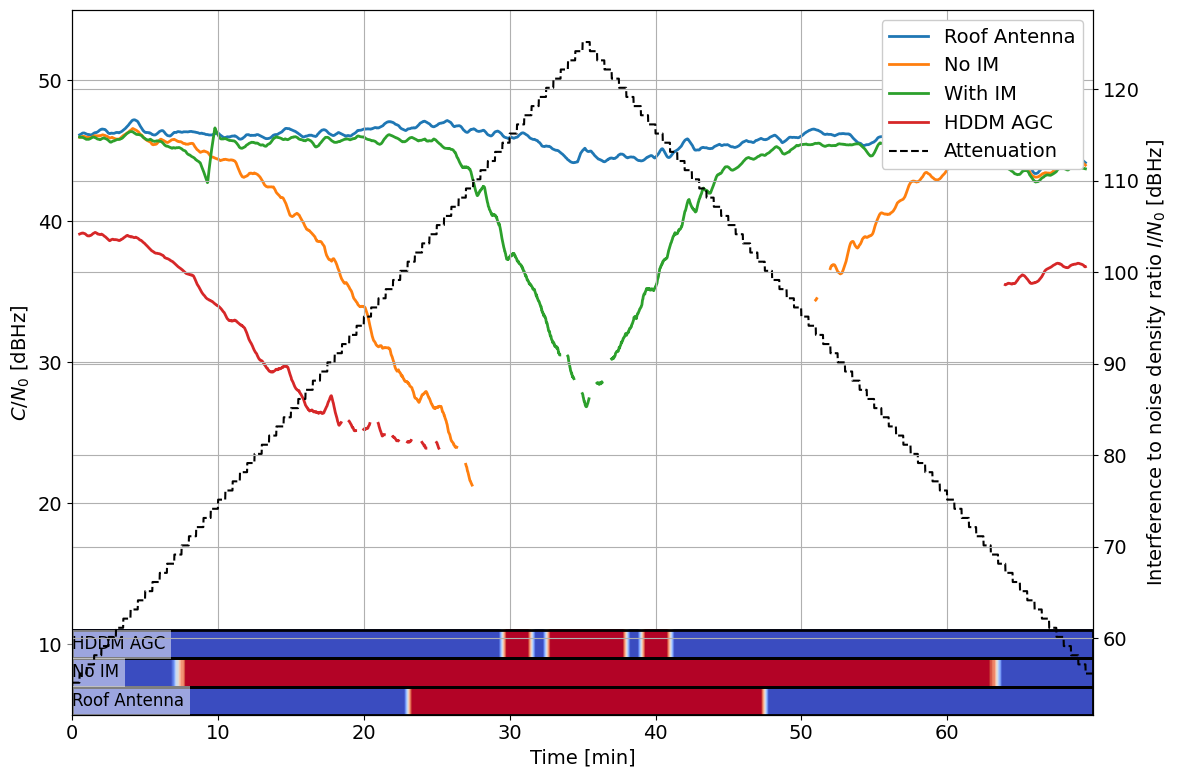

Supplement: Supplementary file 1 [file sensors-22-00679-s001.zip › results/Galileo/E1BC/15754200/HE_Bar_SVID2_CN0.png]

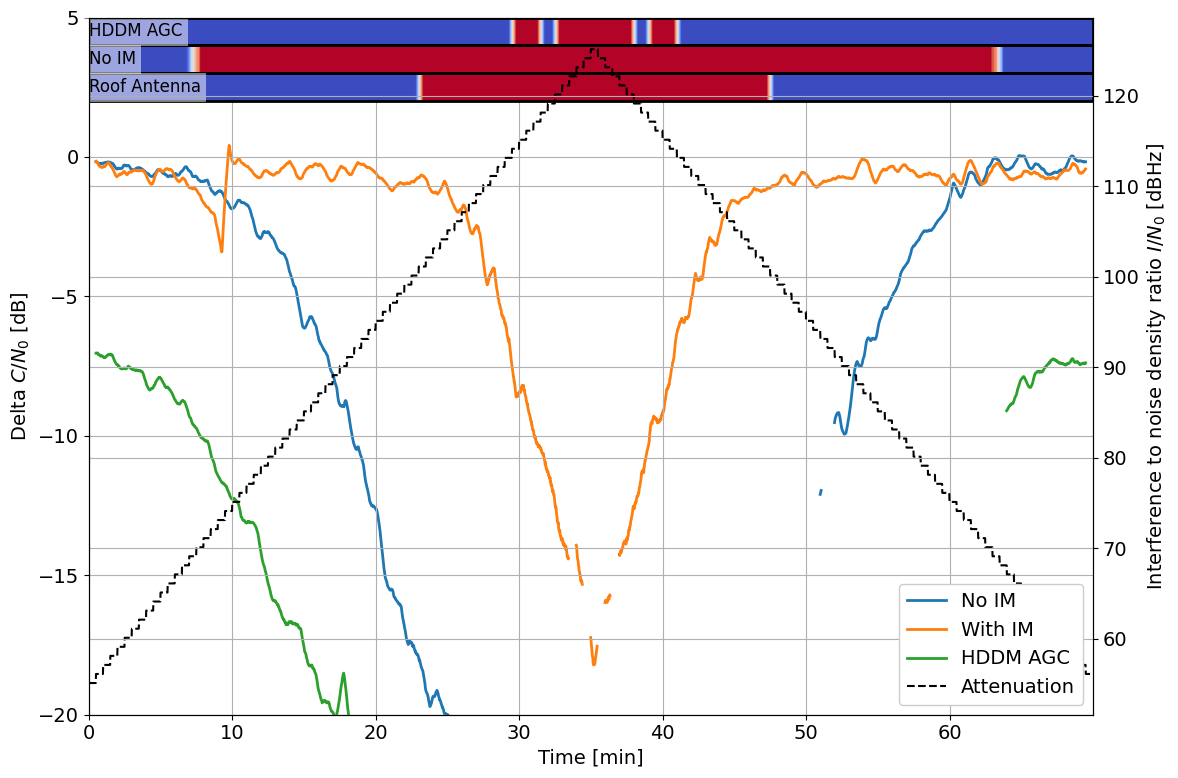

Supplement: Supplementary file 1 [file sensors-22-00679-s001.zip › results/Galileo/E1BC/15754200/HE_Bar_SVID2_DeltaCN0.png]

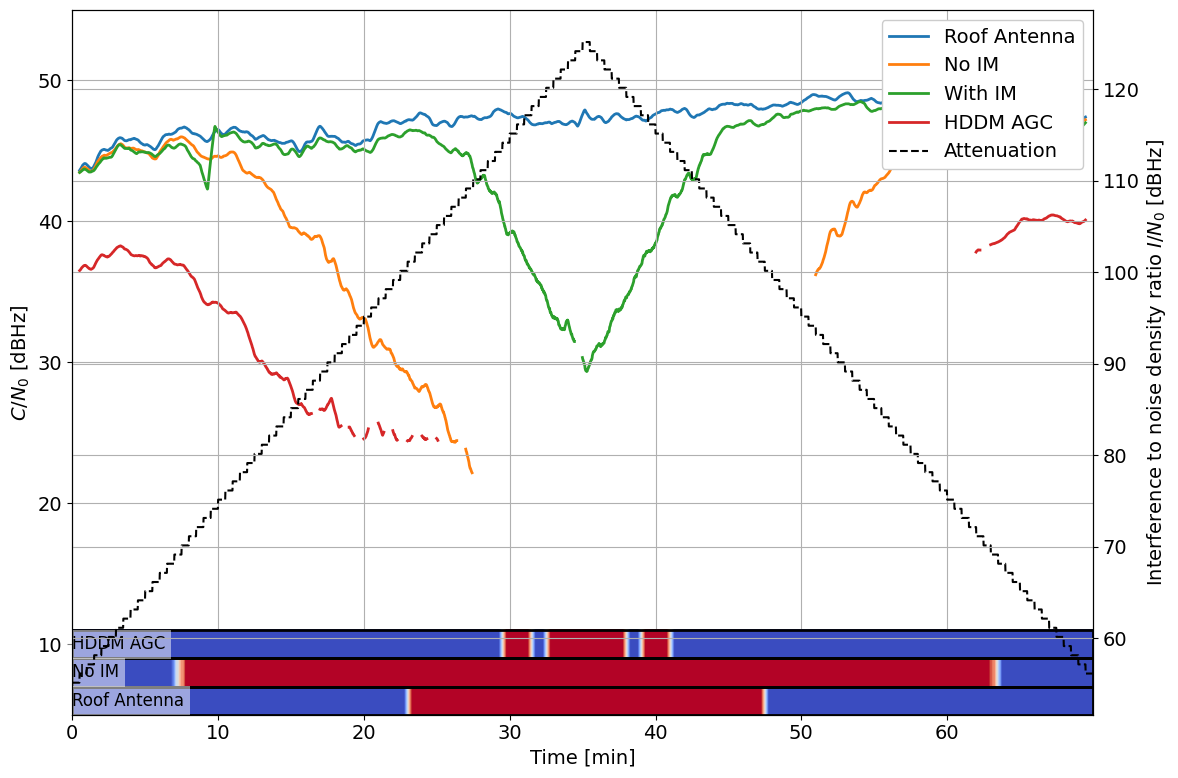

Supplement: Supplementary file 1 [file sensors-22-00679-s001.zip › results/Galileo/E1BC/15754200/HE_Bar_SVID30_CN0.png]

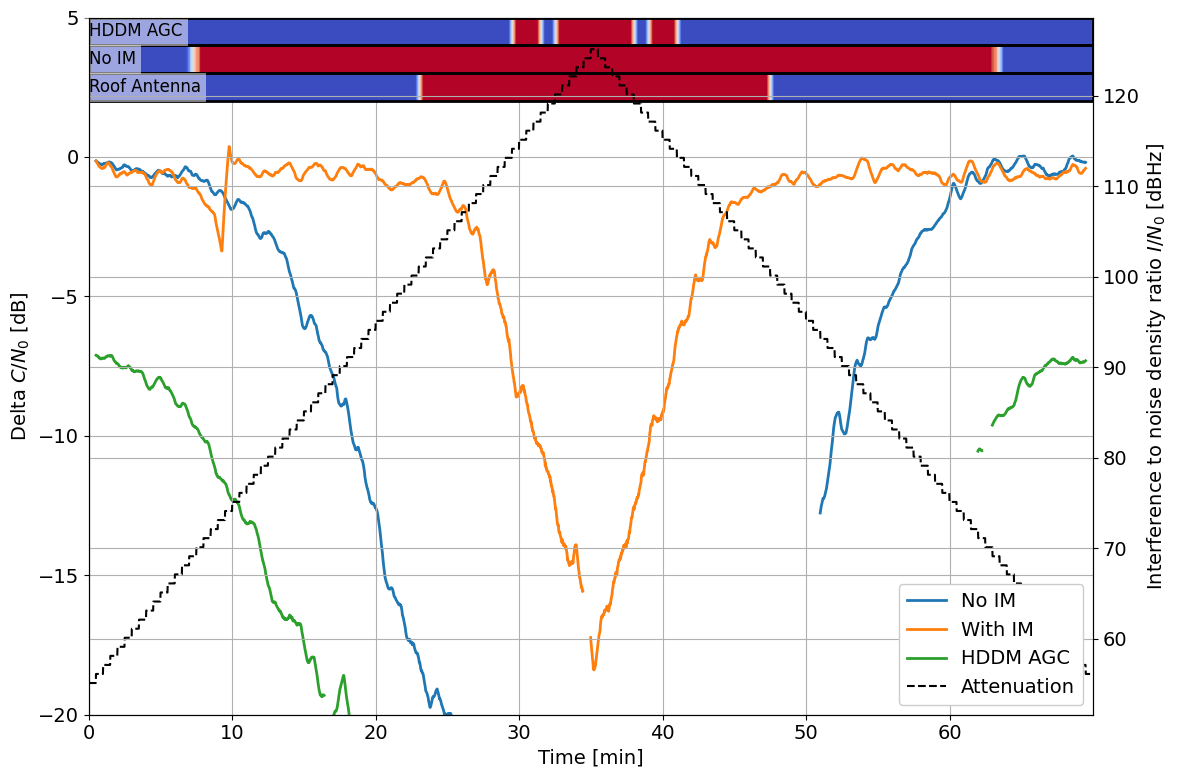

Supplement: Supplementary file 1 [file sensors-22-00679-s001.zip › results/Galileo/E1BC/15754200/HE_Bar_SVID30_DeltaCN0.png]

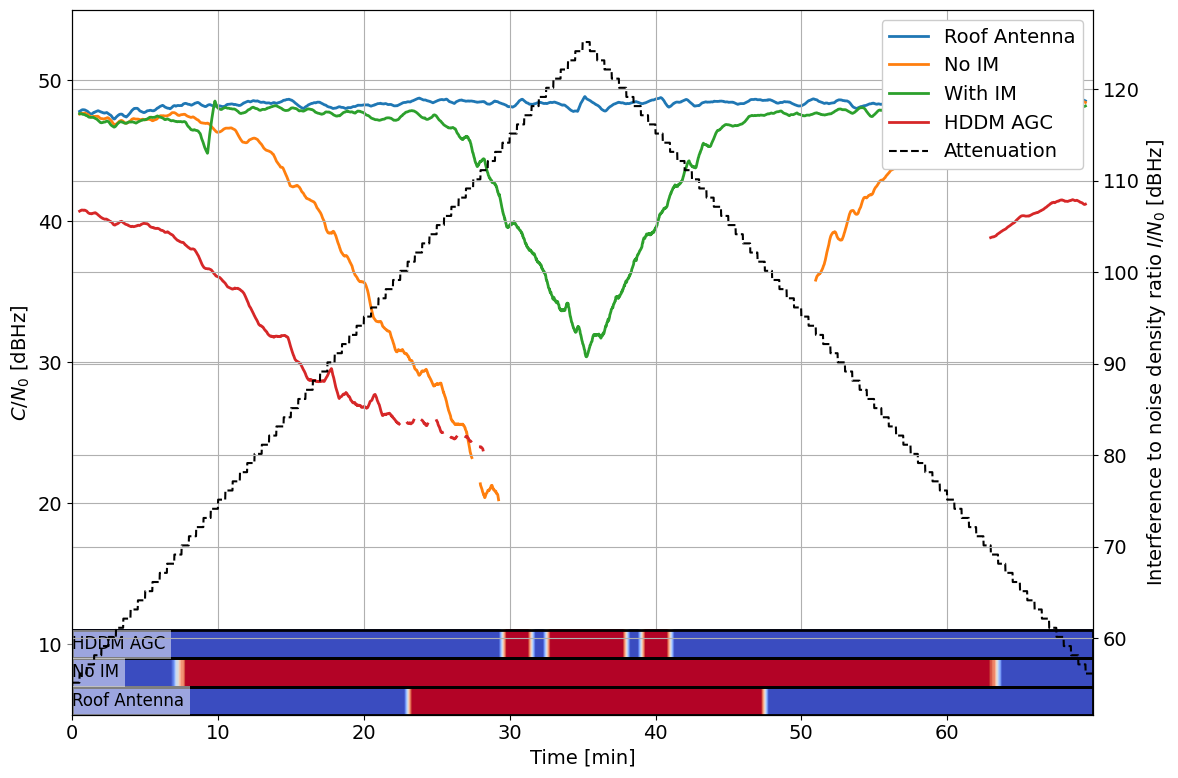

Supplement: Supplementary file 1 [file sensors-22-00679-s001.zip › results/Galileo/E1BC/15754200/HE_Bar_SVID7_CN0.png]

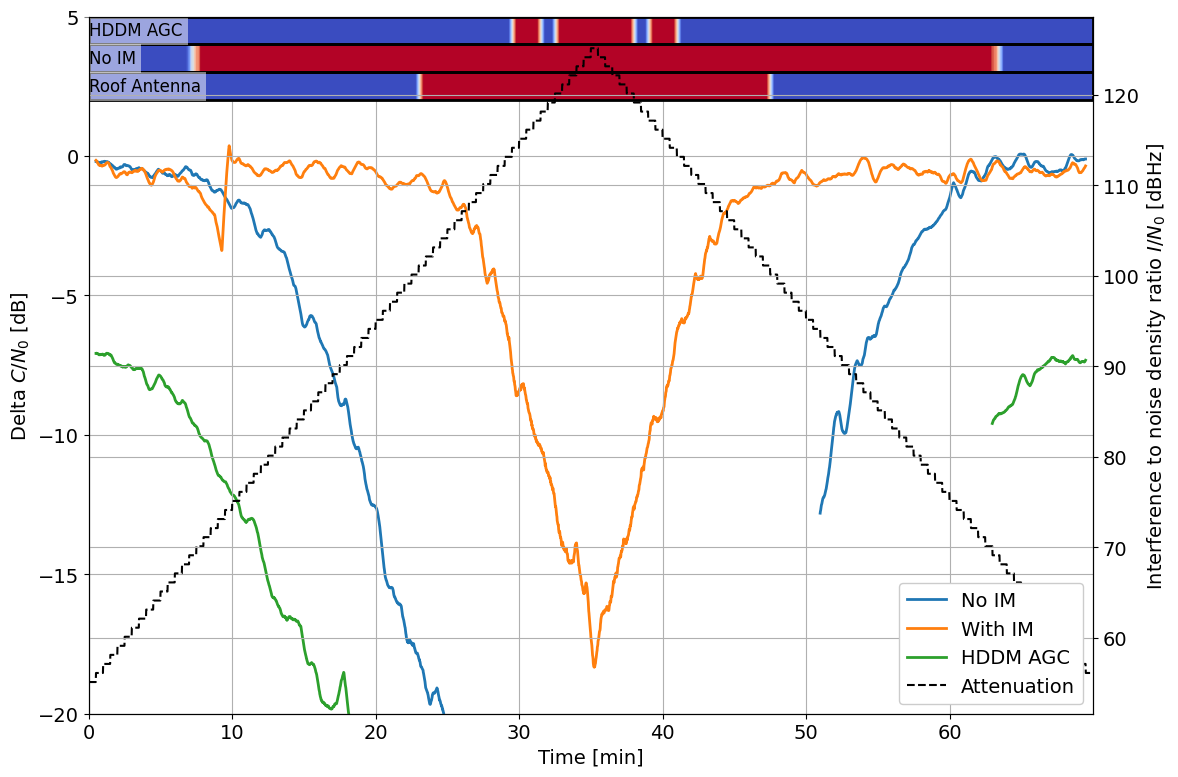

Supplement: Supplementary file 1 [file sensors-22-00679-s001.zip › results/Galileo/E1BC/15754200/HE_Bar_SVID7_DeltaCN0.png]

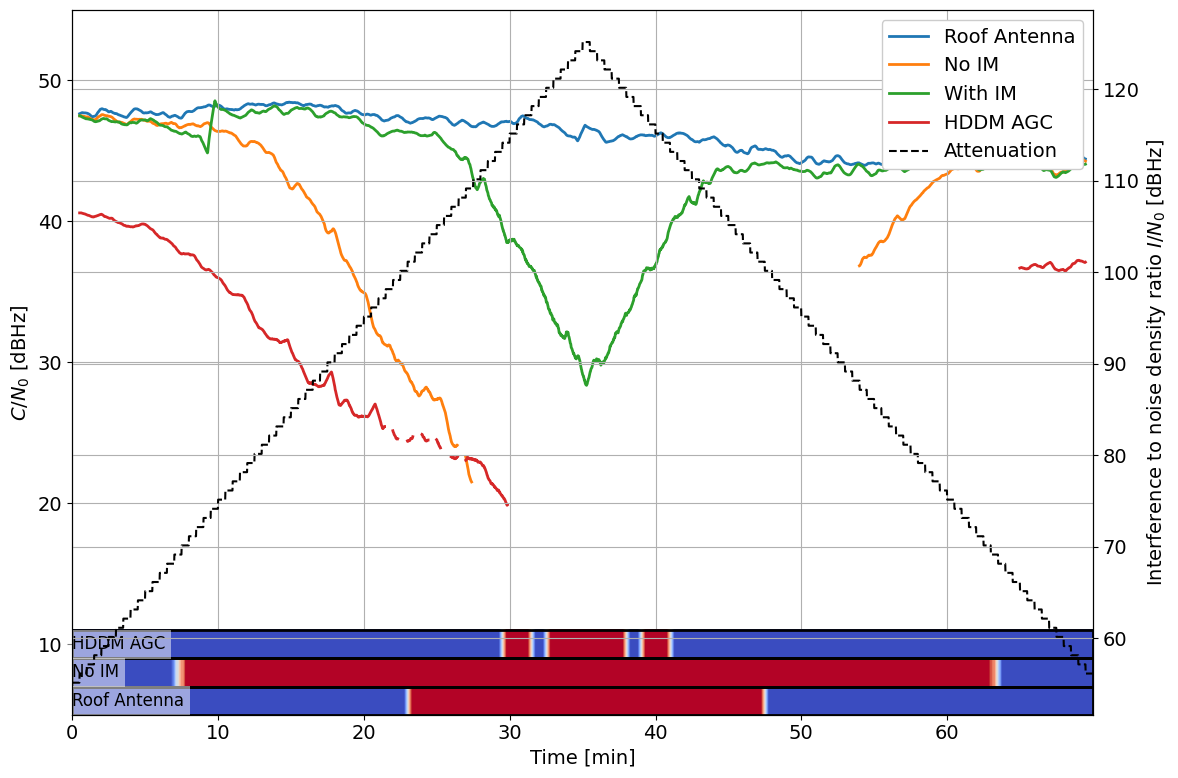

Supplement: Supplementary file 1 [file sensors-22-00679-s001.zip › results/Galileo/E1BC/15754200/HE_Bar_SVID8_CN0.png]

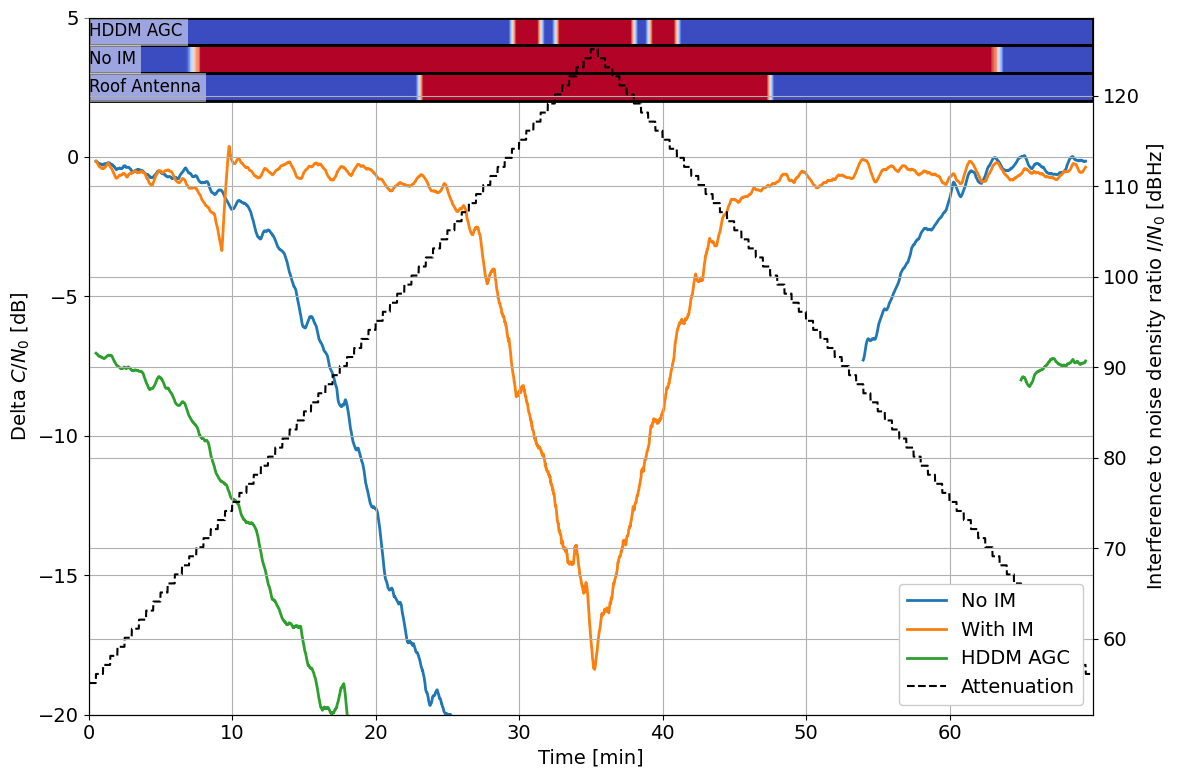

Supplement: Supplementary file 1 [file sensors-22-00679-s001.zip › results/Galileo/E1BC/15754200/HE_Bar_SVID8_DeltaCN0.png]

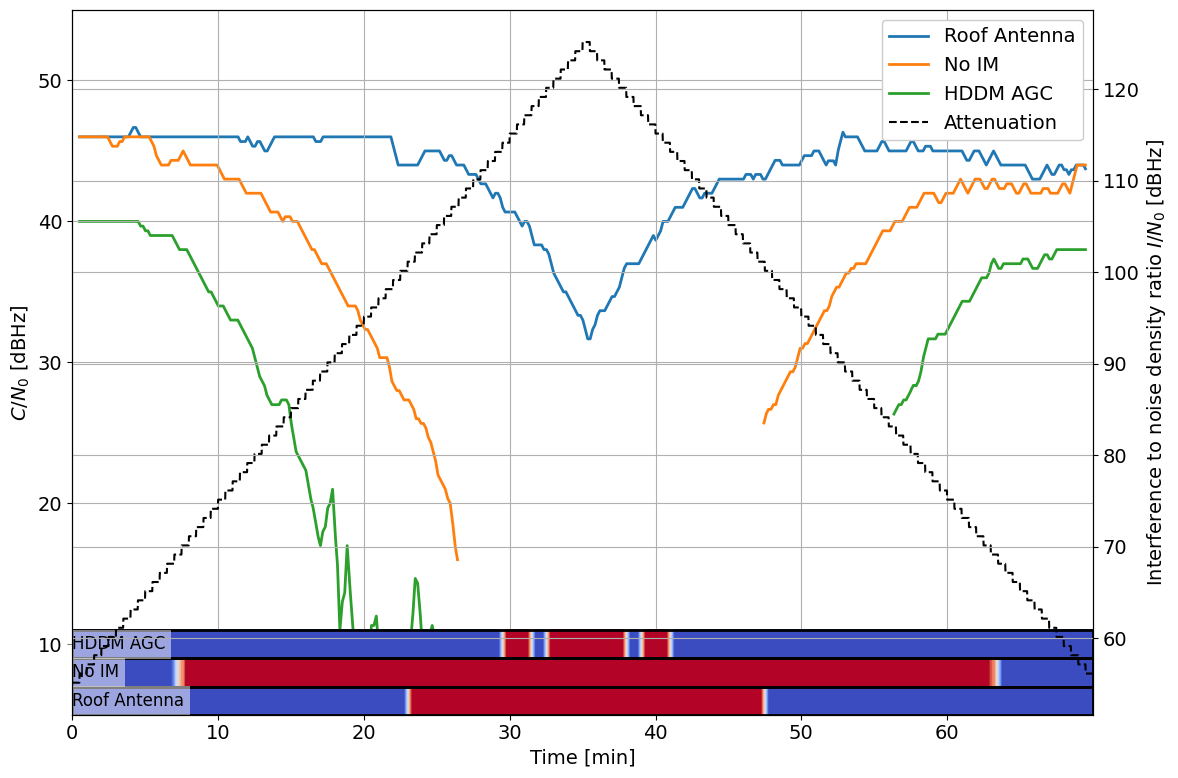

Supplement: Supplementary file 1 [file sensors-22-00679-s001.zip › results/Galileo/E1BC/15754200/LC_Bar_SVID2_CN0.png]

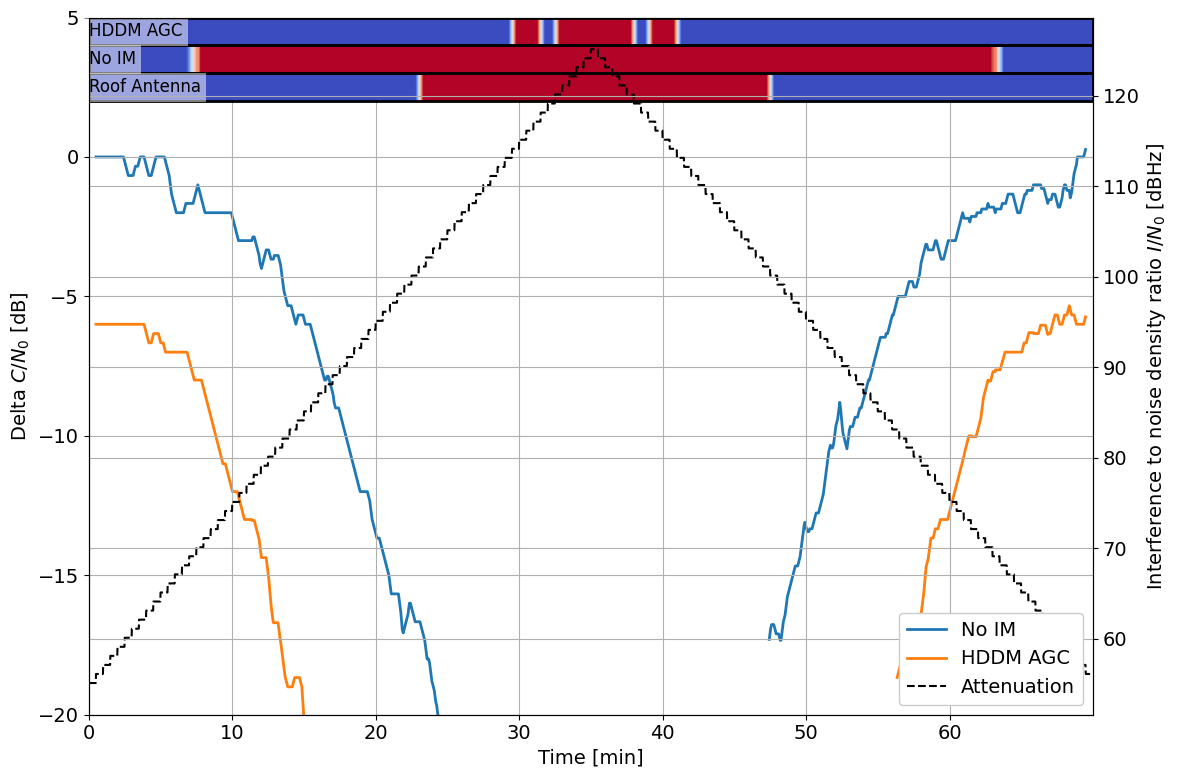

Supplement: Supplementary file 1 [file sensors-22-00679-s001.zip › results/Galileo/E1BC/15754200/LC_Bar_SVID2_DeltaCN0.png]

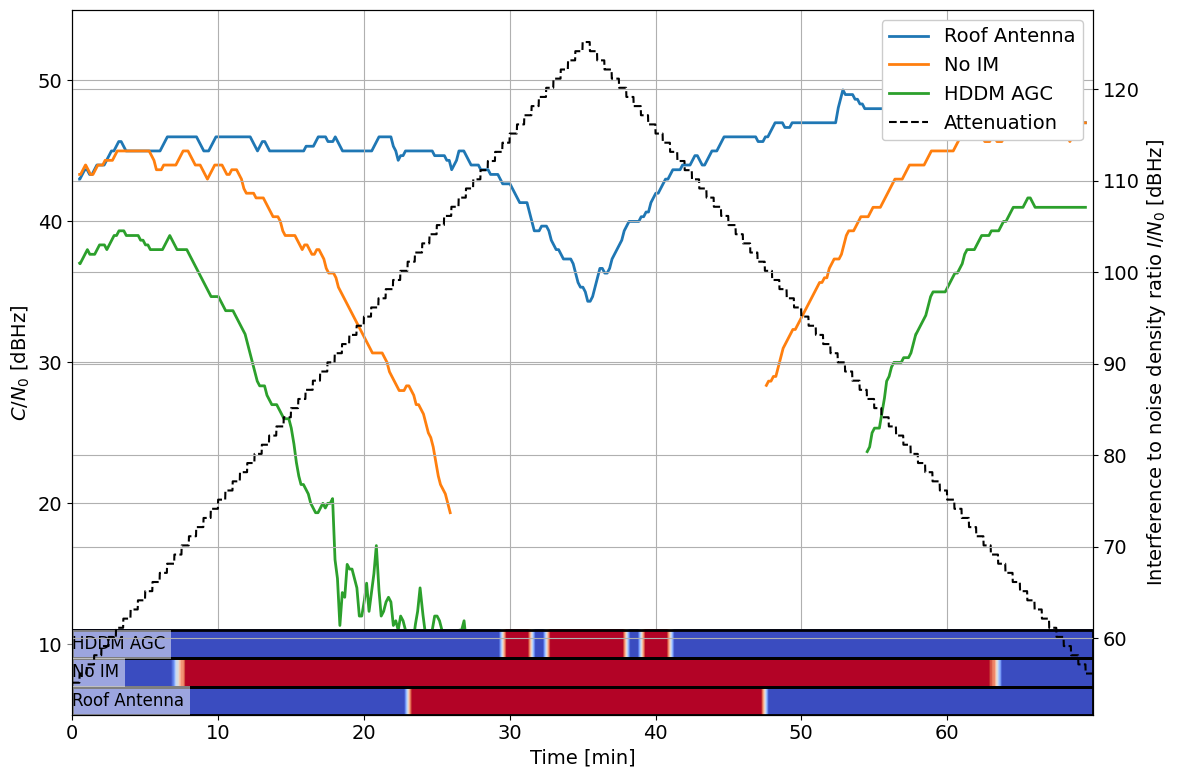

Supplement: Supplementary file 1 [file sensors-22-00679-s001.zip › results/Galileo/E1BC/15754200/LC_Bar_SVID30_CN0.png]

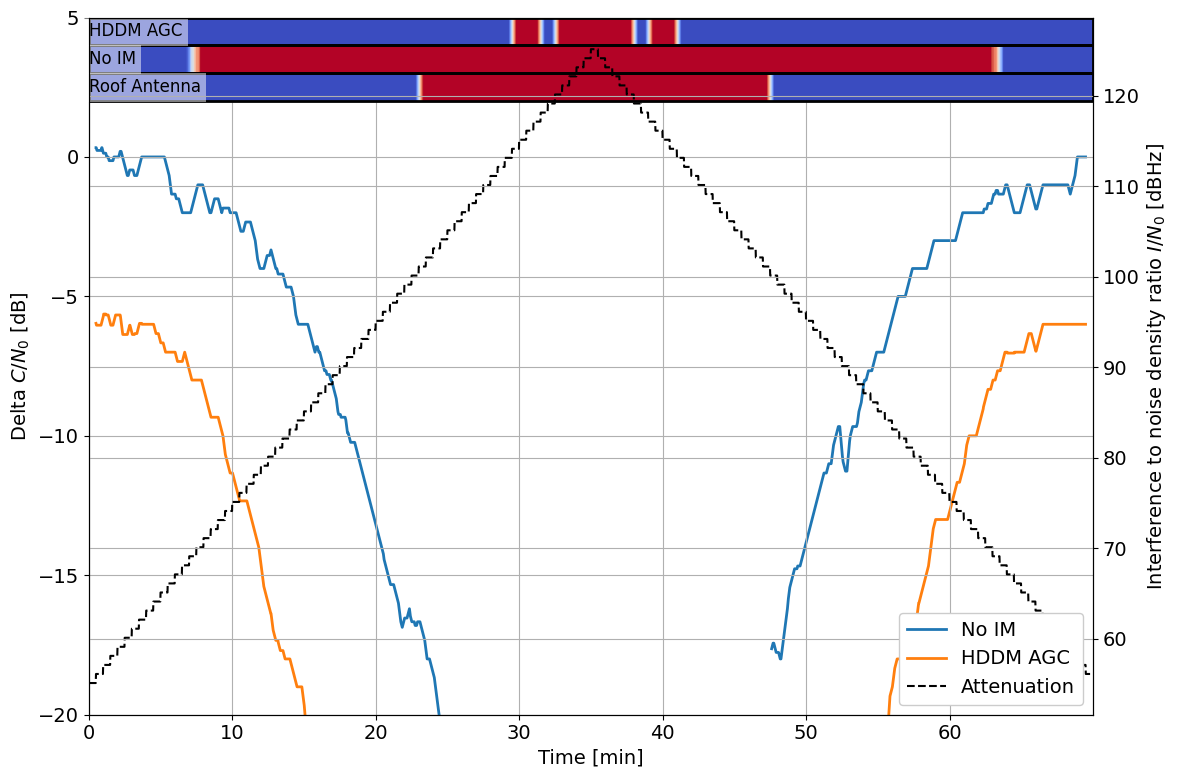

Supplement: Supplementary file 1 [file sensors-22-00679-s001.zip › results/Galileo/E1BC/15754200/LC_Bar_SVID30_DeltaCN0.png]

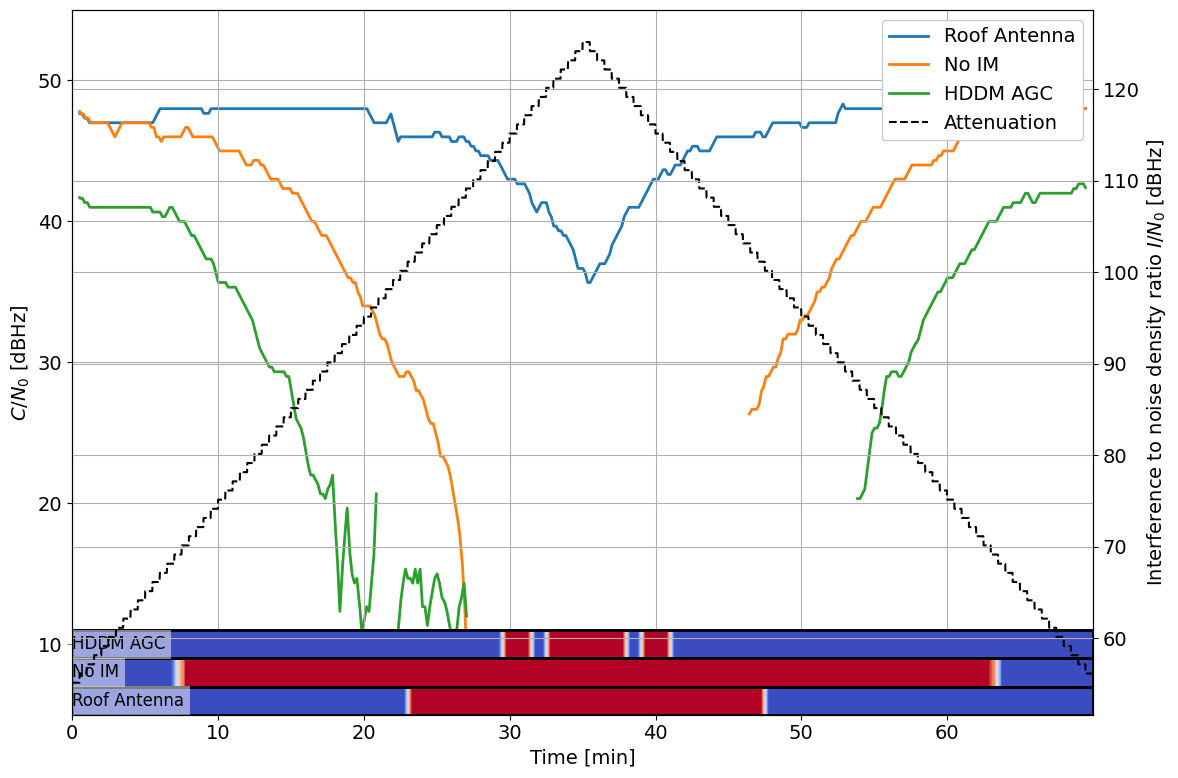

Supplement: Supplementary file 1 [file sensors-22-00679-s001.zip › results/Galileo/E1BC/15754200/LC_Bar_SVID7_CN0.png]

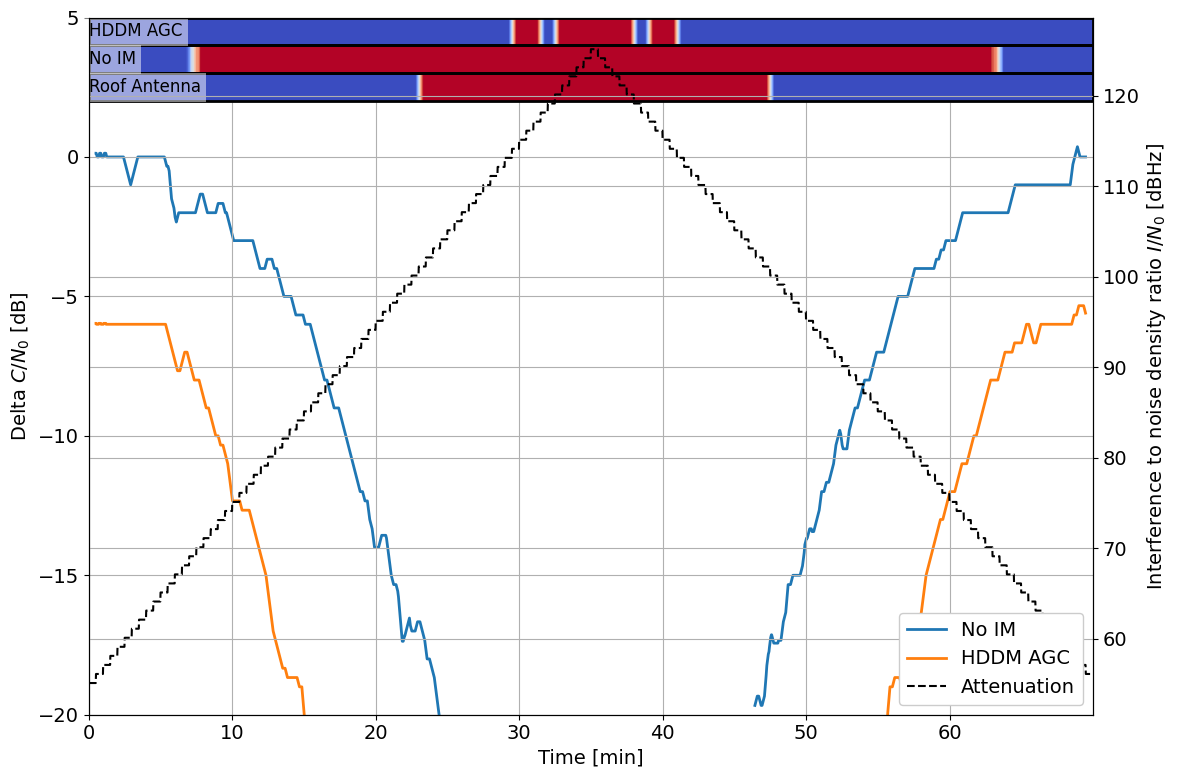

Supplement: Supplementary file 1 [file sensors-22-00679-s001.zip › results/Galileo/E1BC/15754200/LC_Bar_SVID7_DeltaCN0.png]

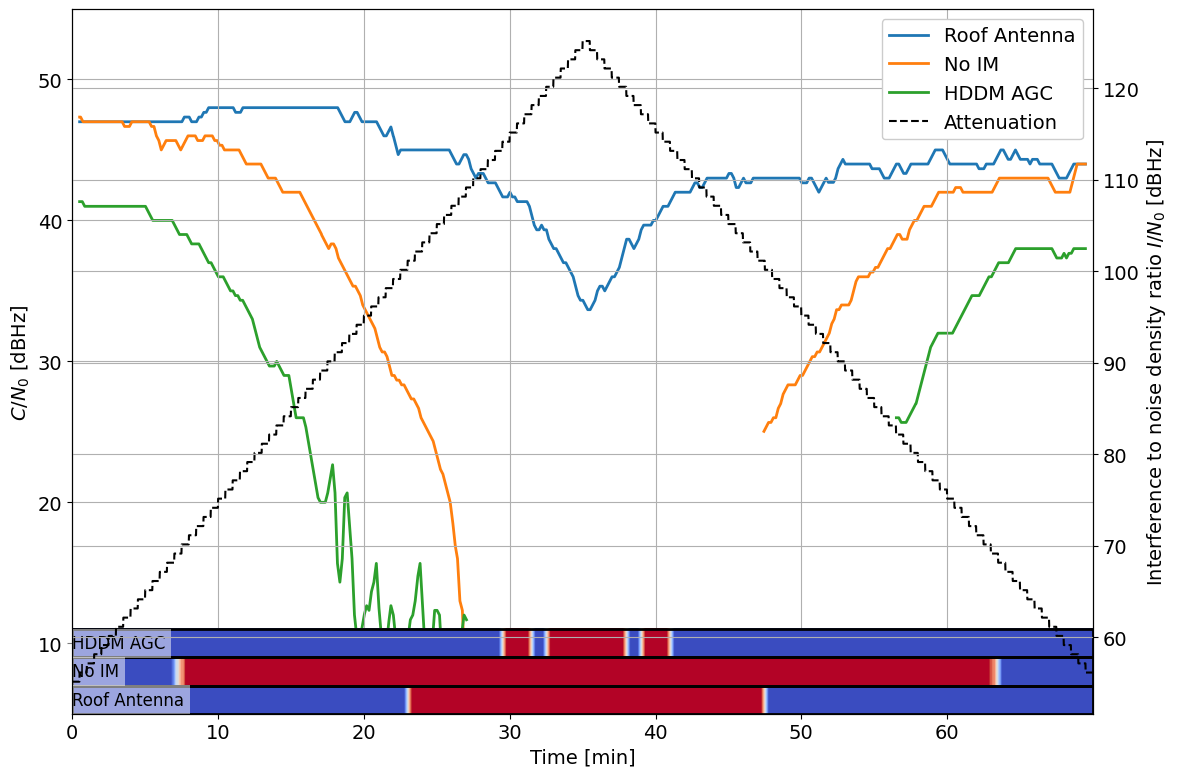

Supplement: Supplementary file 1 [file sensors-22-00679-s001.zip › results/Galileo/E1BC/15754200/LC_Bar_SVID8_CN0.png]

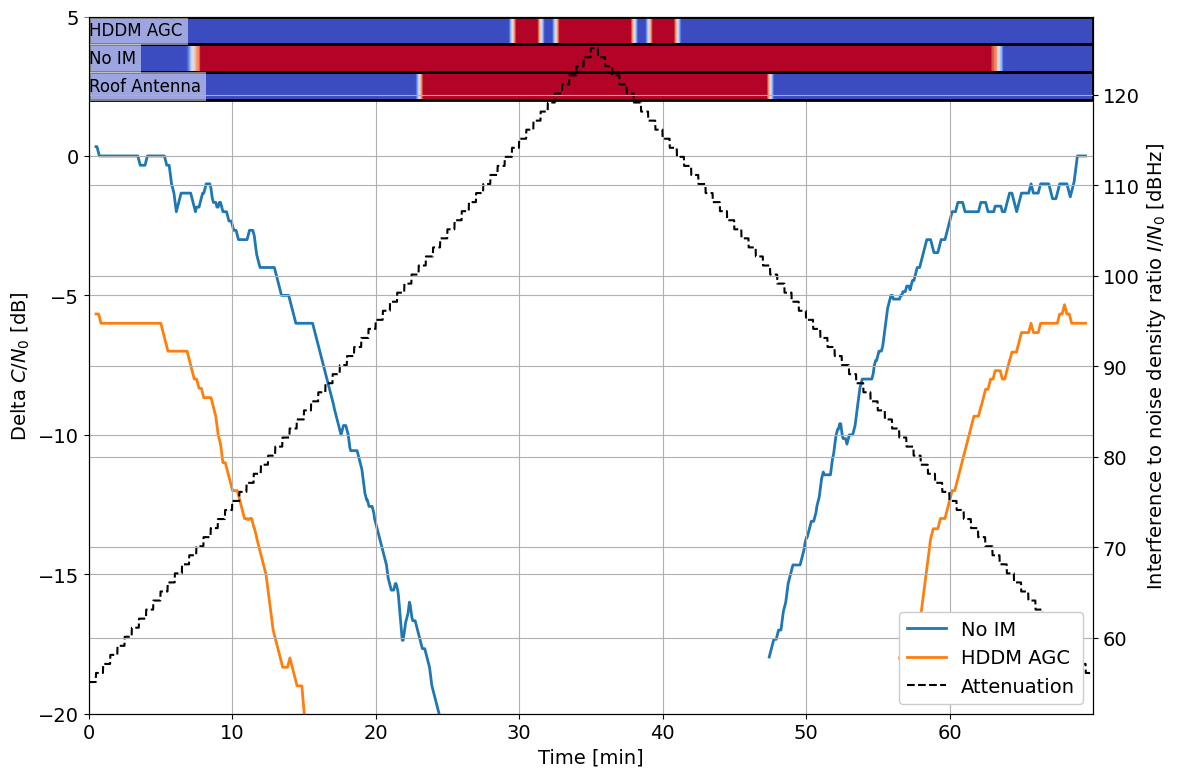

Supplement: Supplementary file 1 [file sensors-22-00679-s001.zip › results/Galileo/E1BC/15754200/LC_Bar_SVID8_DeltaCN0.png]

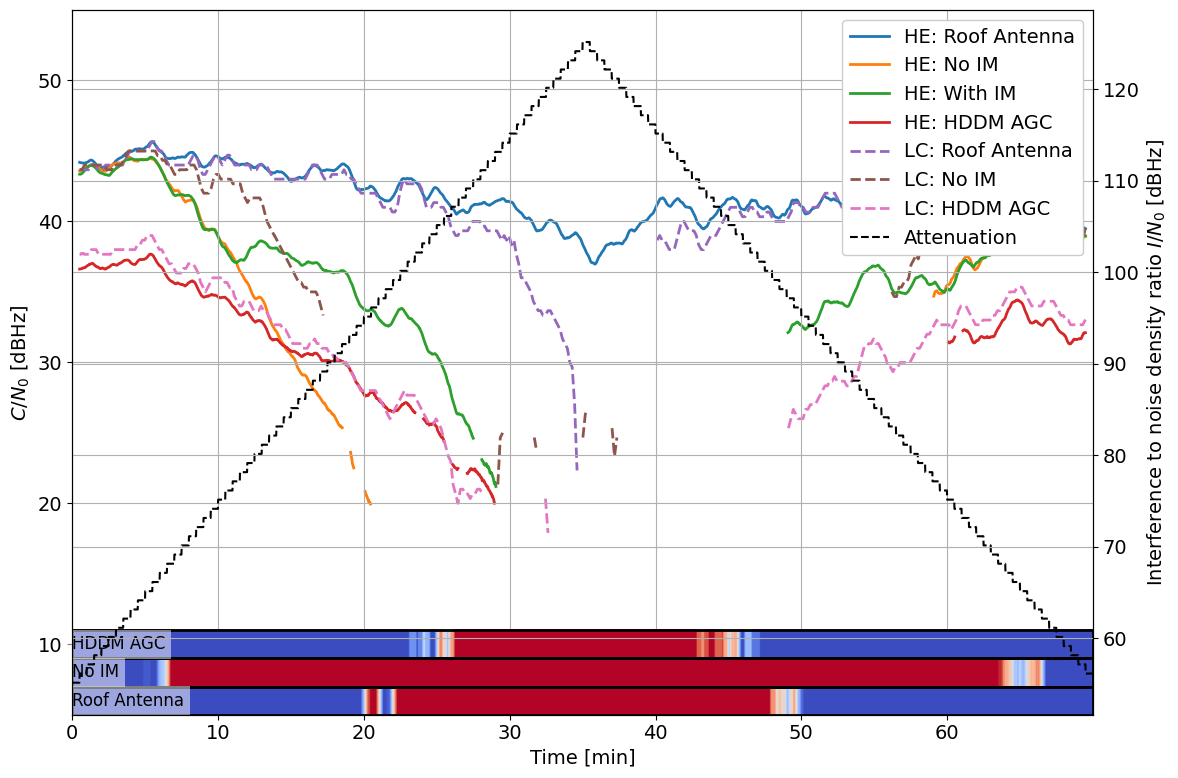

Supplement: Supplementary file 1 [file sensors-22-00679-s001.zip › results/Galileo/E1BC/Chirp_LinearFast_BW10/HELC_Bar_SVID13_CN0.png]

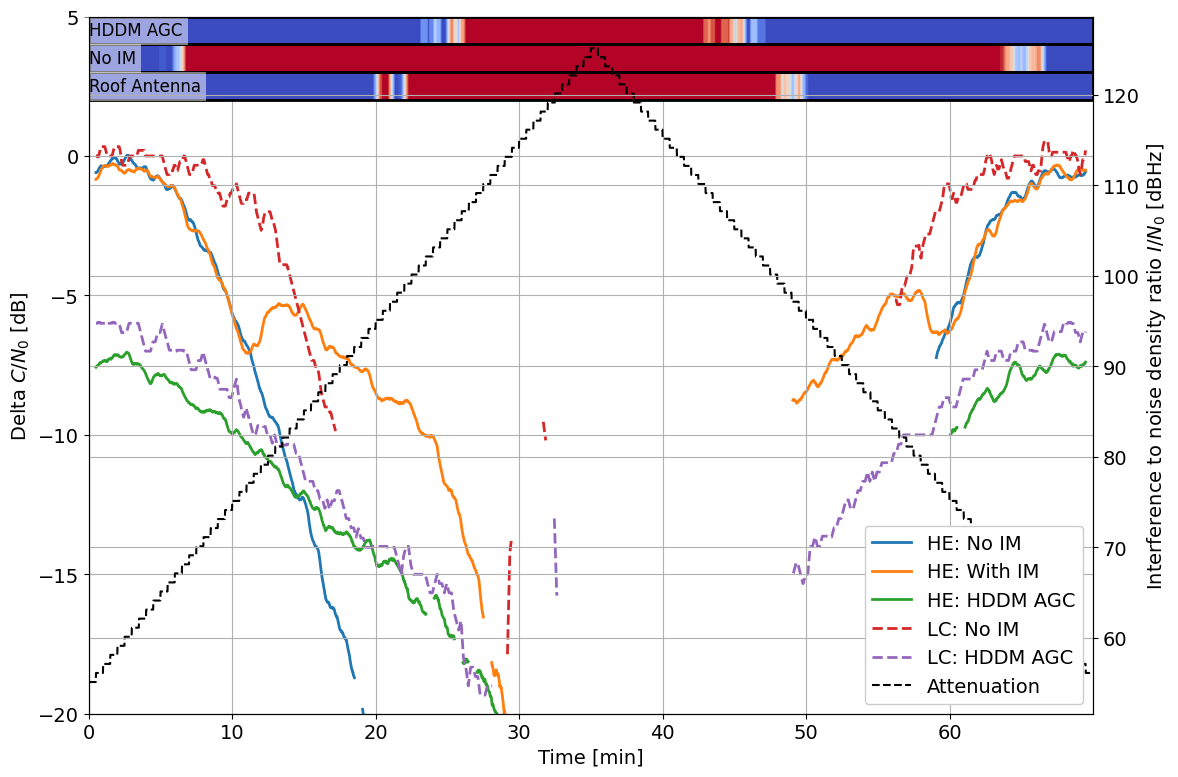

Supplement: Supplementary file 1 [file sensors-22-00679-s001.zip › results/Galileo/E1BC/Chirp_LinearFast_BW10/HELC_Bar_SVID13_DeltaCN0.png]

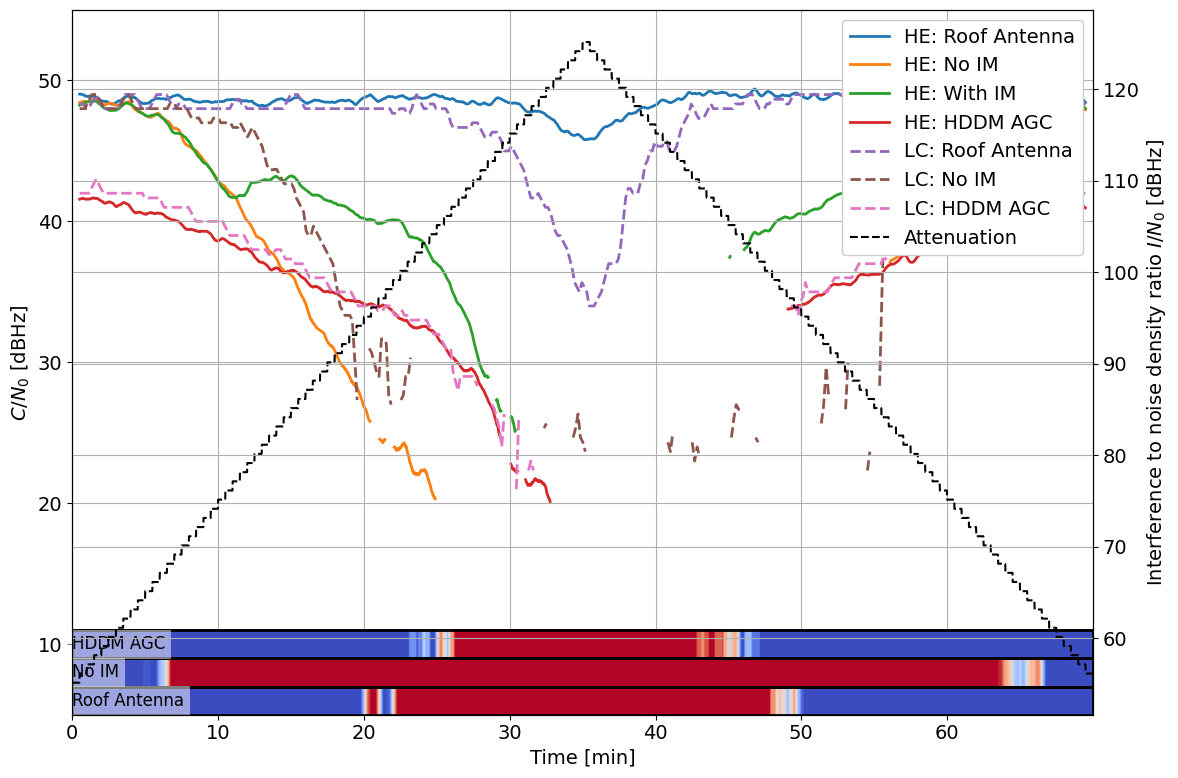

Supplement: Supplementary file 1 [file sensors-22-00679-s001.zip › results/Galileo/E1BC/Chirp_LinearFast_BW10/HELC_Bar_SVID1_CN0.png]

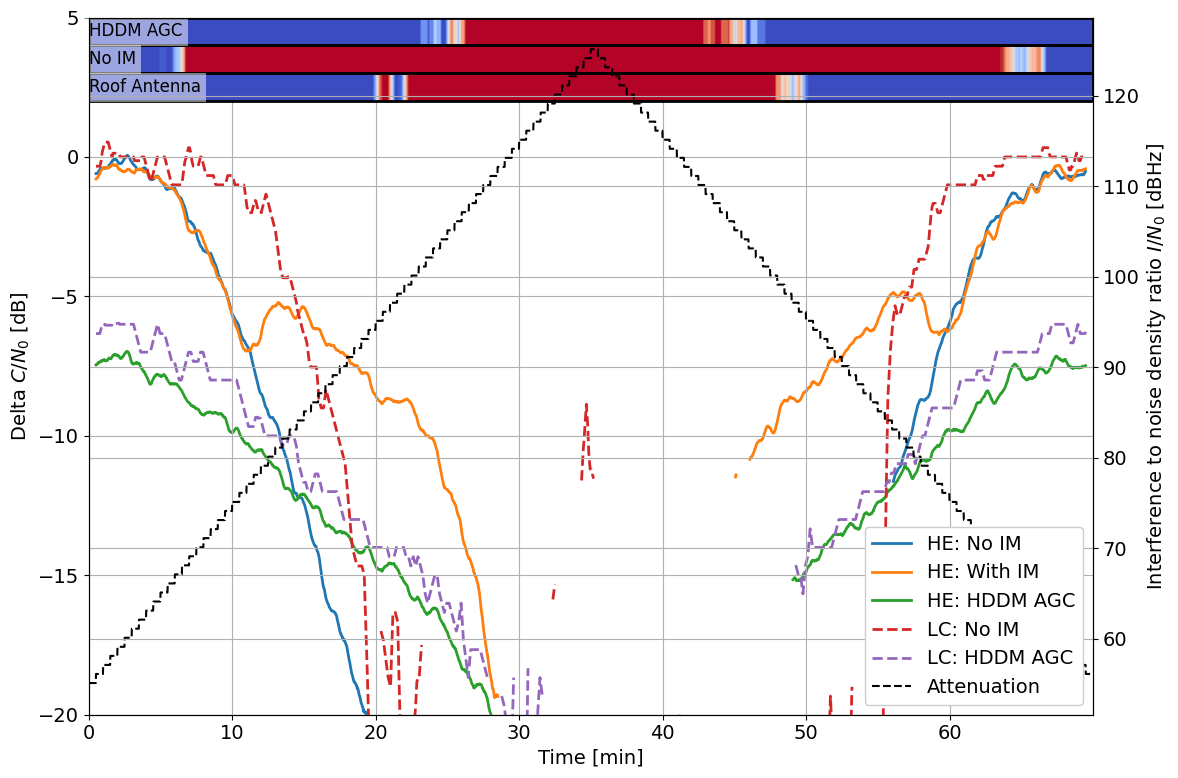

Supplement: Supplementary file 1 [file sensors-22-00679-s001.zip › results/Galileo/E1BC/Chirp_LinearFast_BW10/HELC_Bar_SVID1_DeltaCN0.png]

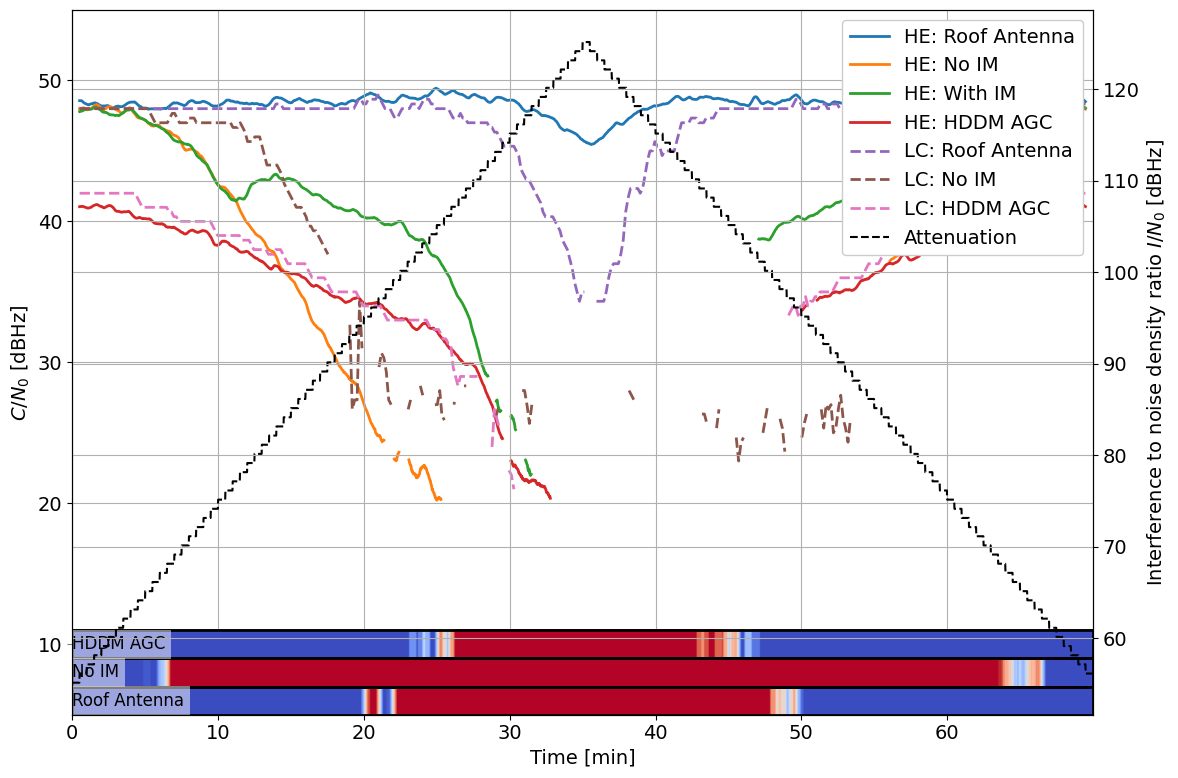

Supplement: Supplementary file 1 [file sensors-22-00679-s001.zip › results/Galileo/E1BC/Chirp_LinearFast_BW10/HELC_Bar_SVID26_CN0.png]

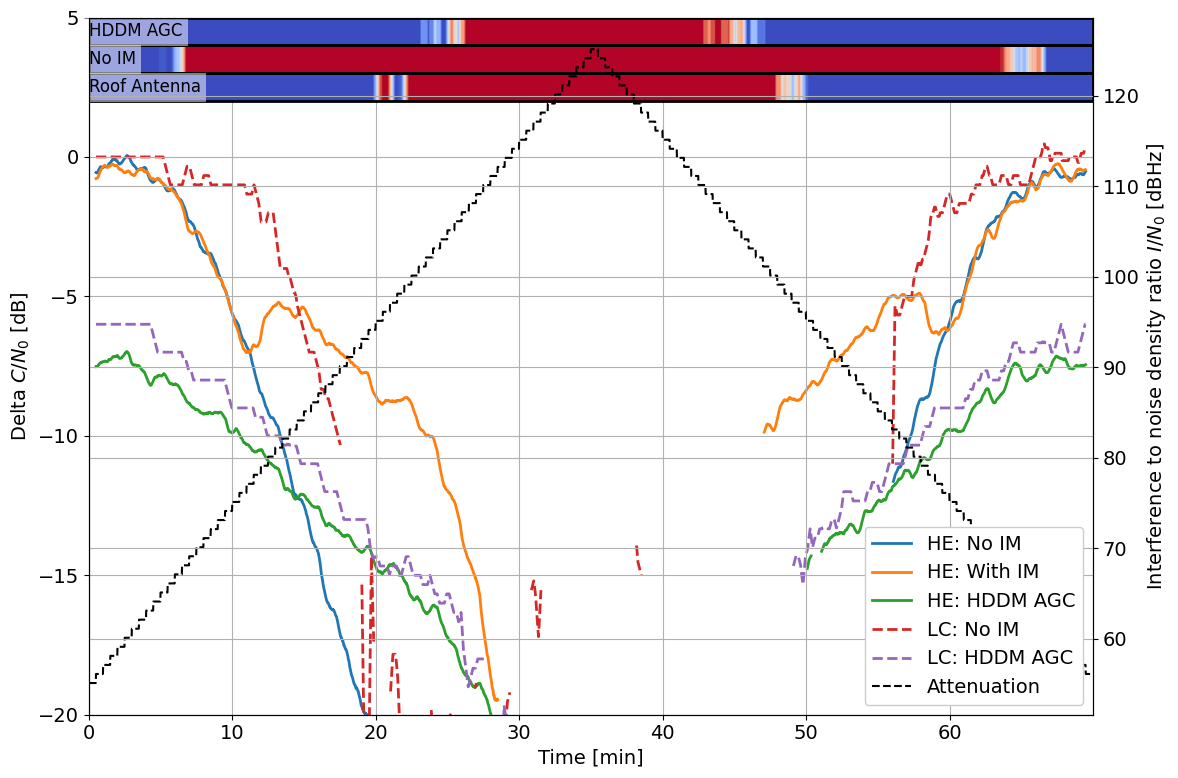

Supplement: Supplementary file 1 [file sensors-22-00679-s001.zip › results/Galileo/E1BC/Chirp_LinearFast_BW10/HELC_Bar_SVID26_DeltaCN0.png]

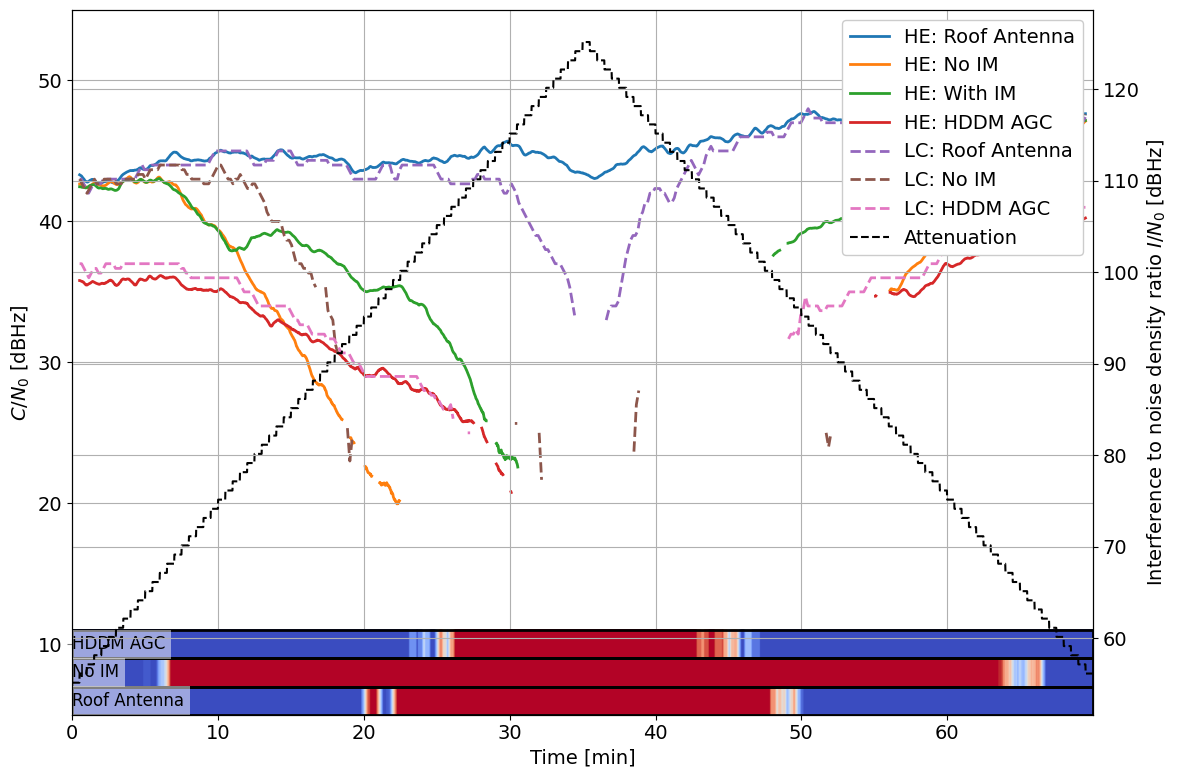

Supplement: Supplementary file 1 [file sensors-22-00679-s001.zip › results/Galileo/E1BC/Chirp_LinearFast_BW10/HELC_Bar_SVID31_CN0.png]

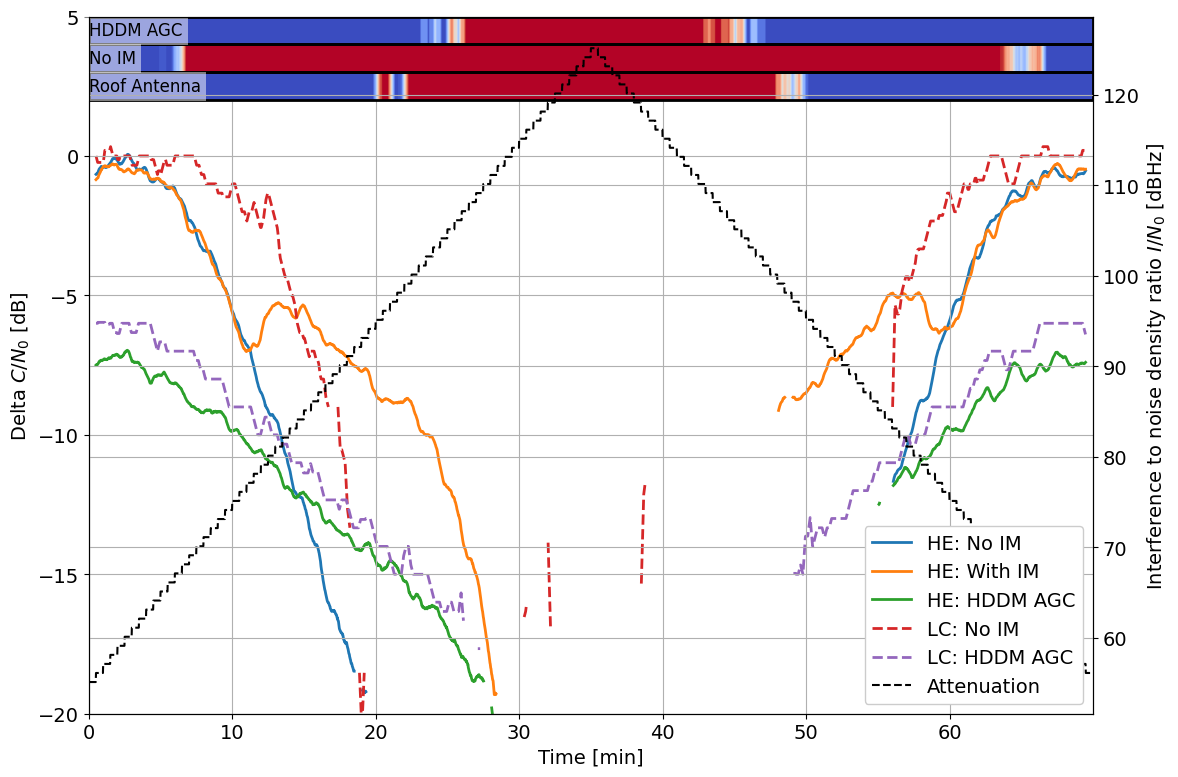

Supplement: Supplementary file 1 [file sensors-22-00679-s001.zip › results/Galileo/E1BC/Chirp_LinearFast_BW10/HELC_Bar_SVID31_DeltaCN0.png]

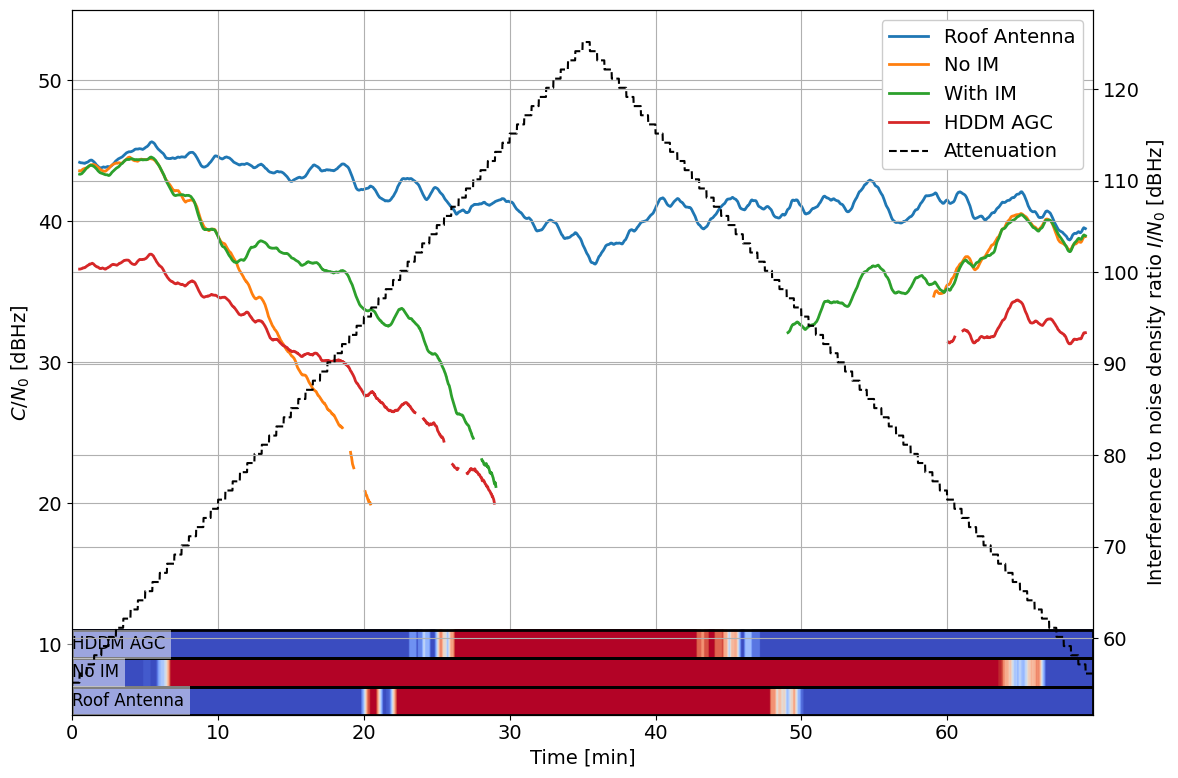

Supplement: Supplementary file 1 [file sensors-22-00679-s001.zip › results/Galileo/E1BC/Chirp_LinearFast_BW10/HE_Bar_SVID13_CN0.png]

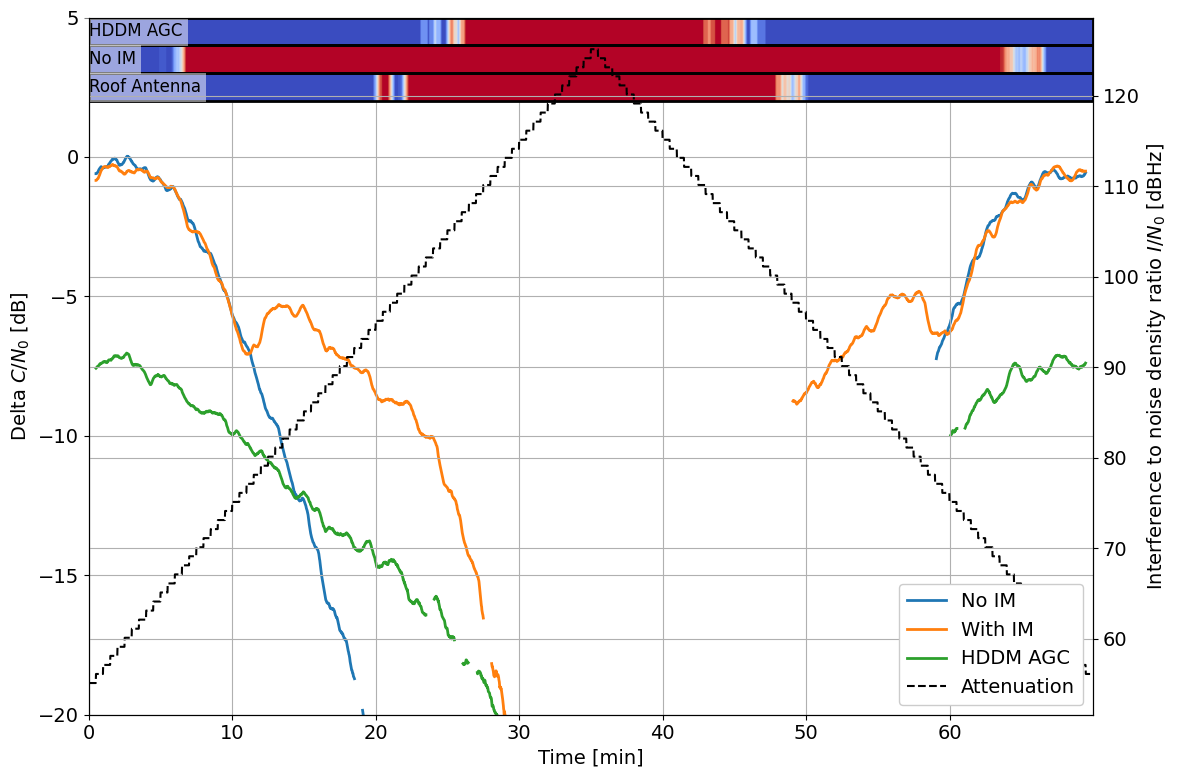

Supplement: Supplementary file 1 [file sensors-22-00679-s001.zip › results/Galileo/E1BC/Chirp_LinearFast_BW10/HE_Bar_SVID13_DeltaCN0.png]

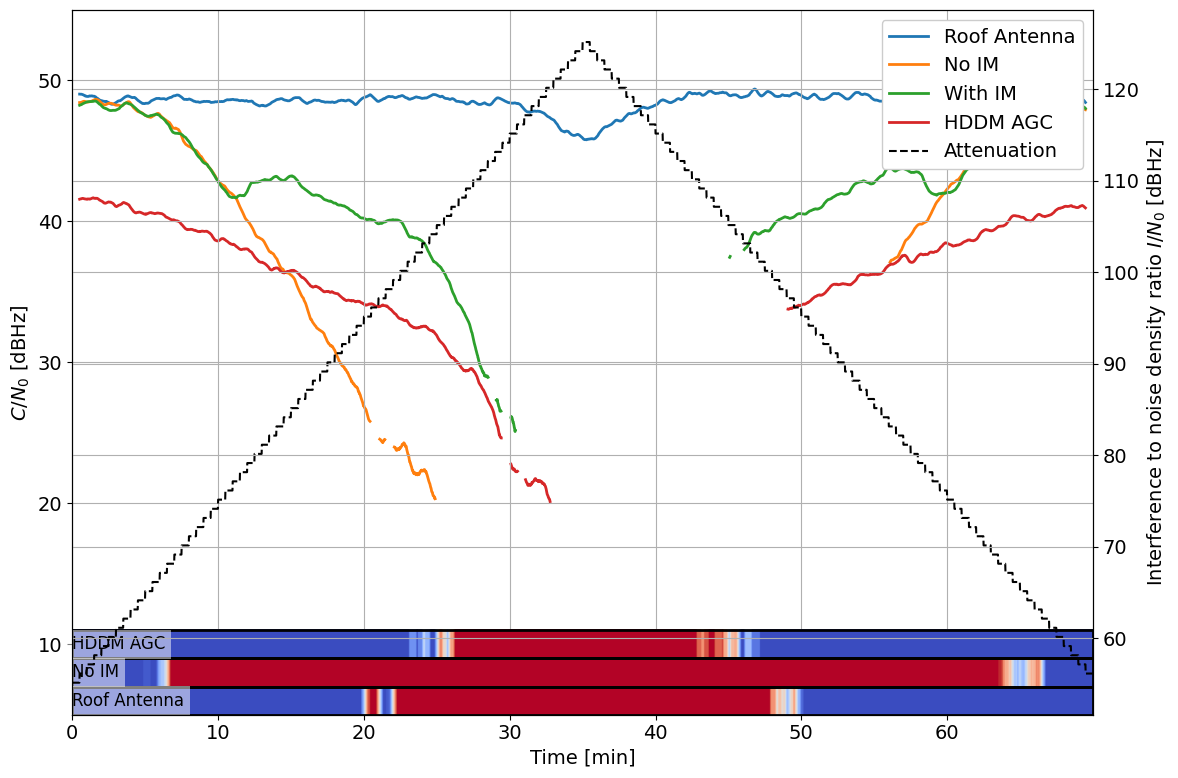

Supplement: Supplementary file 1 [file sensors-22-00679-s001.zip › results/Galileo/E1BC/Chirp_LinearFast_BW10/HE_Bar_SVID1_CN0.png]

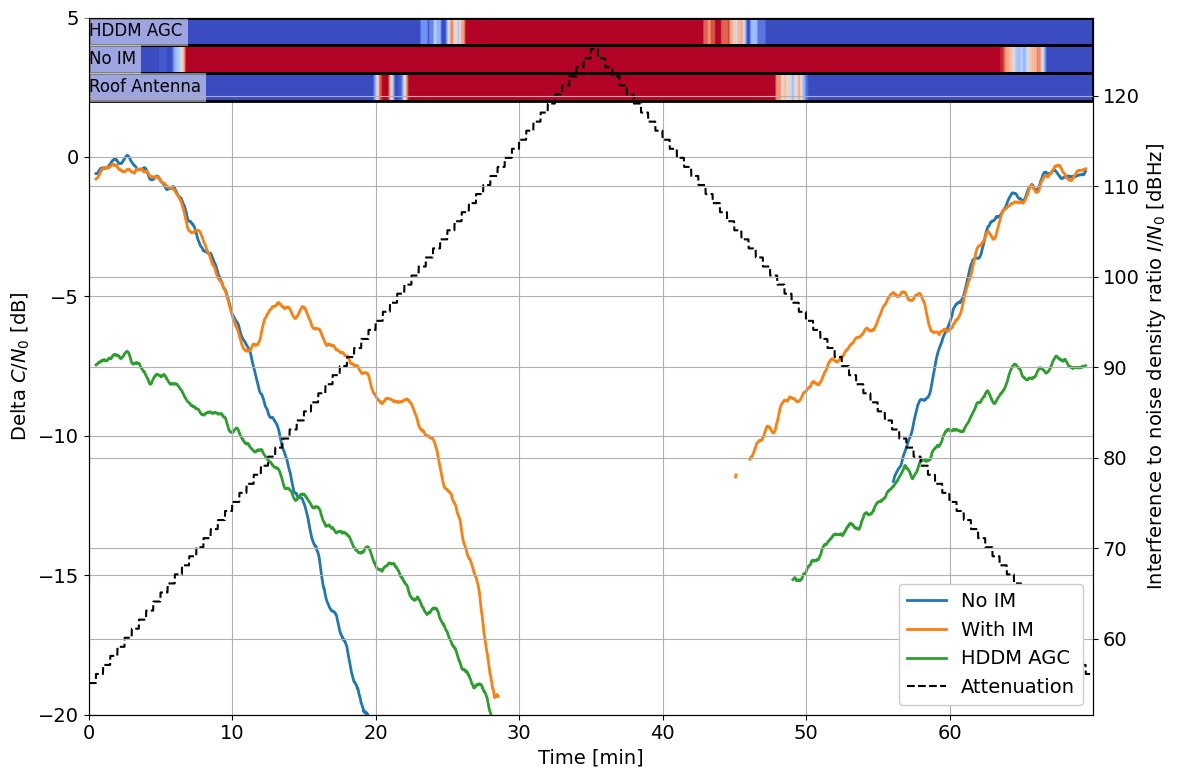

Supplement: Supplementary file 1 [file sensors-22-00679-s001.zip › results/Galileo/E1BC/Chirp_LinearFast_BW10/HE_Bar_SVID1_DeltaCN0.png]

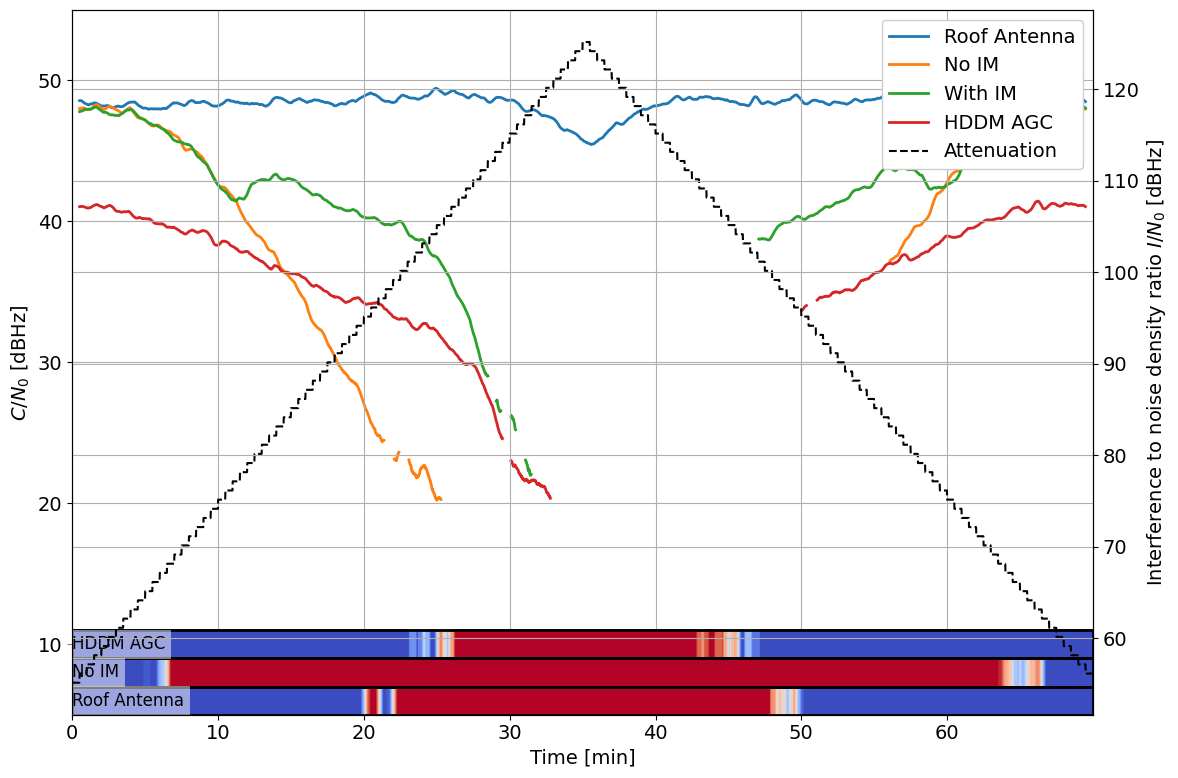

Supplement: Supplementary file 1 [file sensors-22-00679-s001.zip › results/Galileo/E1BC/Chirp_LinearFast_BW10/HE_Bar_SVID26_CN0.png]

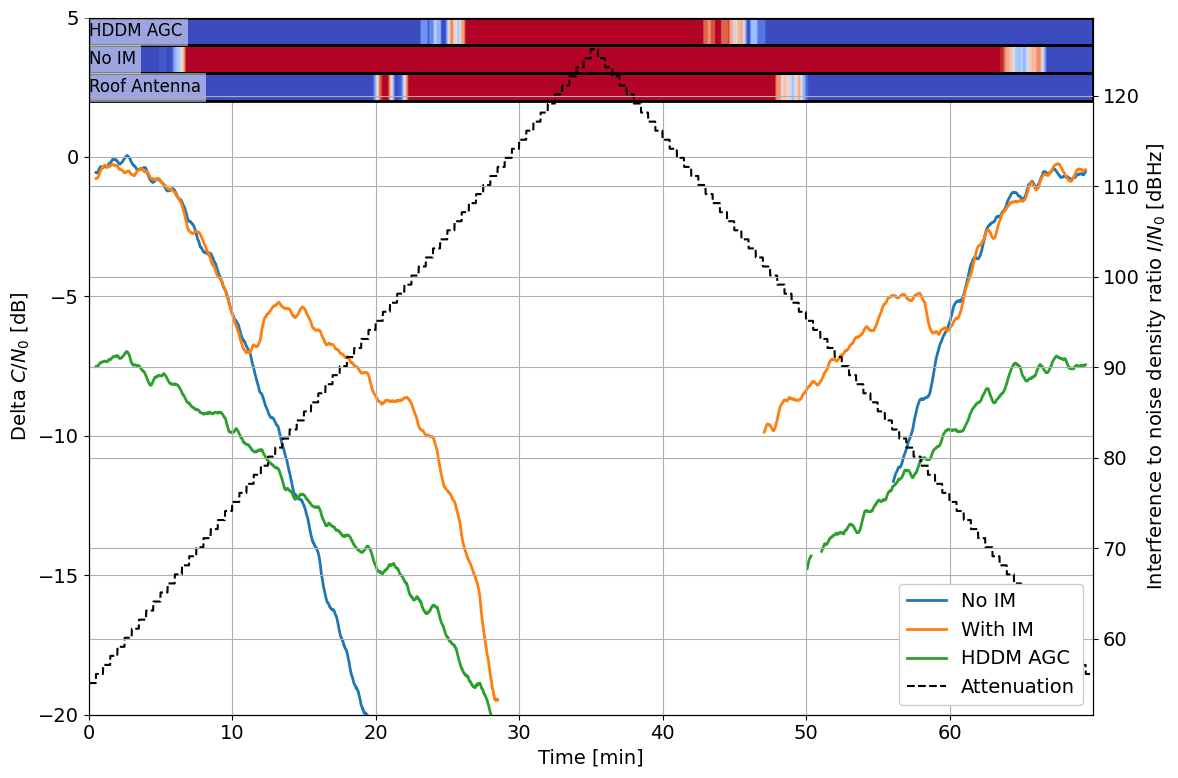

Supplement: Supplementary file 1 [file sensors-22-00679-s001.zip › results/Galileo/E1BC/Chirp_LinearFast_BW10/HE_Bar_SVID26_DeltaCN0.png]

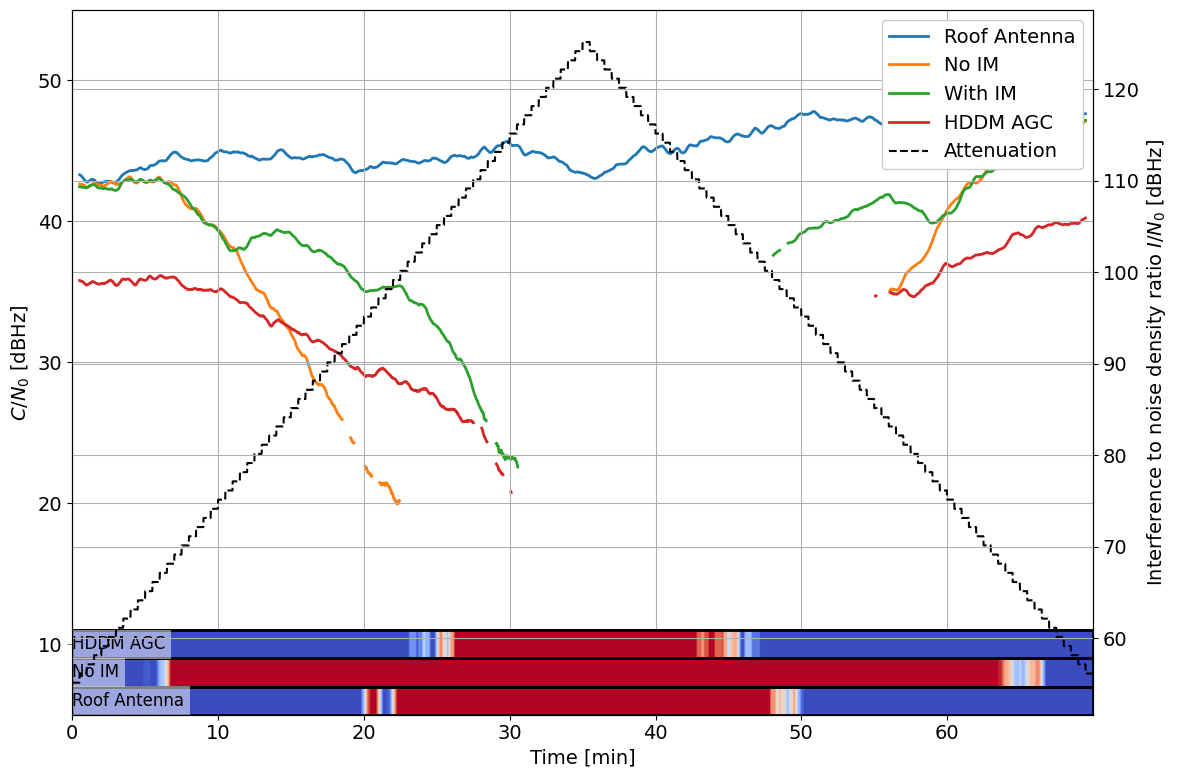

Supplement: Supplementary file 1 [file sensors-22-00679-s001.zip › results/Galileo/E1BC/Chirp_LinearFast_BW10/HE_Bar_SVID31_CN0.png]

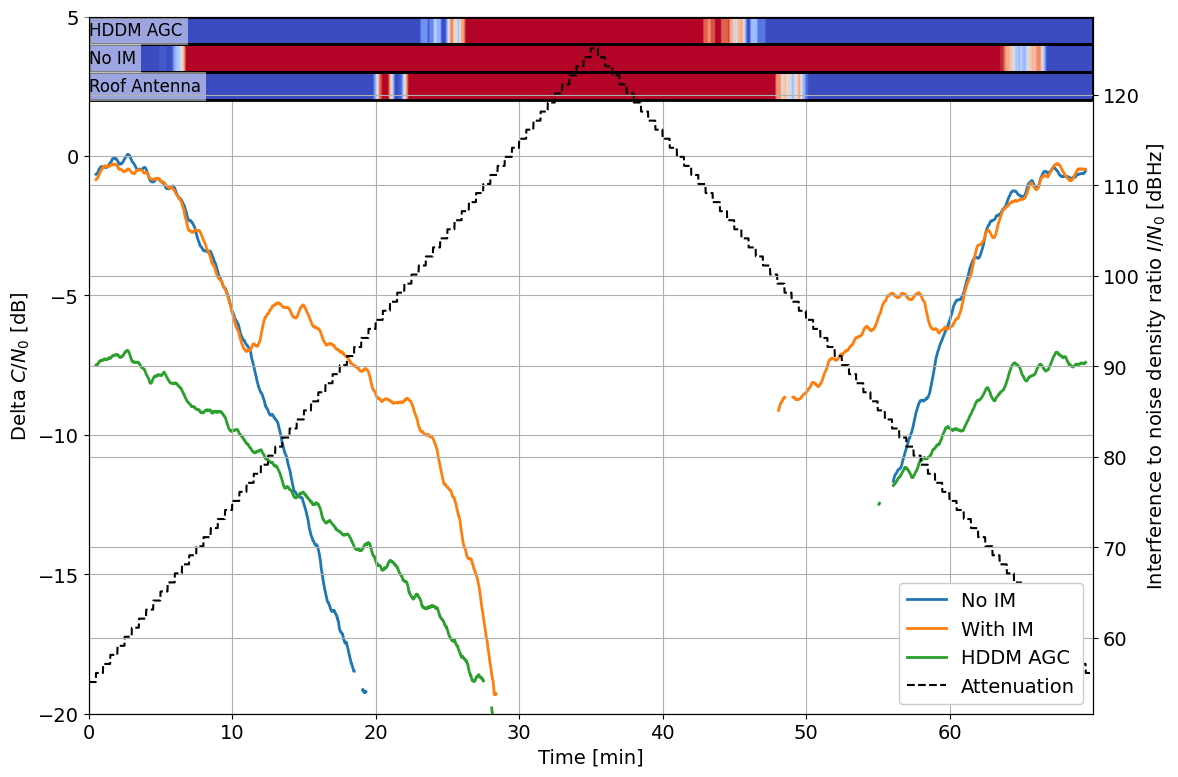

Supplement: Supplementary file 1 [file sensors-22-00679-s001.zip › results/Galileo/E1BC/Chirp_LinearFast_BW10/HE_Bar_SVID31_DeltaCN0.png]

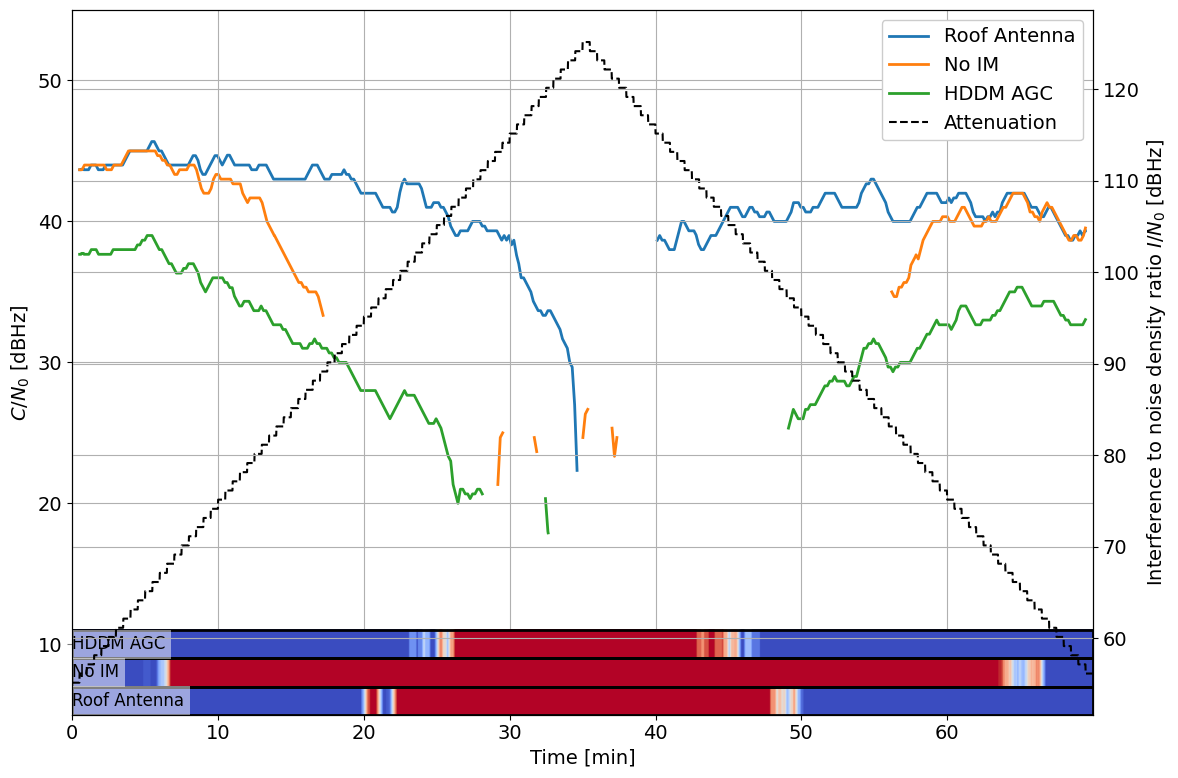

Supplement: Supplementary file 1 [file sensors-22-00679-s001.zip › results/Galileo/E1BC/Chirp_LinearFast_BW10/LC_Bar_SVID13_CN0.png]

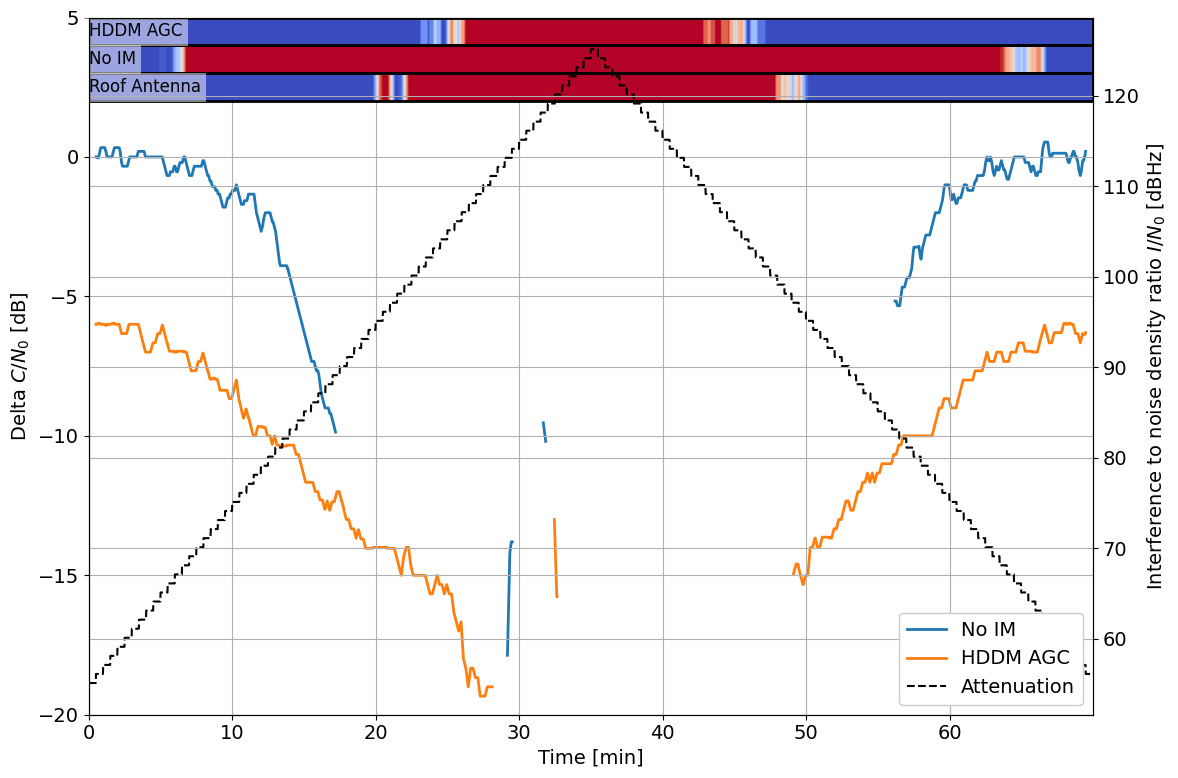

Supplement: Supplementary file 1 [file sensors-22-00679-s001.zip › results/Galileo/E1BC/Chirp_LinearFast_BW10/LC_Bar_SVID13_DeltaCN0.png]

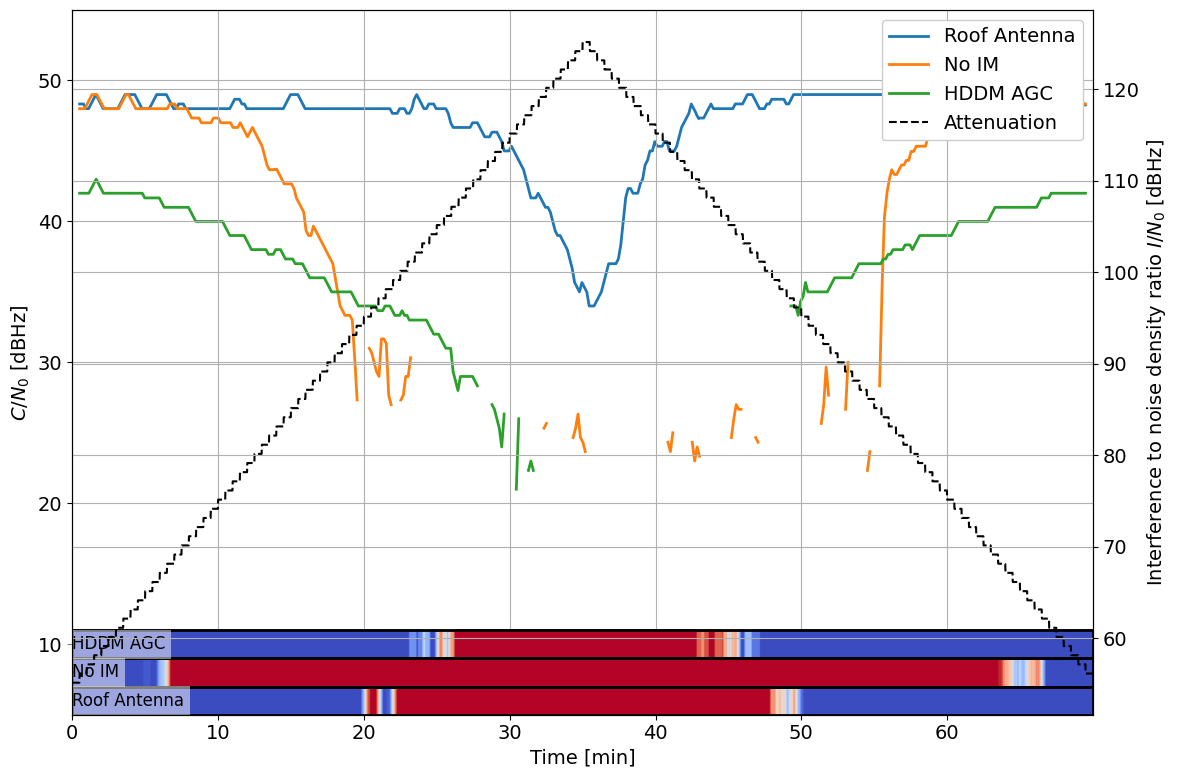

Supplement: Supplementary file 1 [file sensors-22-00679-s001.zip › results/Galileo/E1BC/Chirp_LinearFast_BW10/LC_Bar_SVID1_CN0.png]

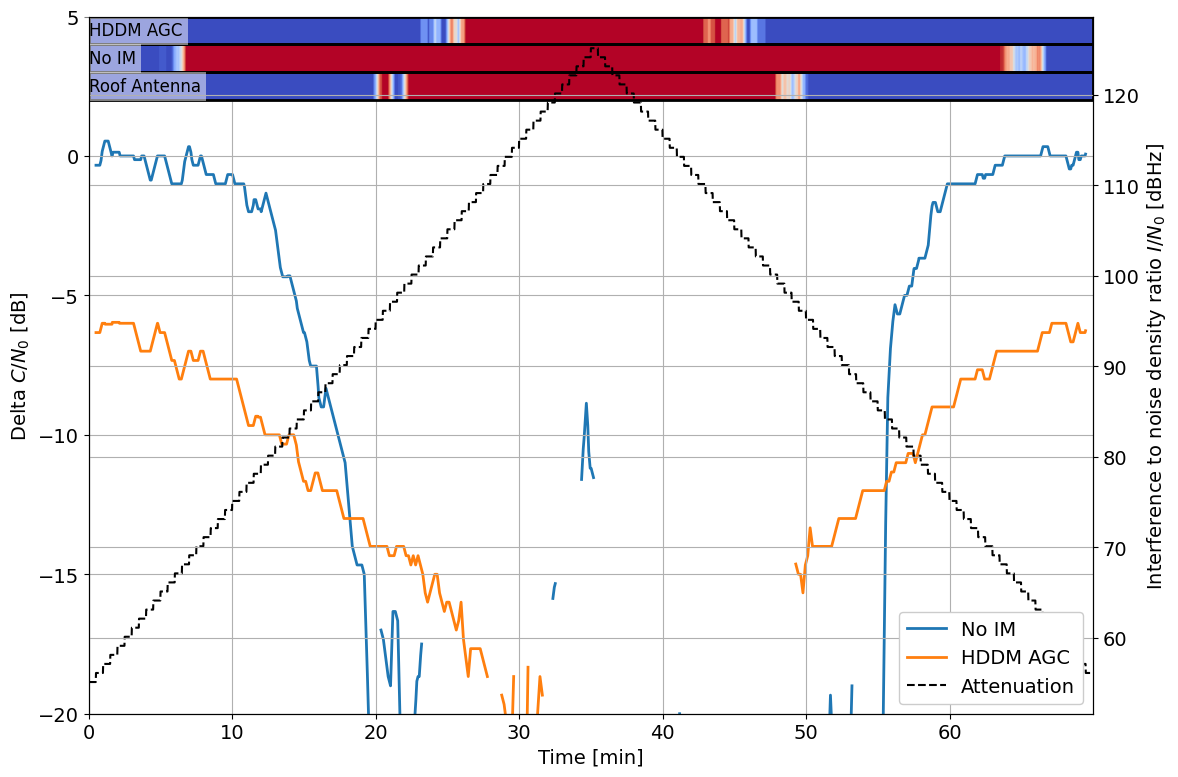

Supplement: Supplementary file 1 [file sensors-22-00679-s001.zip › results/Galileo/E1BC/Chirp_LinearFast_BW10/LC_Bar_SVID1_DeltaCN0.png]

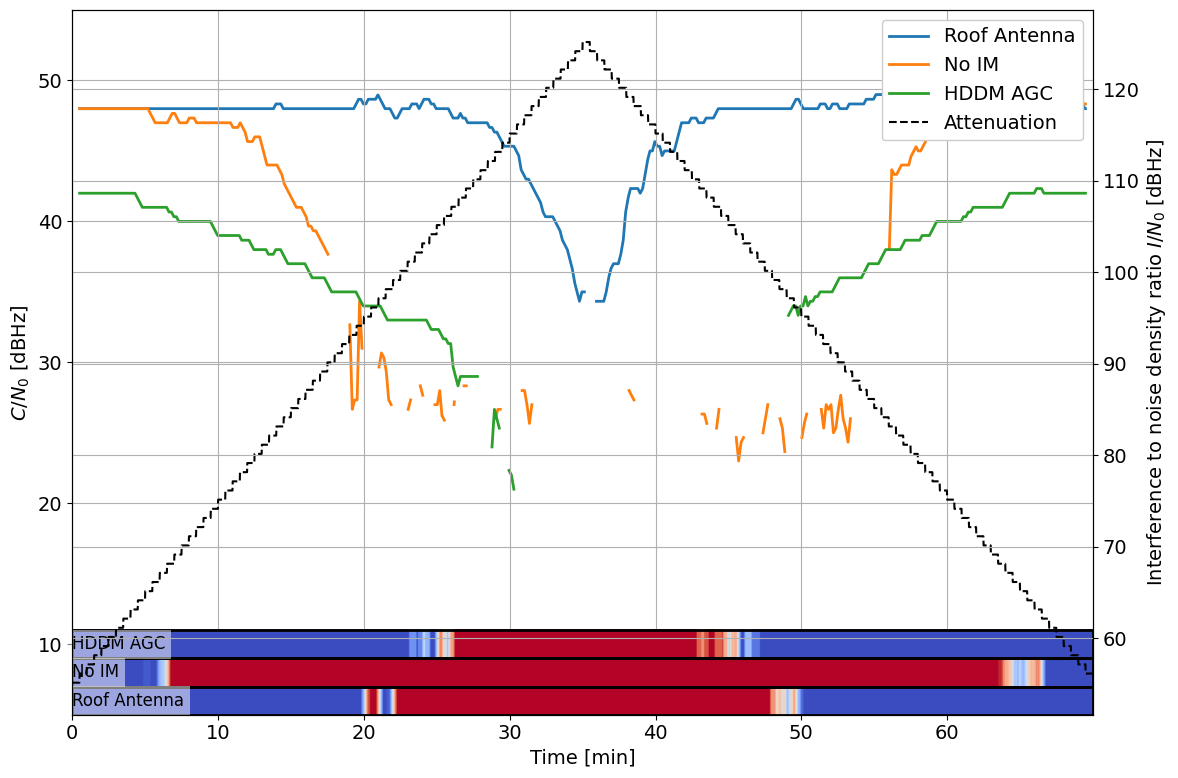

Supplement: Supplementary file 1 [file sensors-22-00679-s001.zip › results/Galileo/E1BC/Chirp_LinearFast_BW10/LC_Bar_SVID26_CN0.png]

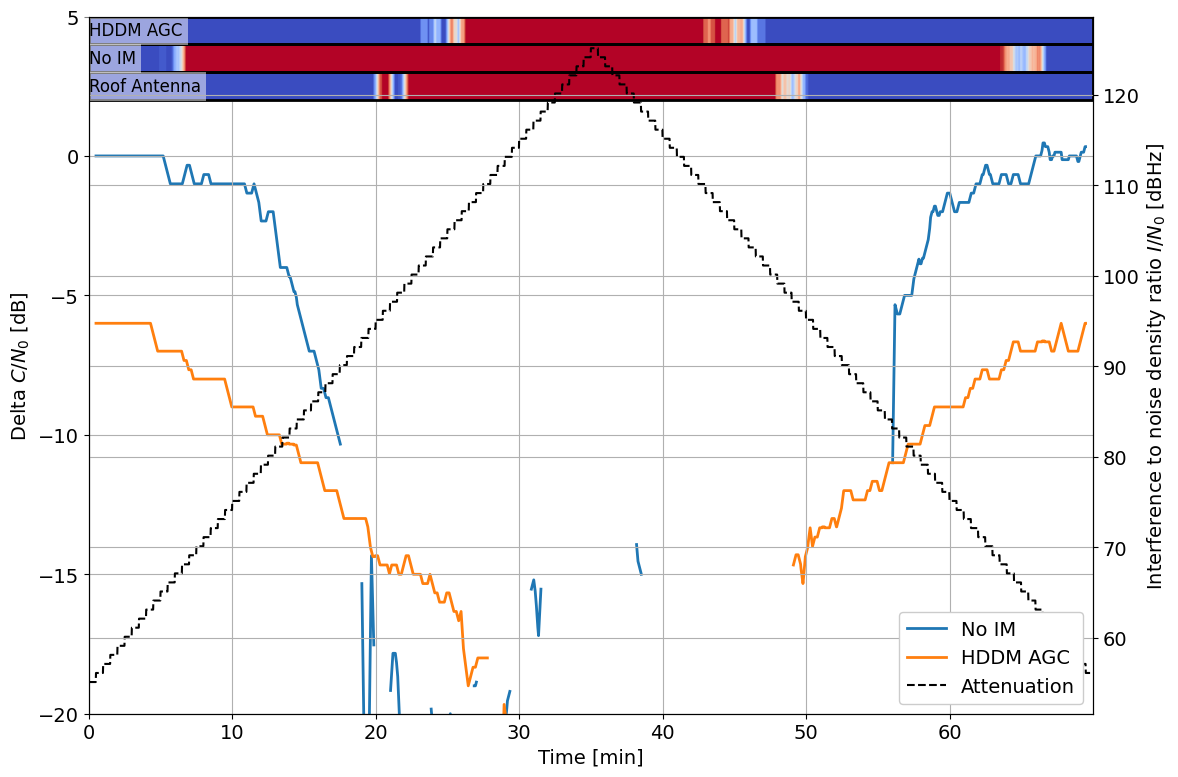

Supplement: Supplementary file 1 [file sensors-22-00679-s001.zip › results/Galileo/E1BC/Chirp_LinearFast_BW10/LC_Bar_SVID26_DeltaCN0.png]

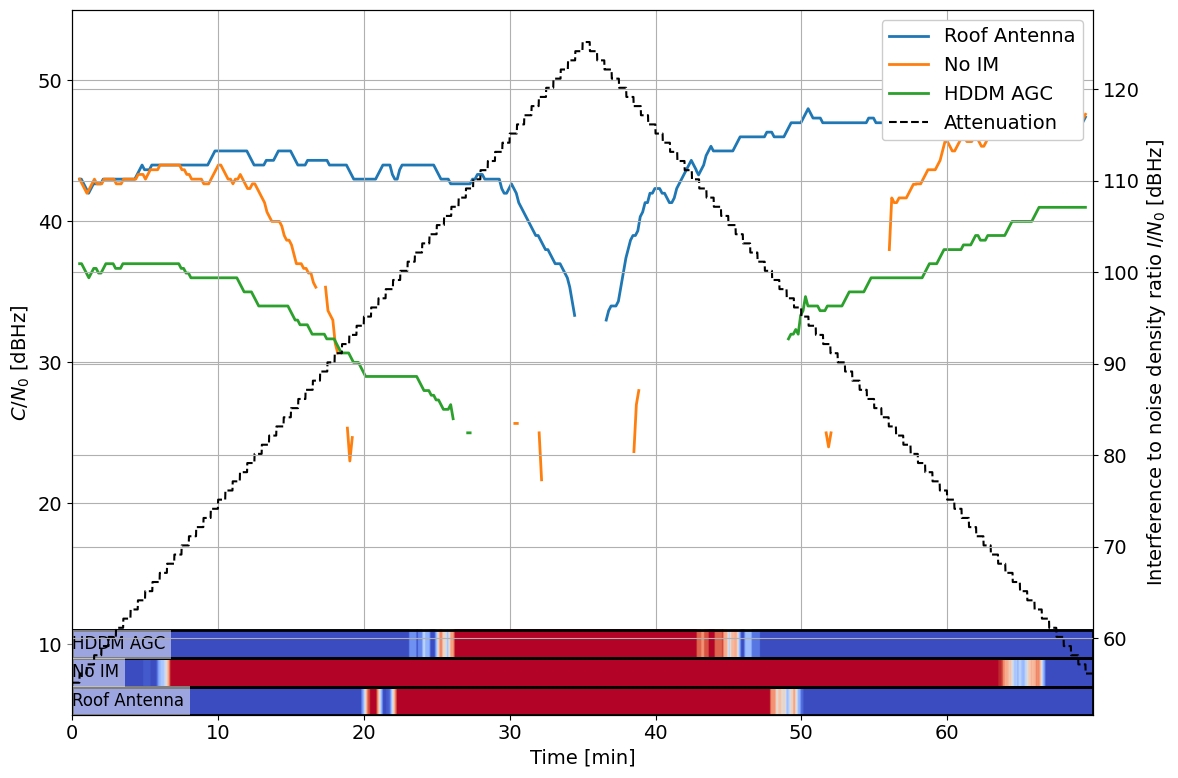

Supplement: Supplementary file 1 [file sensors-22-00679-s001.zip › results/Galileo/E1BC/Chirp_LinearFast_BW10/LC_Bar_SVID31_CN0.png]

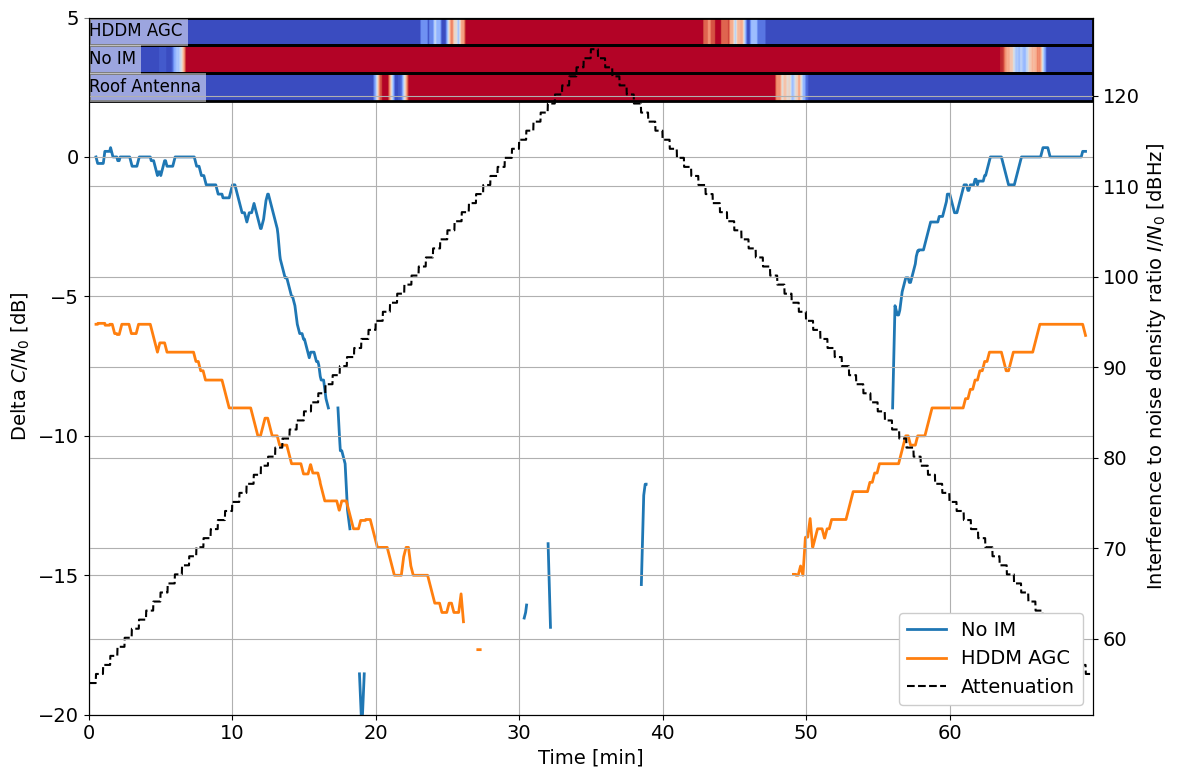

Supplement: Supplementary file 1 [file sensors-22-00679-s001.zip › results/Galileo/E1BC/Chirp_LinearFast_BW10/LC_Bar_SVID31_DeltaCN0.png]

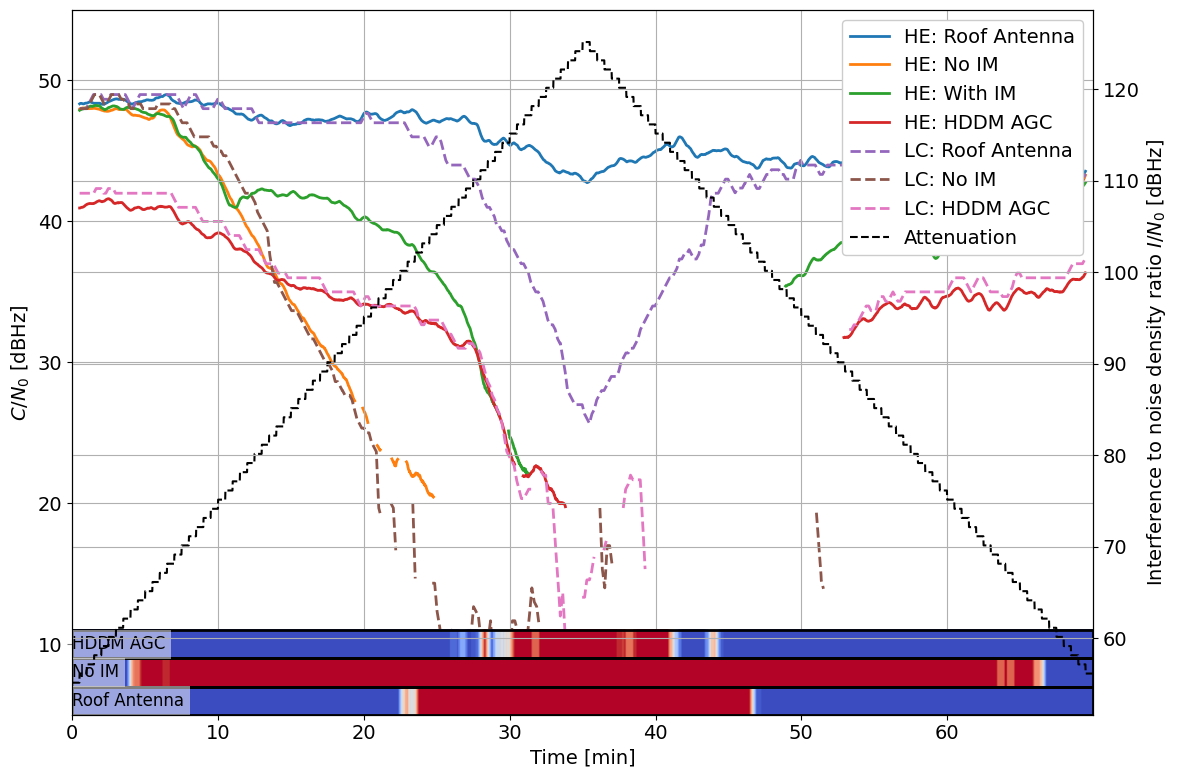

Supplement: Supplementary file 1 [file sensors-22-00679-s001.zip › results/Galileo/E1BC/Chirp_LinearMedium_BW10/HELC_Bar_SVID1_CN0.png]

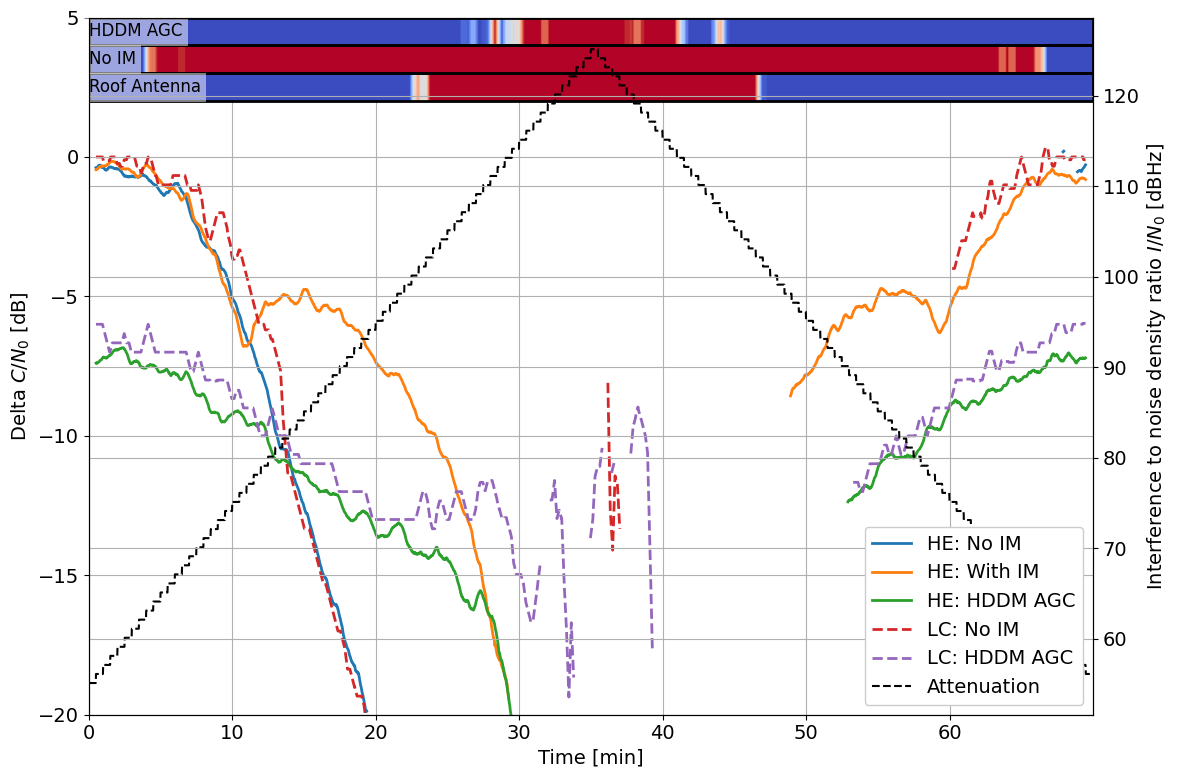

Supplement: Supplementary file 1 [file sensors-22-00679-s001.zip › results/Galileo/E1BC/Chirp_LinearMedium_BW10/HELC_Bar_SVID1_DeltaCN0.png]

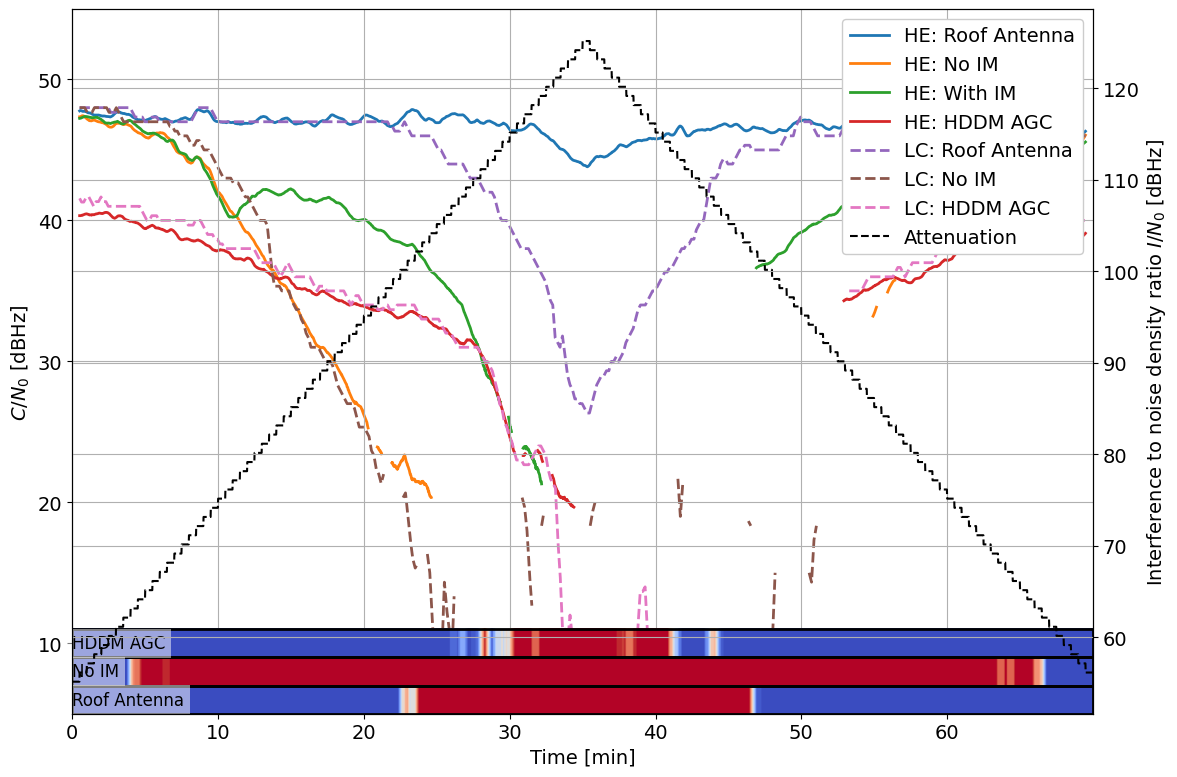

Supplement: Supplementary file 1 [file sensors-22-00679-s001.zip › results/Galileo/E1BC/Chirp_LinearMedium_BW10/HELC_Bar_SVID26_CN0.png]

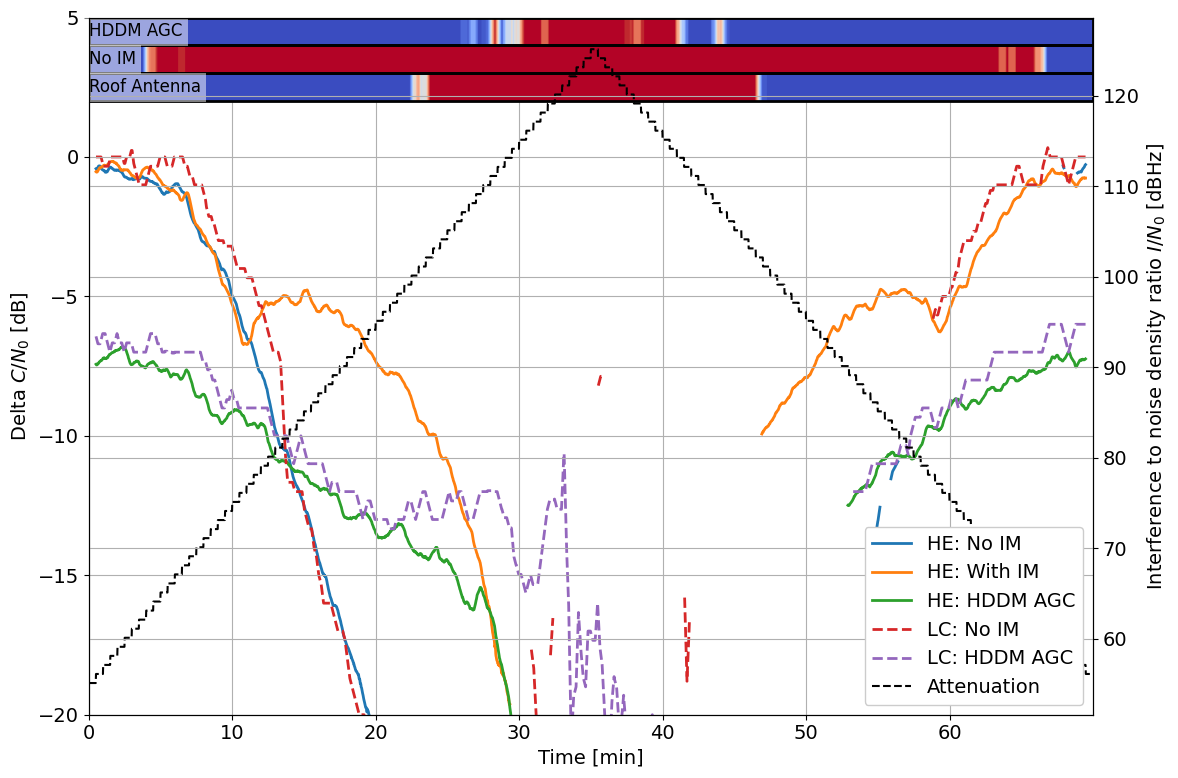

Supplement: Supplementary file 1 [file sensors-22-00679-s001.zip › results/Galileo/E1BC/Chirp_LinearMedium_BW10/HELC_Bar_SVID26_DeltaCN0.png]

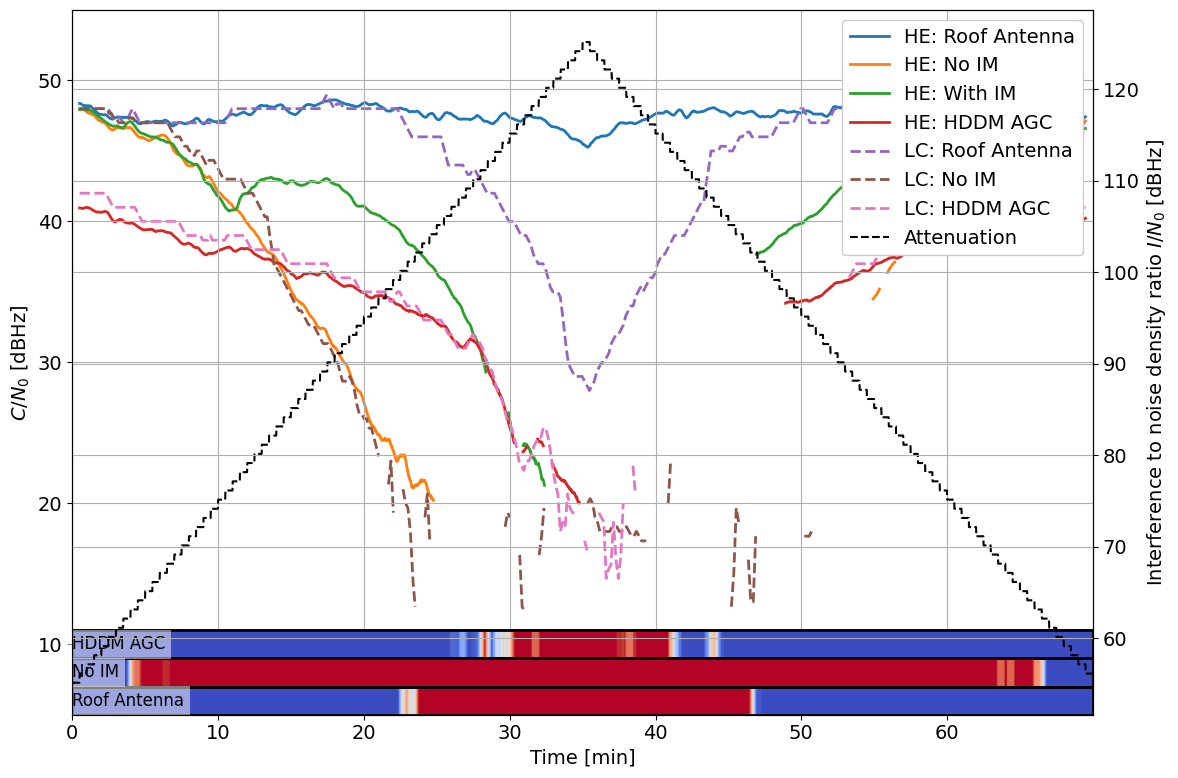

Supplement: Supplementary file 1 [file sensors-22-00679-s001.zip › results/Galileo/E1BC/Chirp_LinearMedium_BW10/HELC_Bar_SVID31_CN0.png]

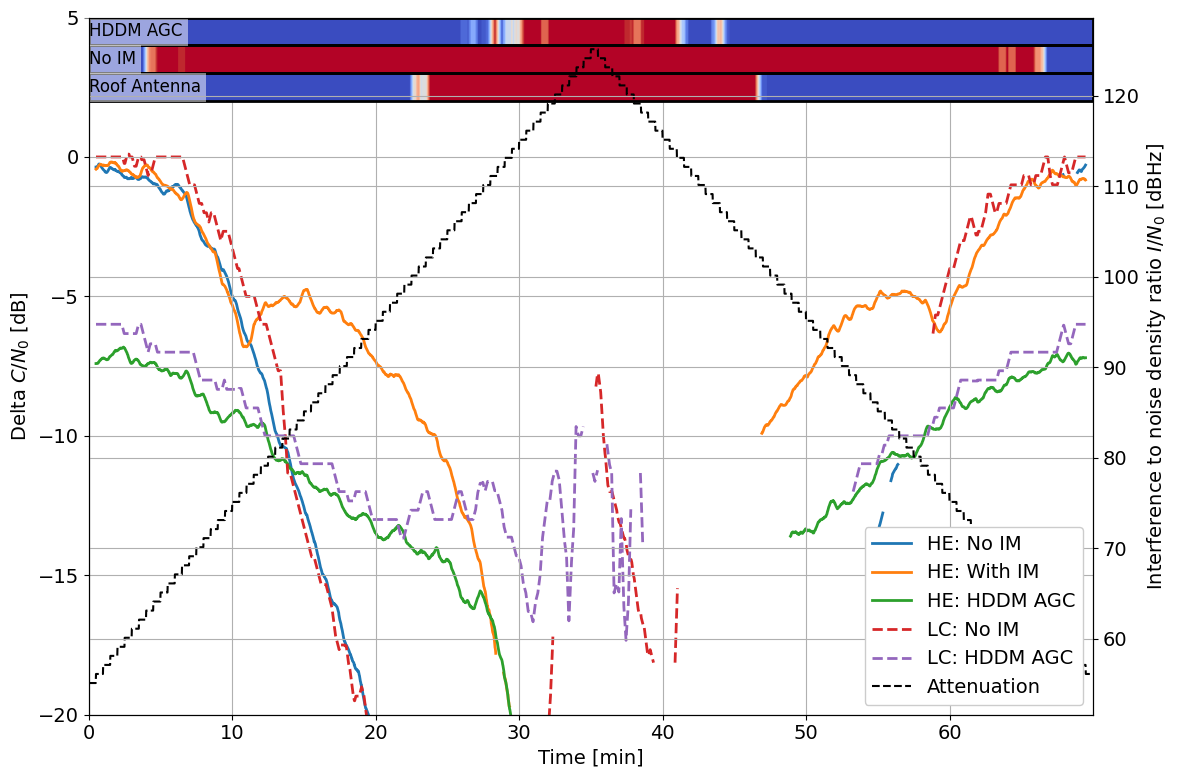

Supplement: Supplementary file 1 [file sensors-22-00679-s001.zip › results/Galileo/E1BC/Chirp_LinearMedium_BW10/HELC_Bar_SVID31_DeltaCN0.png]

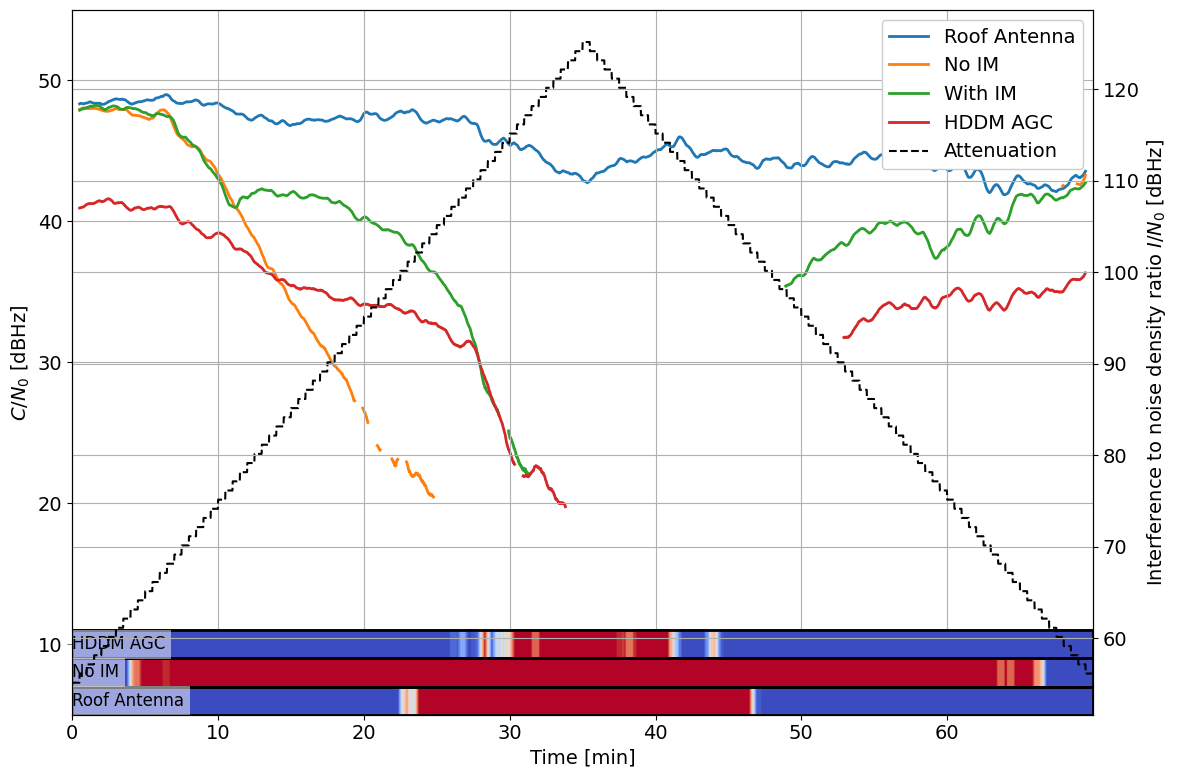

Supplement: Supplementary file 1 [file sensors-22-00679-s001.zip › results/Galileo/E1BC/Chirp_LinearMedium_BW10/HE_Bar_SVID1_CN0.png]

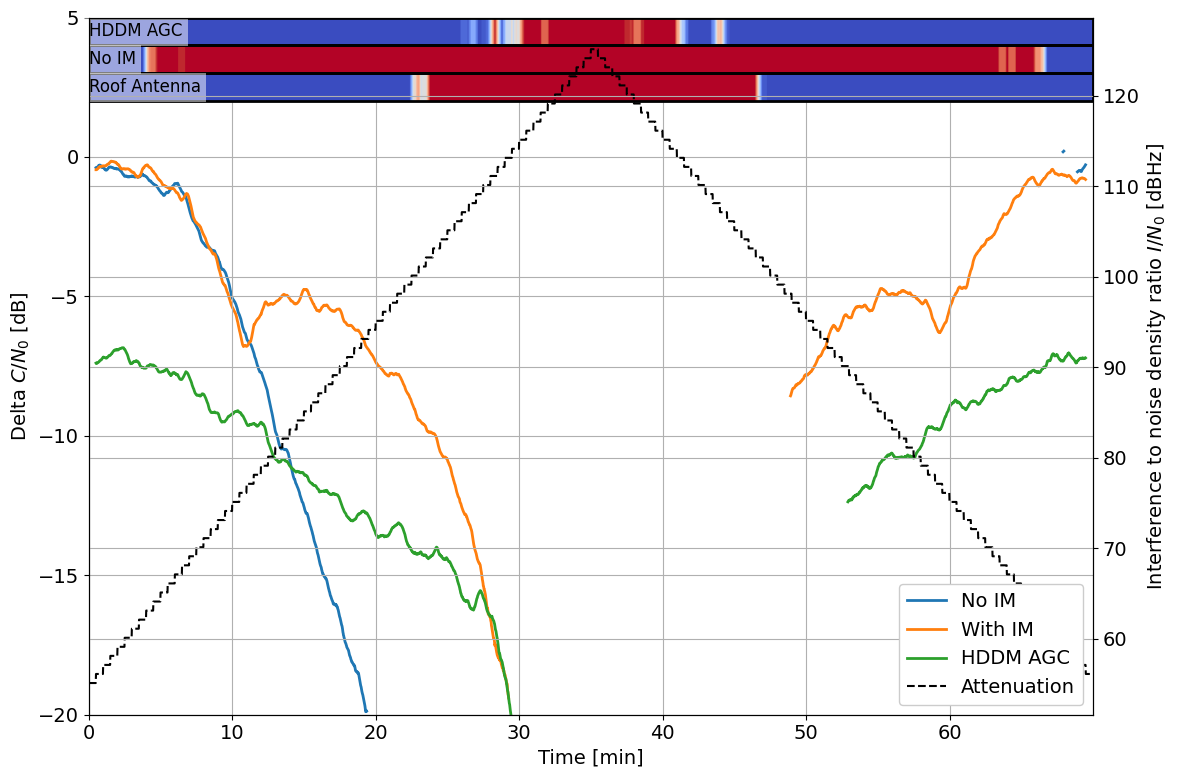

Supplement: Supplementary file 1 [file sensors-22-00679-s001.zip › results/Galileo/E1BC/Chirp_LinearMedium_BW10/HE_Bar_SVID1_DeltaCN0.png]

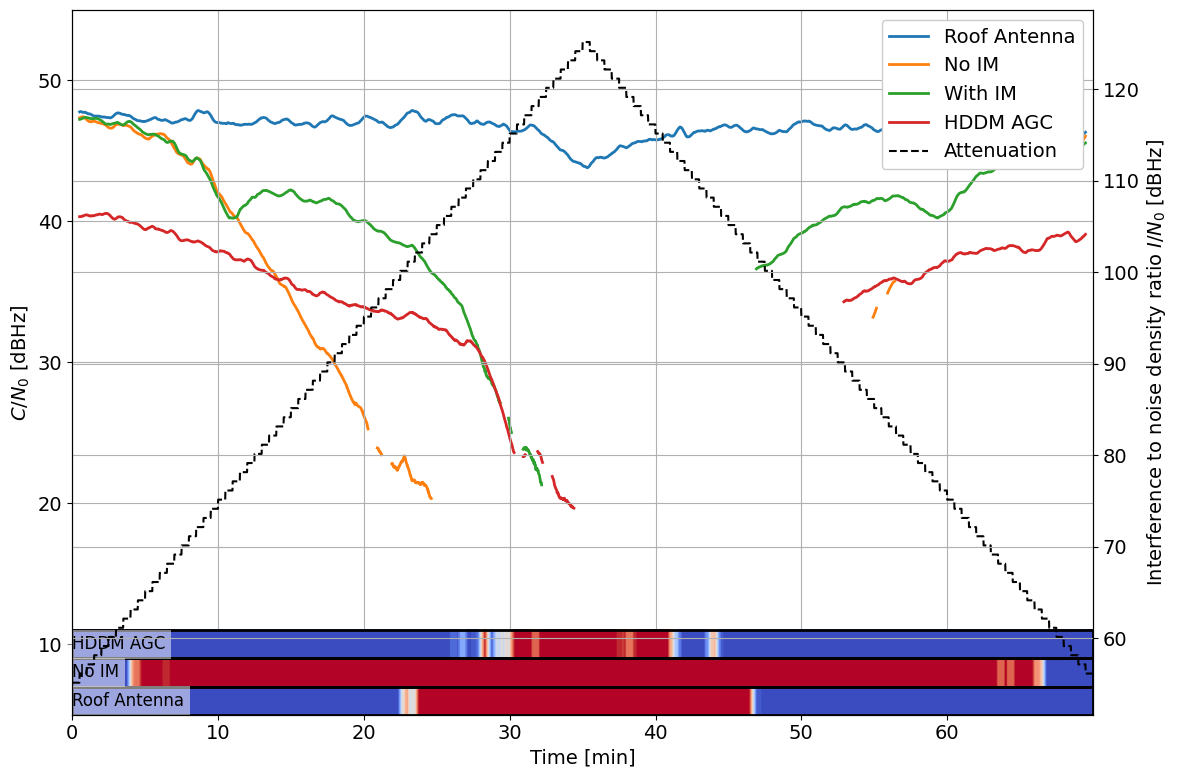

Supplement: Supplementary file 1 [file sensors-22-00679-s001.zip › results/Galileo/E1BC/Chirp_LinearMedium_BW10/HE_Bar_SVID26_CN0.png]

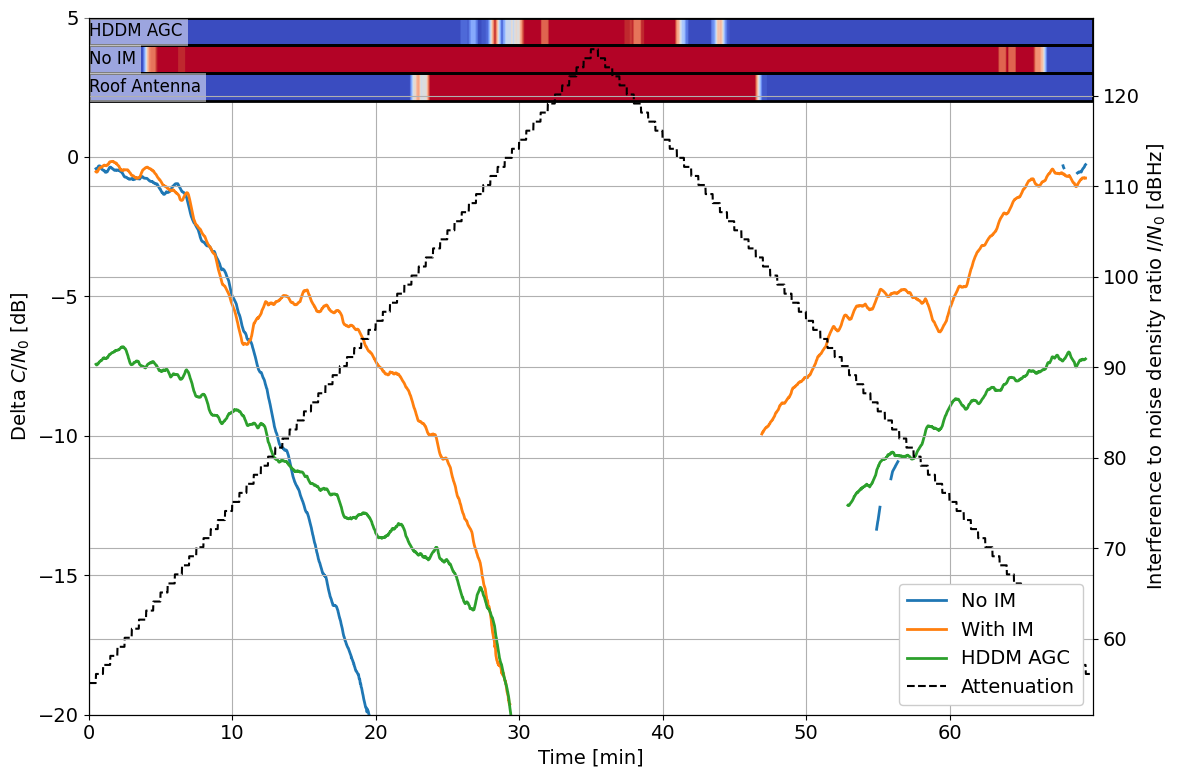

Supplement: Supplementary file 1 [file sensors-22-00679-s001.zip › results/Galileo/E1BC/Chirp_LinearMedium_BW10/HE_Bar_SVID26_DeltaCN0.png]

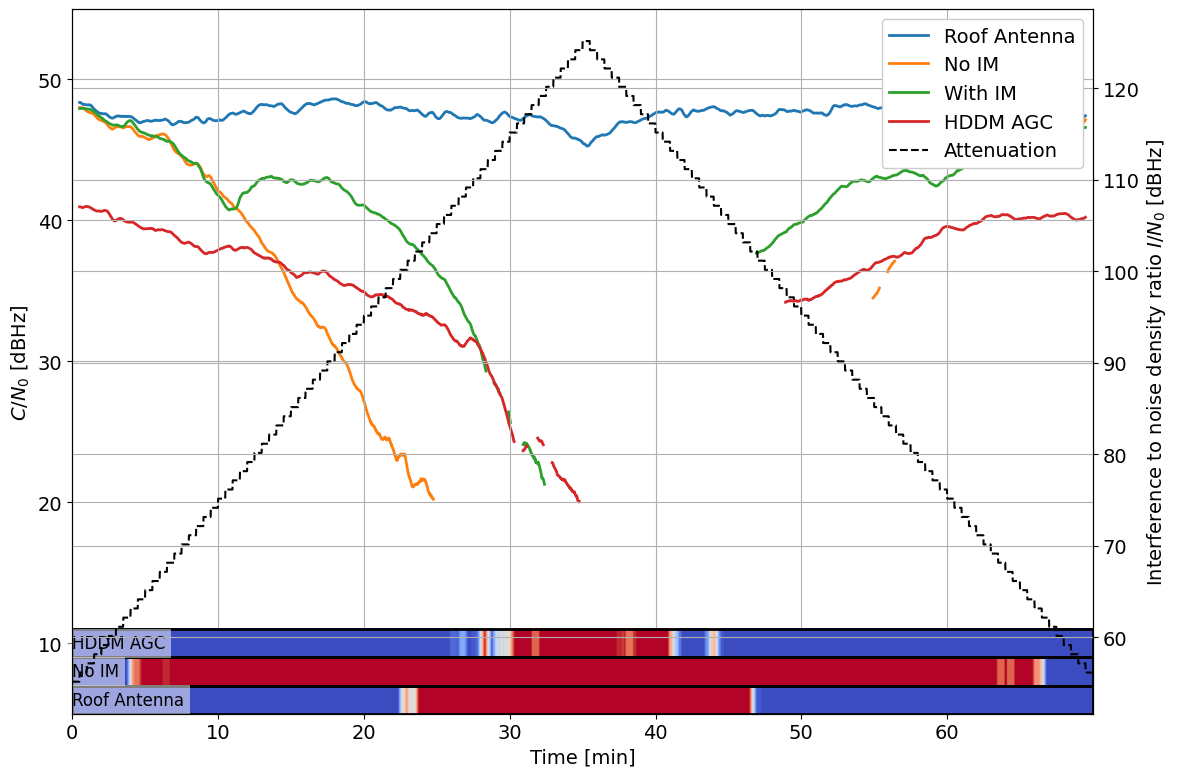

Supplement: Supplementary file 1 [file sensors-22-00679-s001.zip › results/Galileo/E1BC/Chirp_LinearMedium_BW10/HE_Bar_SVID31_CN0.png]

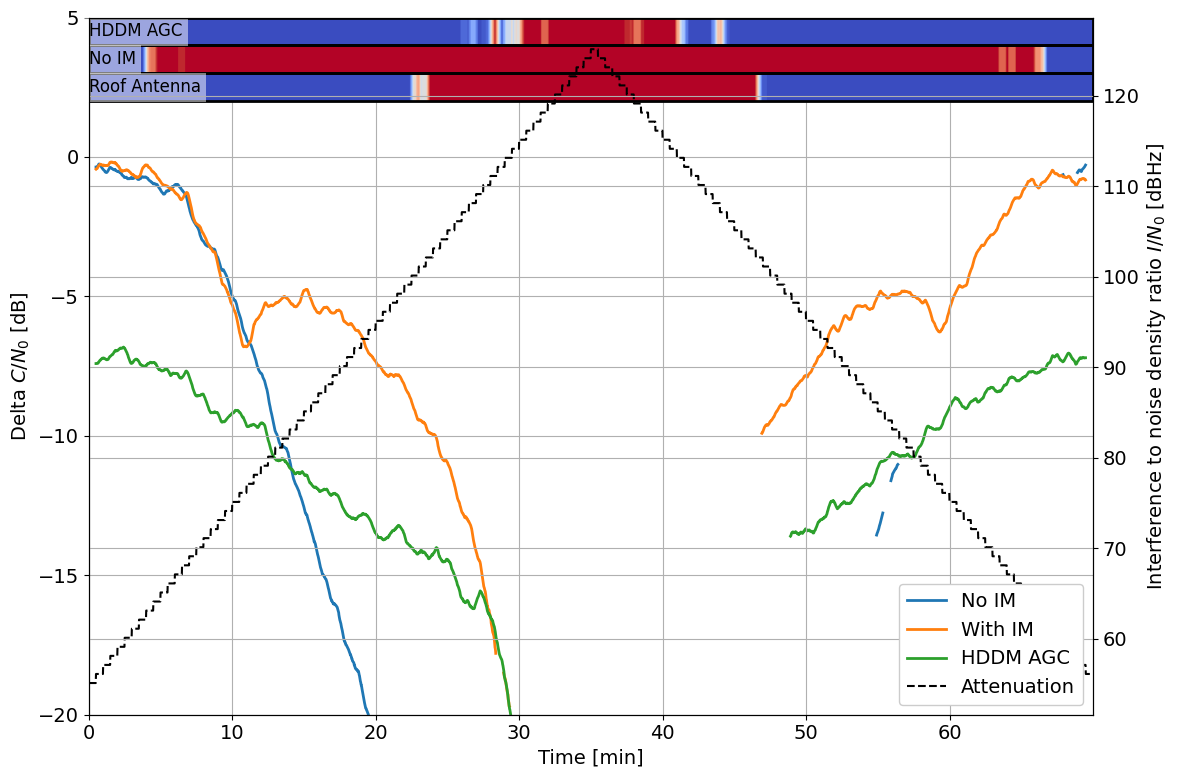

Supplement: Supplementary file 1 [file sensors-22-00679-s001.zip › results/Galileo/E1BC/Chirp_LinearMedium_BW10/HE_Bar_SVID31_DeltaCN0.png]

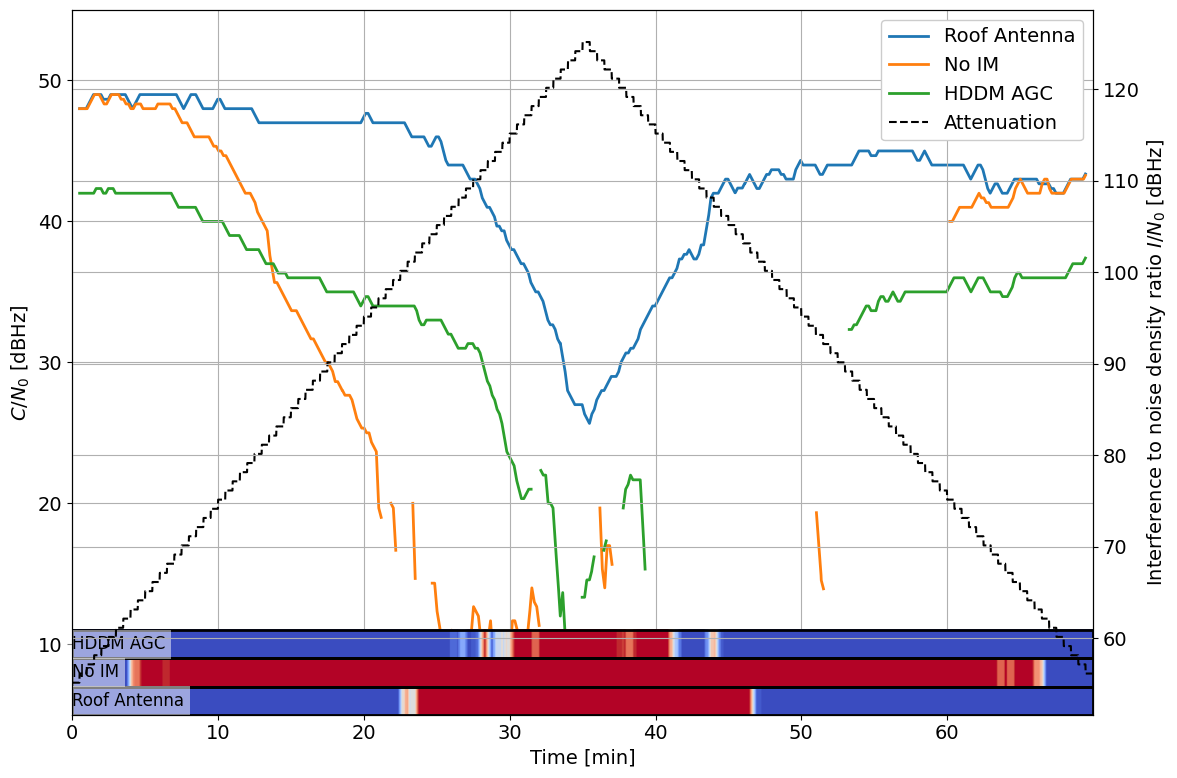

Supplement: Supplementary file 1 [file sensors-22-00679-s001.zip › results/Galileo/E1BC/Chirp_LinearMedium_BW10/LC_Bar_SVID1_CN0.png]

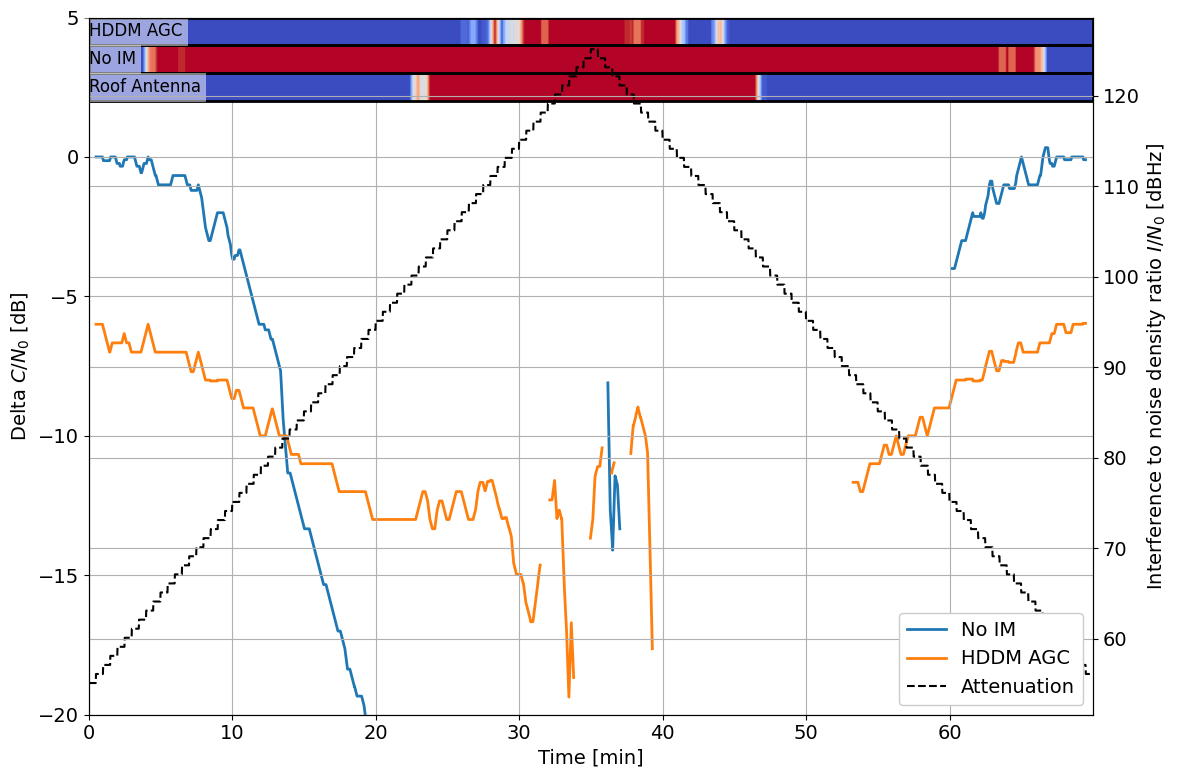

Supplement: Supplementary file 1 [file sensors-22-00679-s001.zip › results/Galileo/E1BC/Chirp_LinearMedium_BW10/LC_Bar_SVID1_DeltaCN0.png]

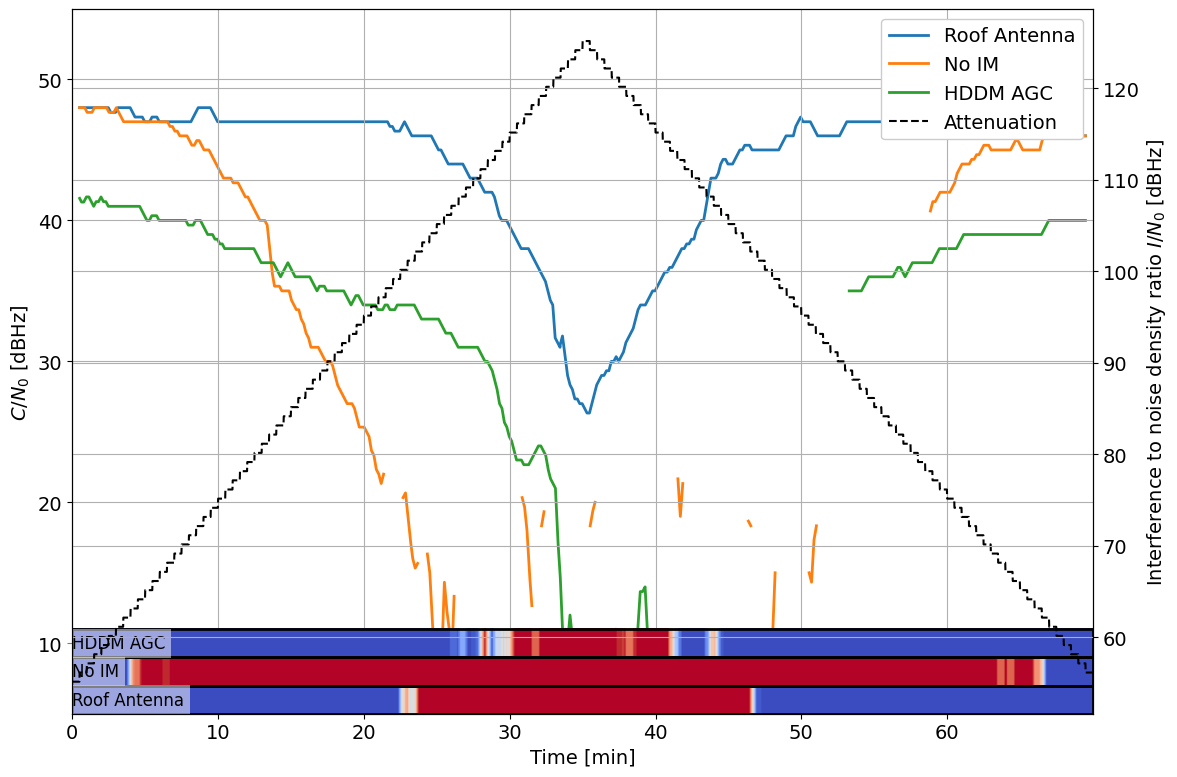

Supplement: Supplementary file 1 [file sensors-22-00679-s001.zip › results/Galileo/E1BC/Chirp_LinearMedium_BW10/LC_Bar_SVID26_CN0.png]

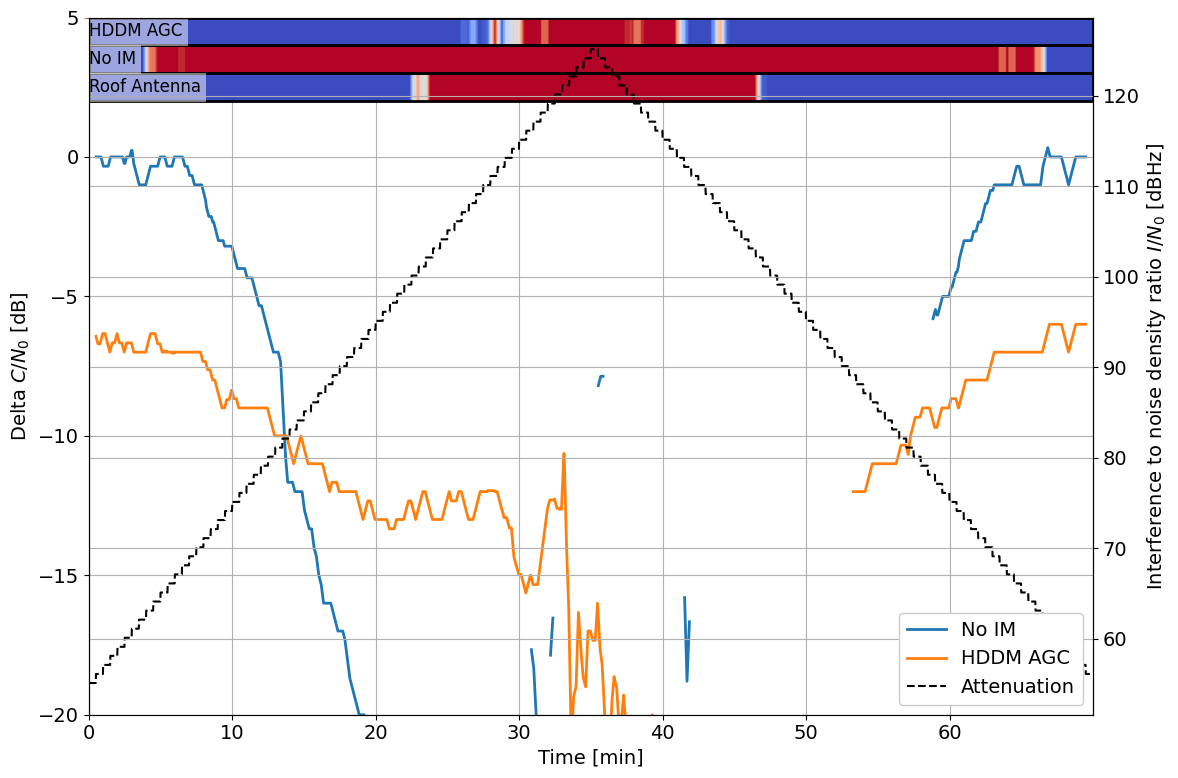

Supplement: Supplementary file 1 [file sensors-22-00679-s001.zip › results/Galileo/E1BC/Chirp_LinearMedium_BW10/LC_Bar_SVID26_DeltaCN0.png]

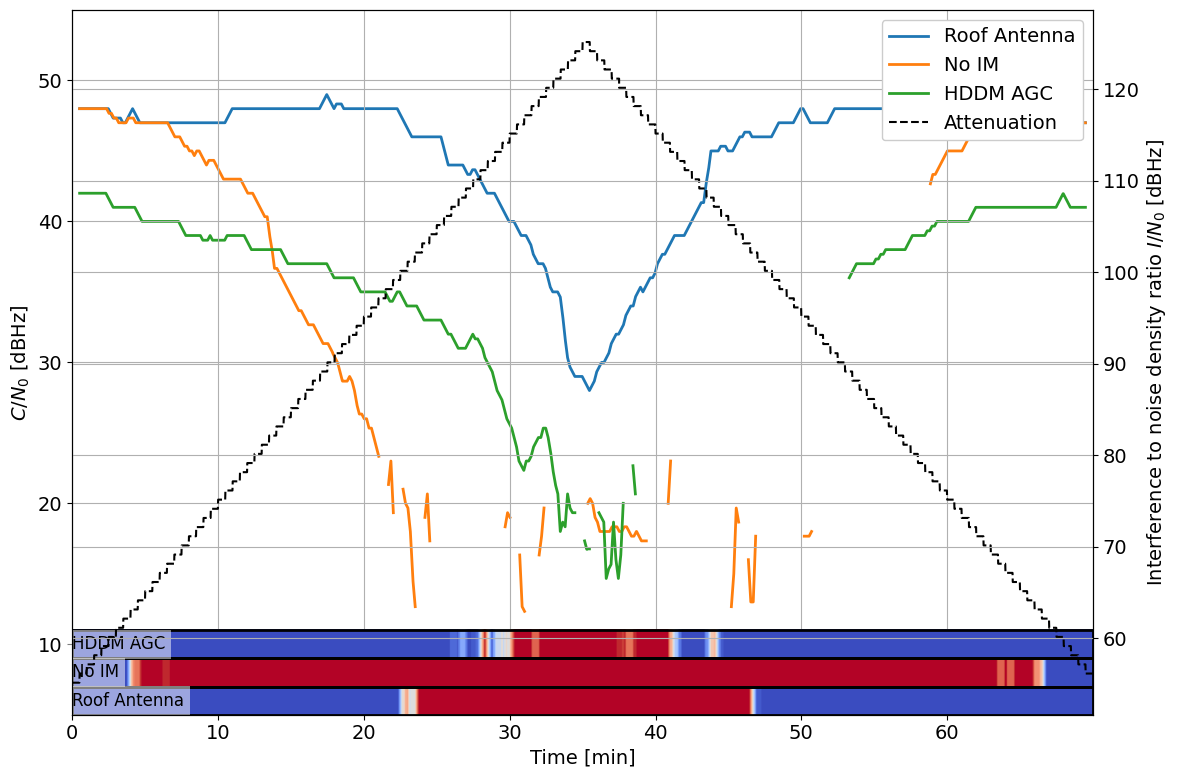

Supplement: Supplementary file 1 [file sensors-22-00679-s001.zip › results/Galileo/E1BC/Chirp_LinearMedium_BW10/LC_Bar_SVID31_CN0.png]

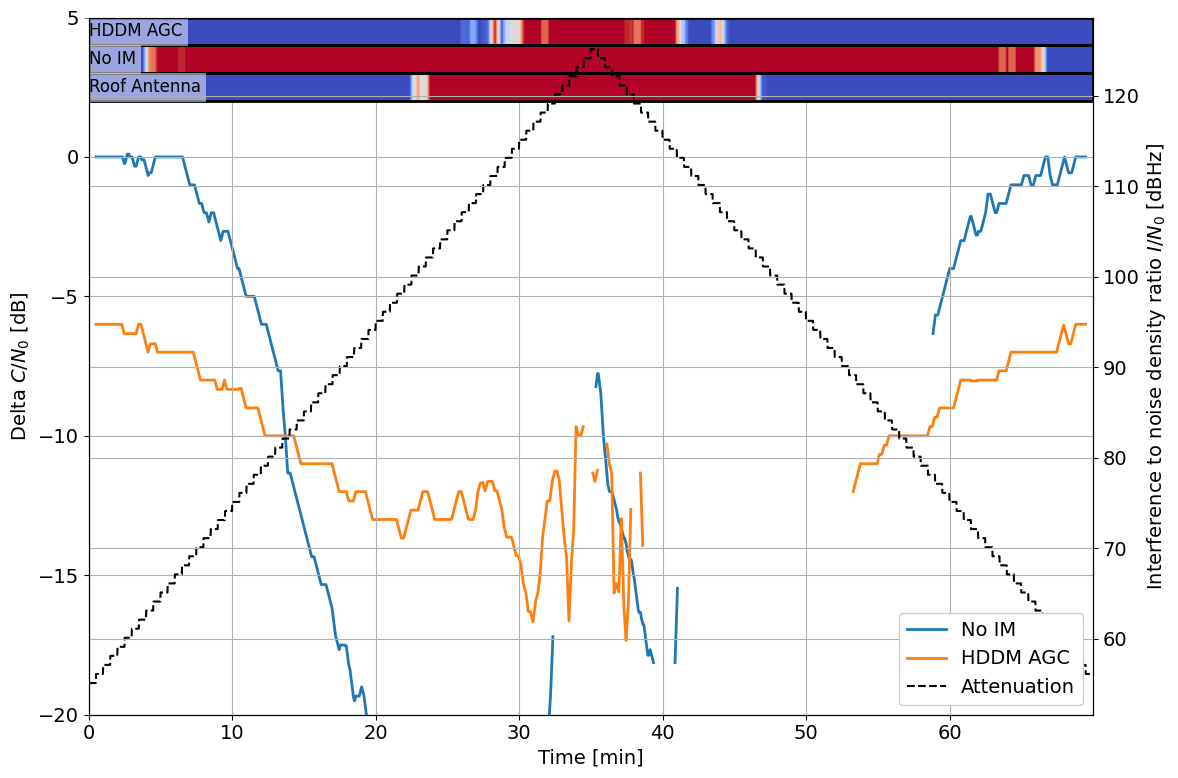

Supplement: Supplementary file 1 [file sensors-22-00679-s001.zip › results/Galileo/E1BC/Chirp_LinearMedium_BW10/LC_Bar_SVID31_DeltaCN0.png]

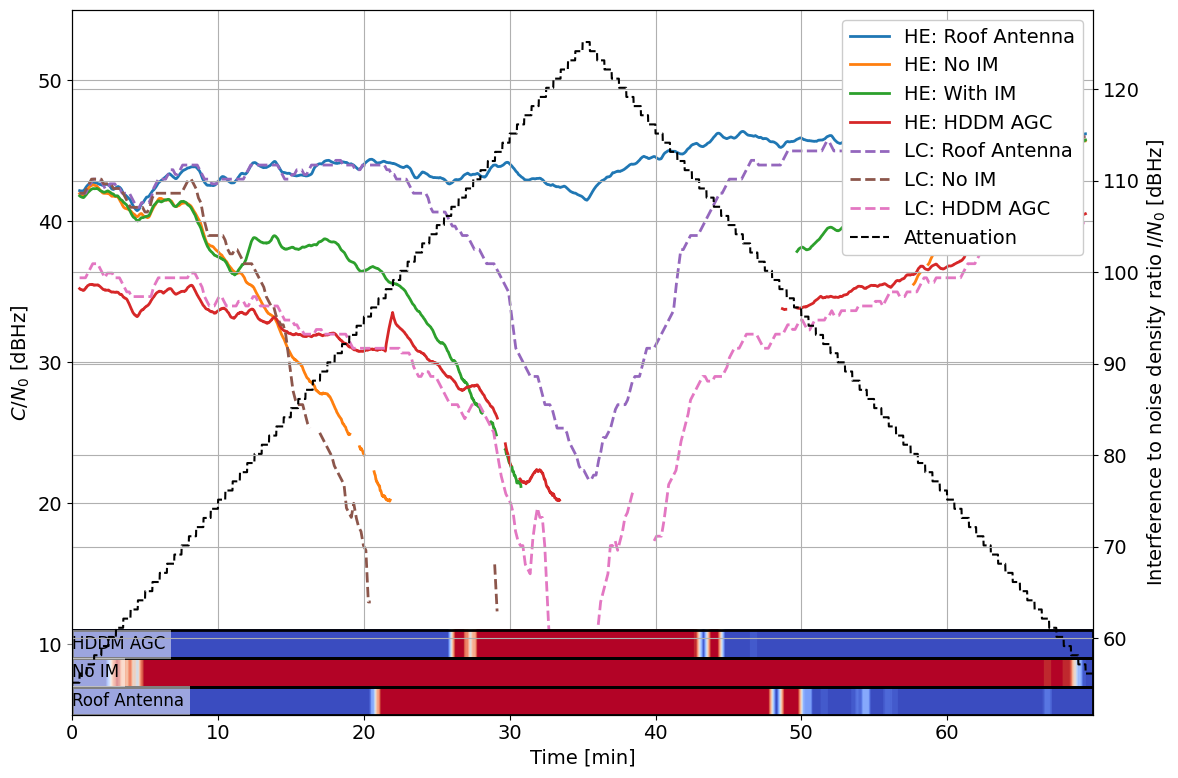

Supplement: Supplementary file 1 [file sensors-22-00679-s001.zip › results/Galileo/E1BC/Chirp_LinearSlow_BW10/HELC_Bar_SVID24_CN0.png]

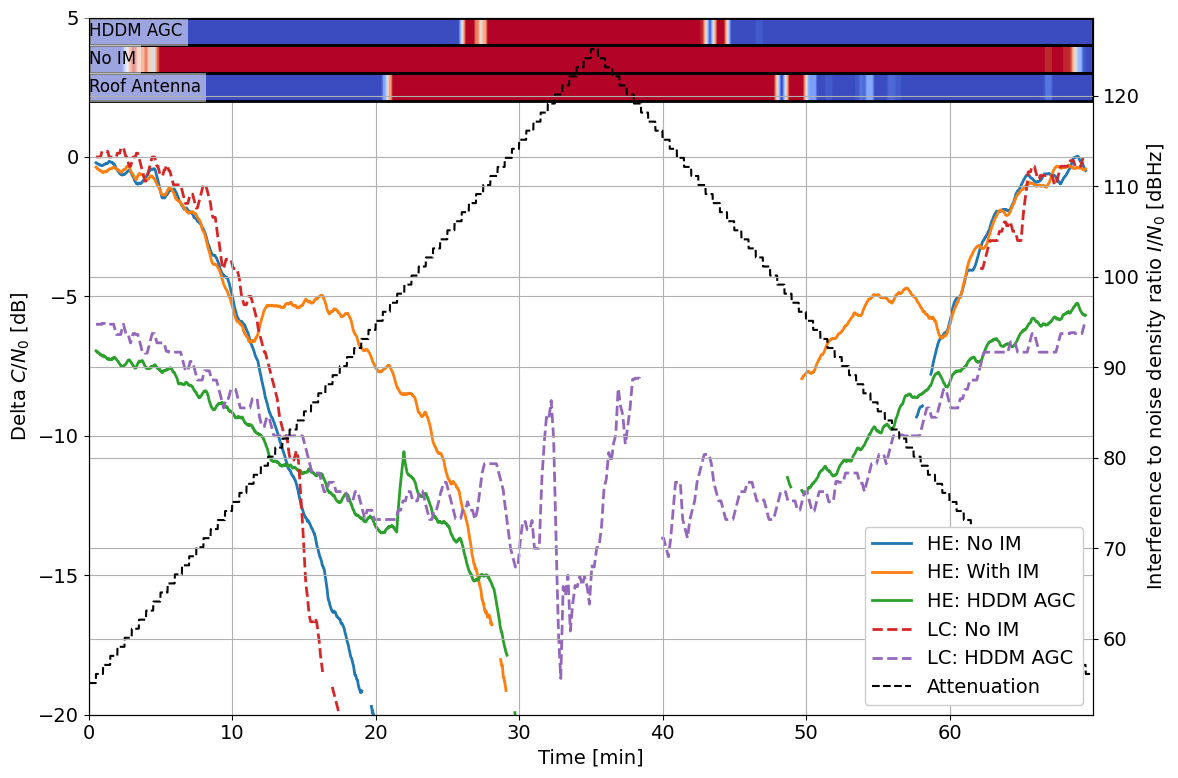

Supplement: Supplementary file 1 [file sensors-22-00679-s001.zip › results/Galileo/E1BC/Chirp_LinearSlow_BW10/HELC_Bar_SVID24_DeltaCN0.png]

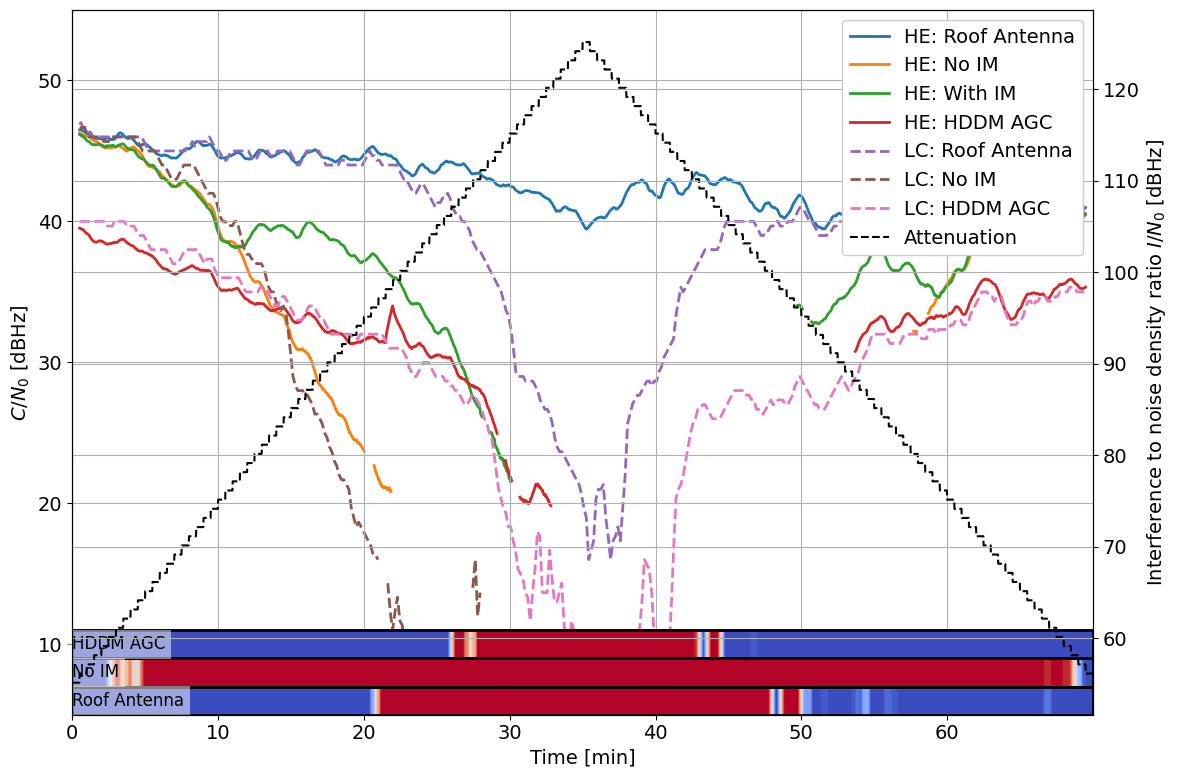

Supplement: Supplementary file 1 [file sensors-22-00679-s001.zip › results/Galileo/E1BC/Chirp_LinearSlow_BW10/HELC_Bar_SVID26_CN0.png]

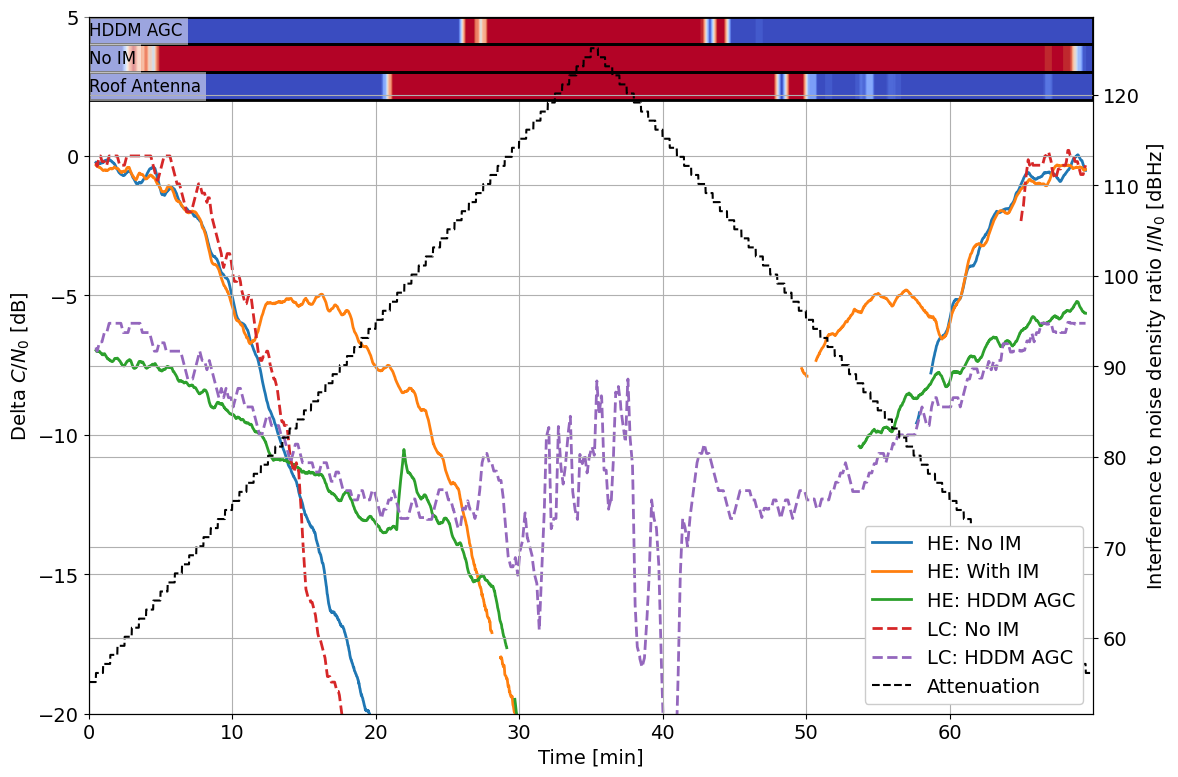

Supplement: Supplementary file 1 [file sensors-22-00679-s001.zip › results/Galileo/E1BC/Chirp_LinearSlow_BW10/HELC_Bar_SVID26_DeltaCN0.png]

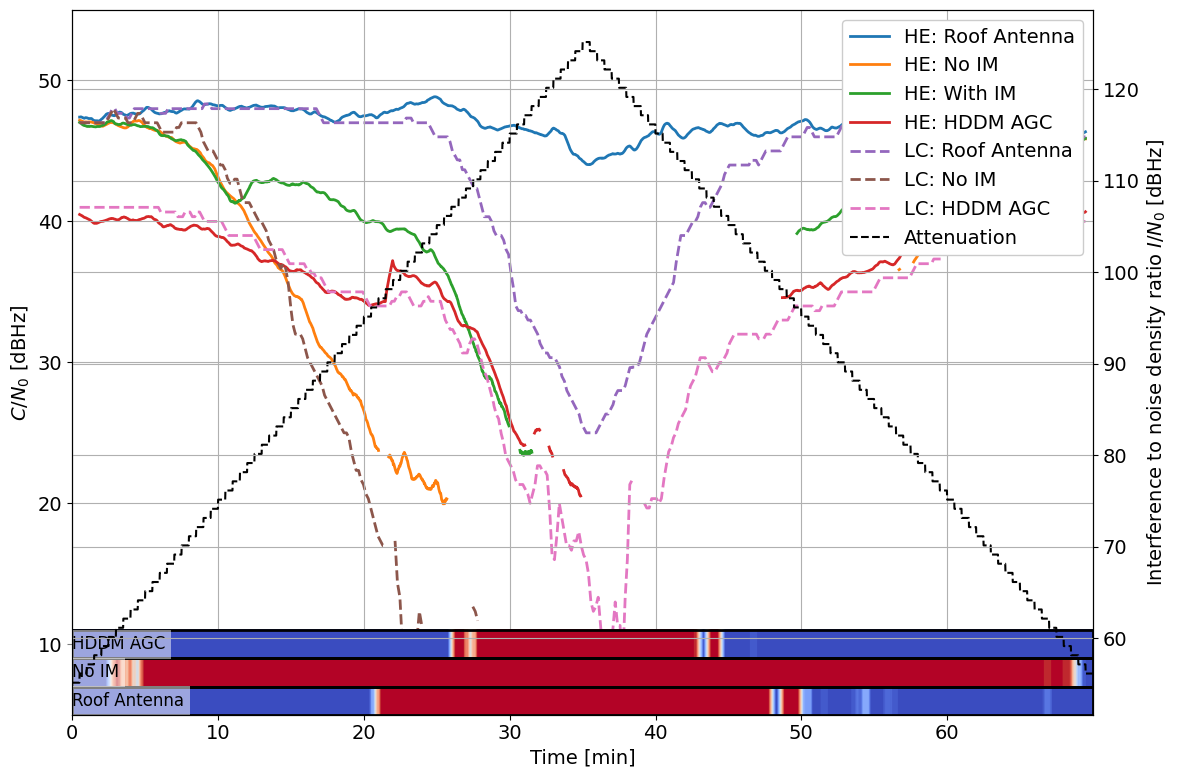

Supplement: Supplementary file 1 [file sensors-22-00679-s001.zip › results/Galileo/E1BC/Chirp_LinearSlow_BW10/HELC_Bar_SVID31_CN0.png]

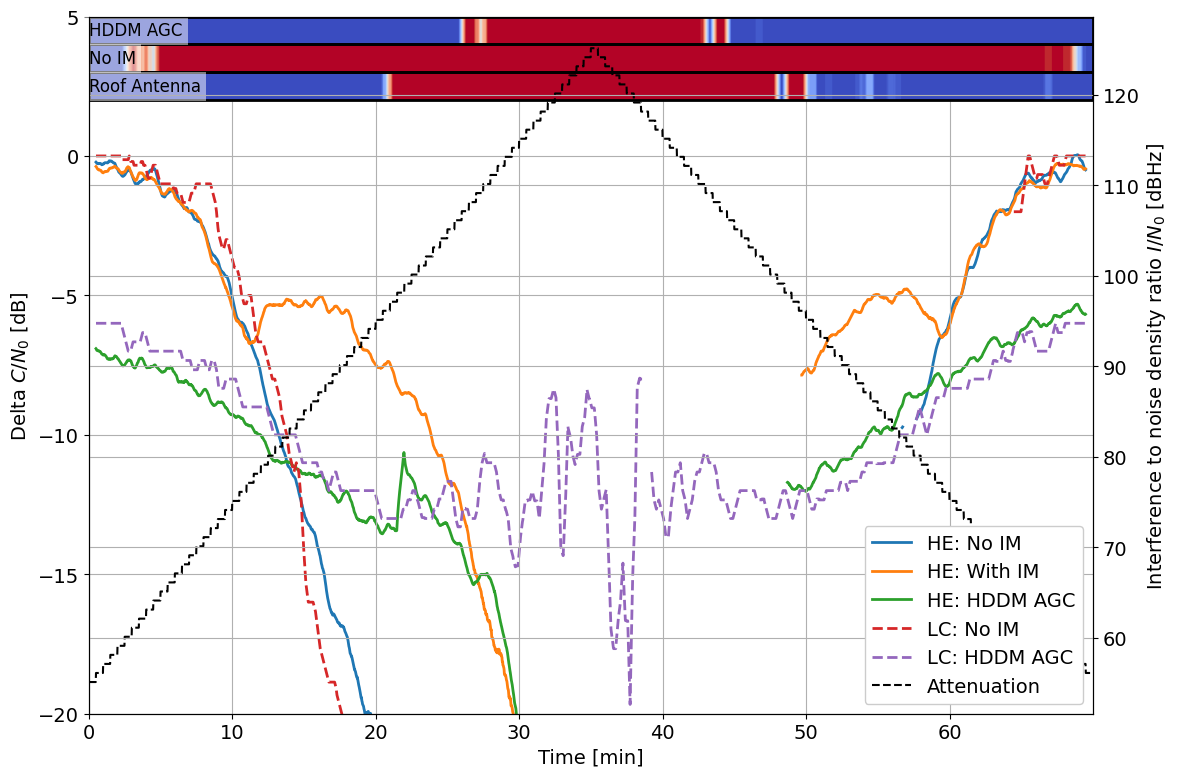

Supplement: Supplementary file 1 [file sensors-22-00679-s001.zip › results/Galileo/E1BC/Chirp_LinearSlow_BW10/HELC_Bar_SVID31_DeltaCN0.png]

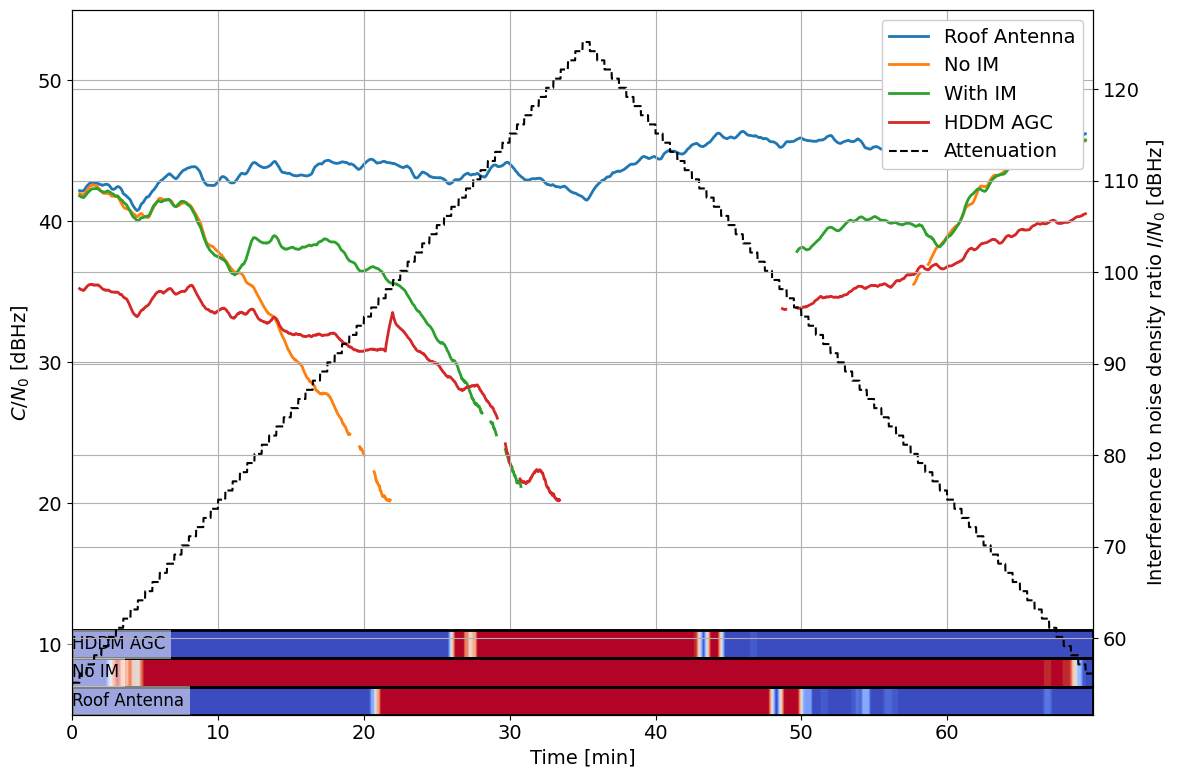

Supplement: Supplementary file 1 [file sensors-22-00679-s001.zip › results/Galileo/E1BC/Chirp_LinearSlow_BW10/HE_Bar_SVID24_CN0.png]

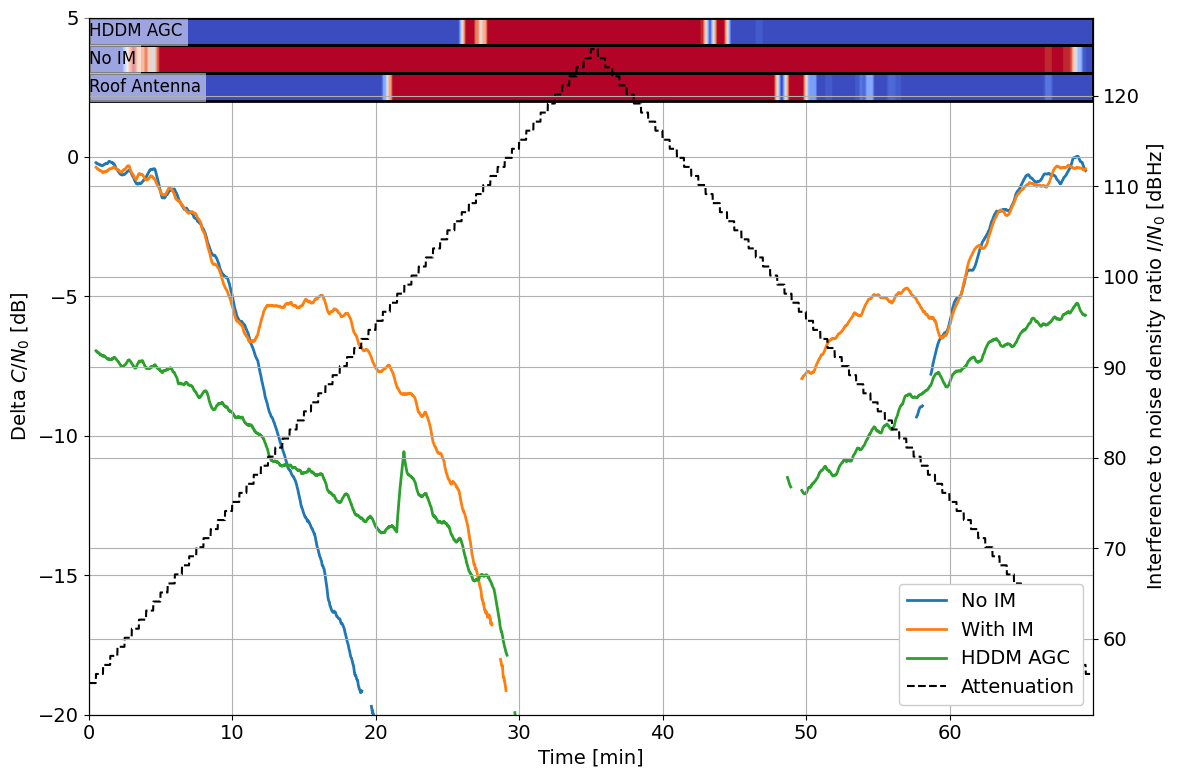

Supplement: Supplementary file 1 [file sensors-22-00679-s001.zip › results/Galileo/E1BC/Chirp_LinearSlow_BW10/HE_Bar_SVID24_DeltaCN0.png]

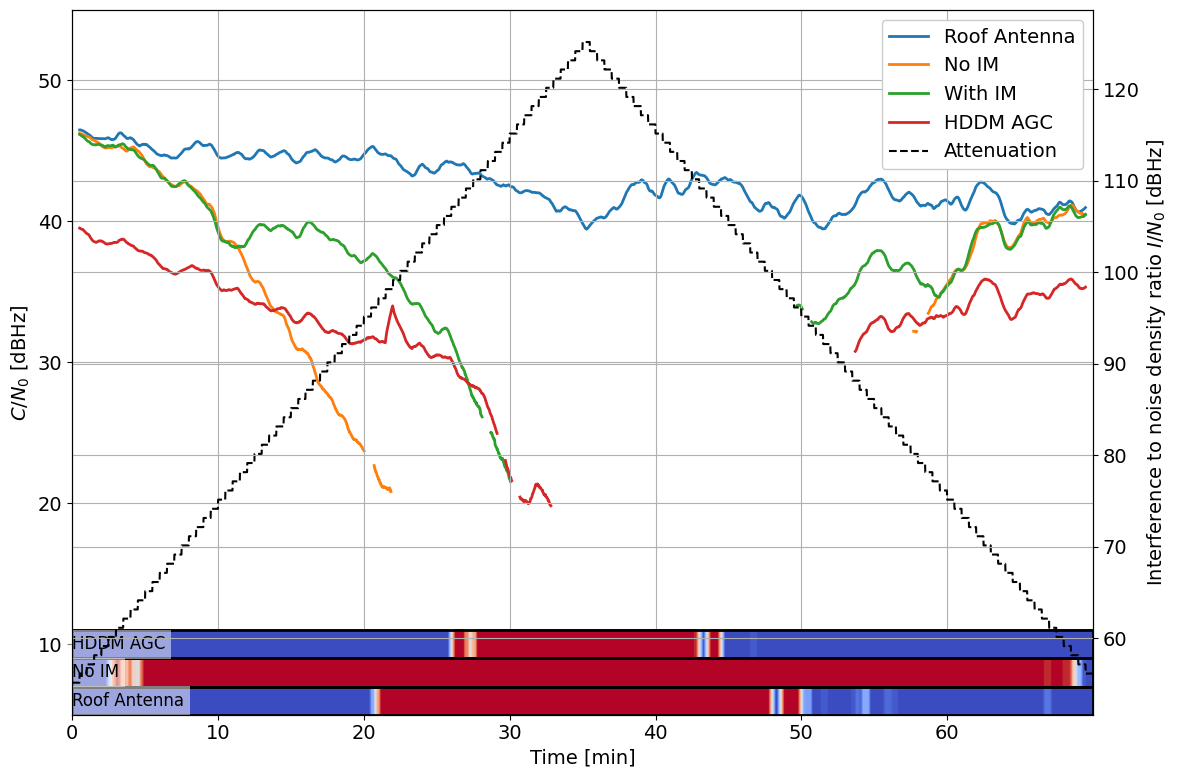

Supplement: Supplementary file 1 [file sensors-22-00679-s001.zip › results/Galileo/E1BC/Chirp_LinearSlow_BW10/HE_Bar_SVID26_CN0.png]

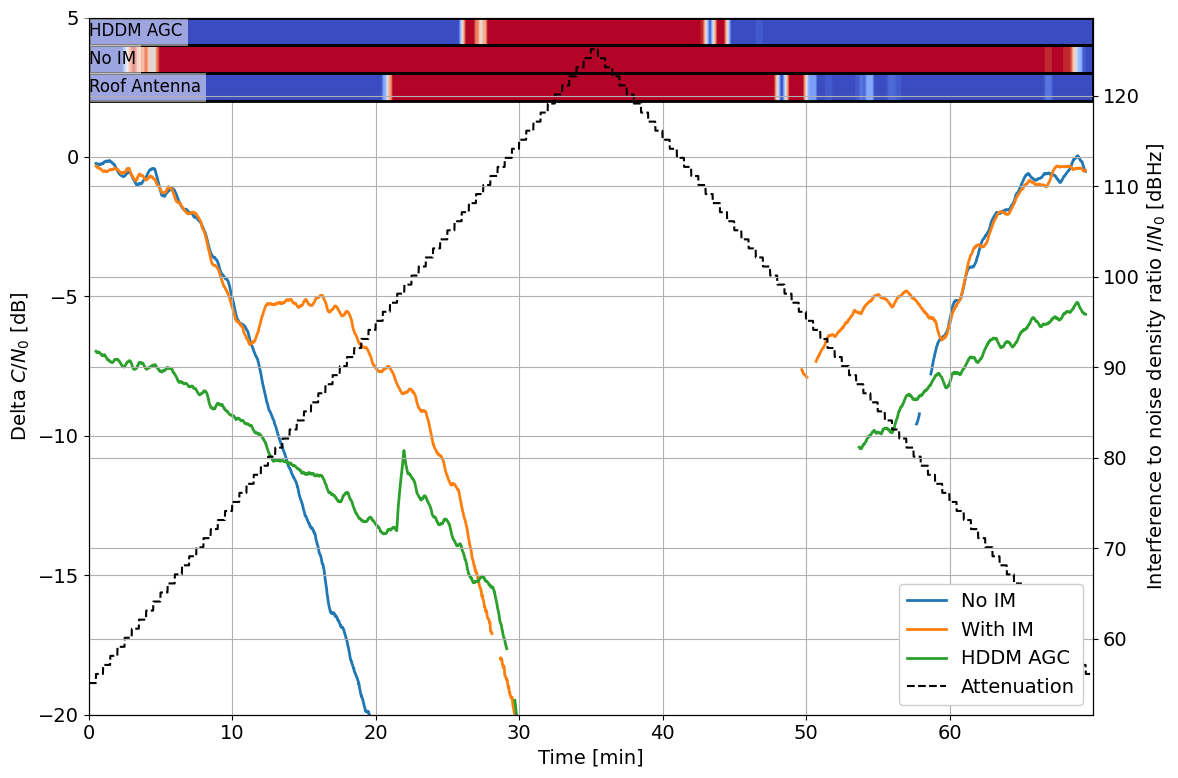

Supplement: Supplementary file 1 [file sensors-22-00679-s001.zip › results/Galileo/E1BC/Chirp_LinearSlow_BW10/HE_Bar_SVID26_DeltaCN0.png]

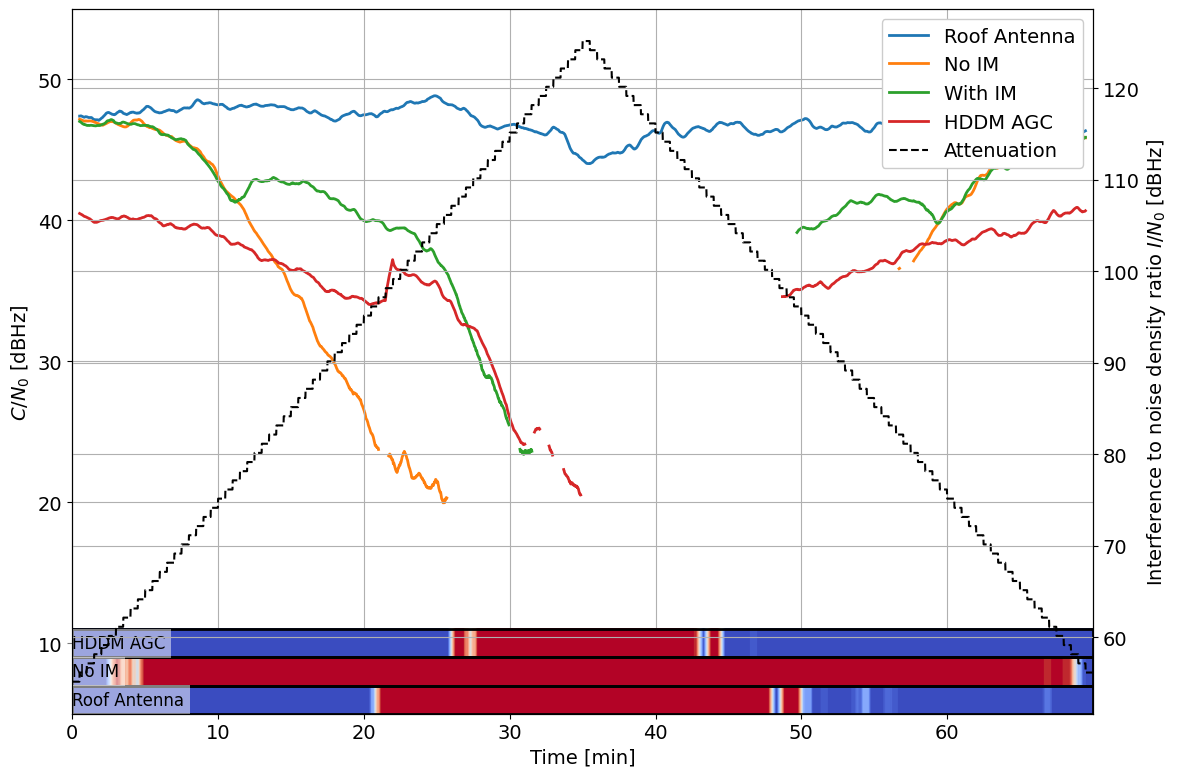

Supplement: Supplementary file 1 [file sensors-22-00679-s001.zip › results/Galileo/E1BC/Chirp_LinearSlow_BW10/HE_Bar_SVID31_CN0.png]

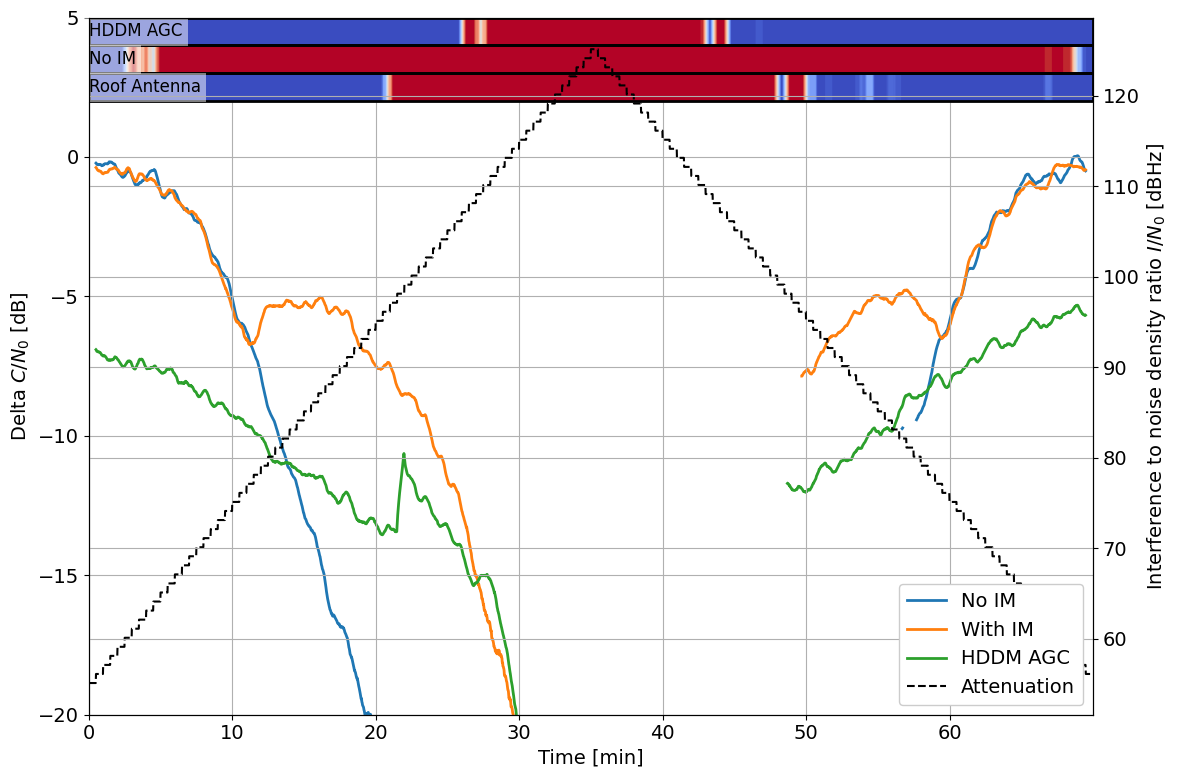

Supplement: Supplementary file 1 [file sensors-22-00679-s001.zip › results/Galileo/E1BC/Chirp_LinearSlow_BW10/HE_Bar_SVID31_DeltaCN0.png]

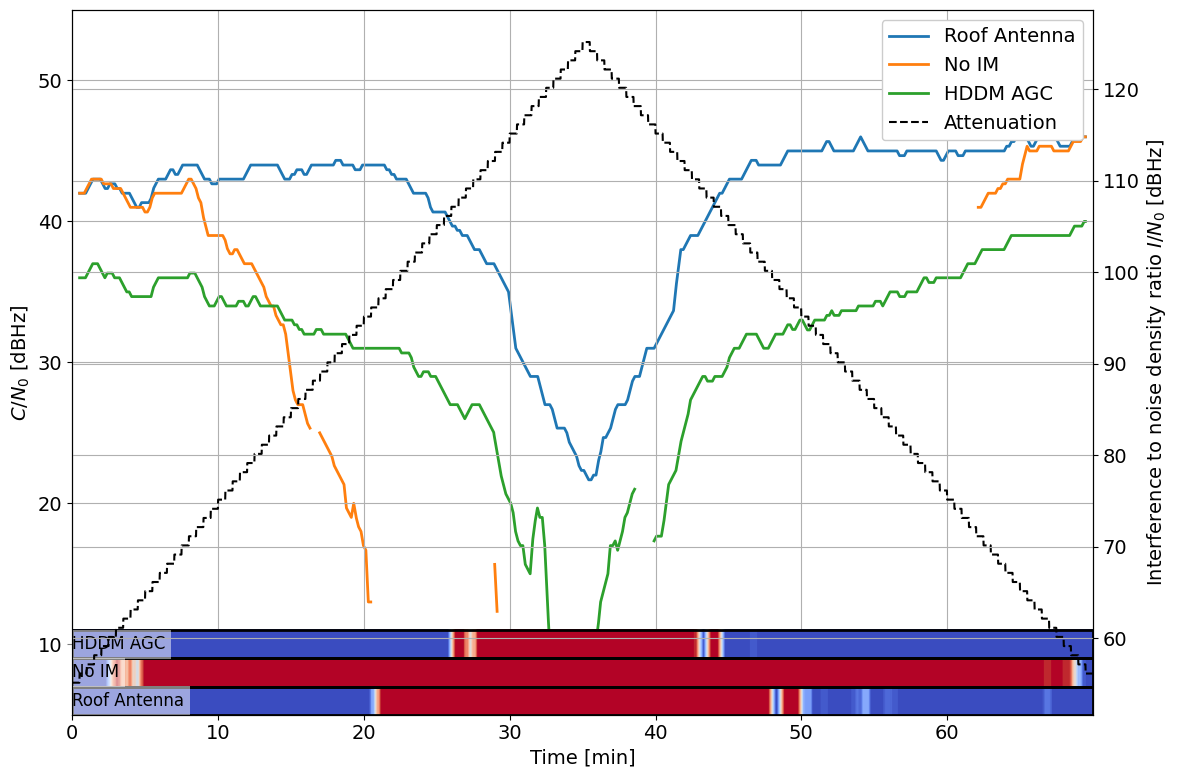

Supplement: Supplementary file 1 [file sensors-22-00679-s001.zip › results/Galileo/E1BC/Chirp_LinearSlow_BW10/LC_Bar_SVID24_CN0.png]

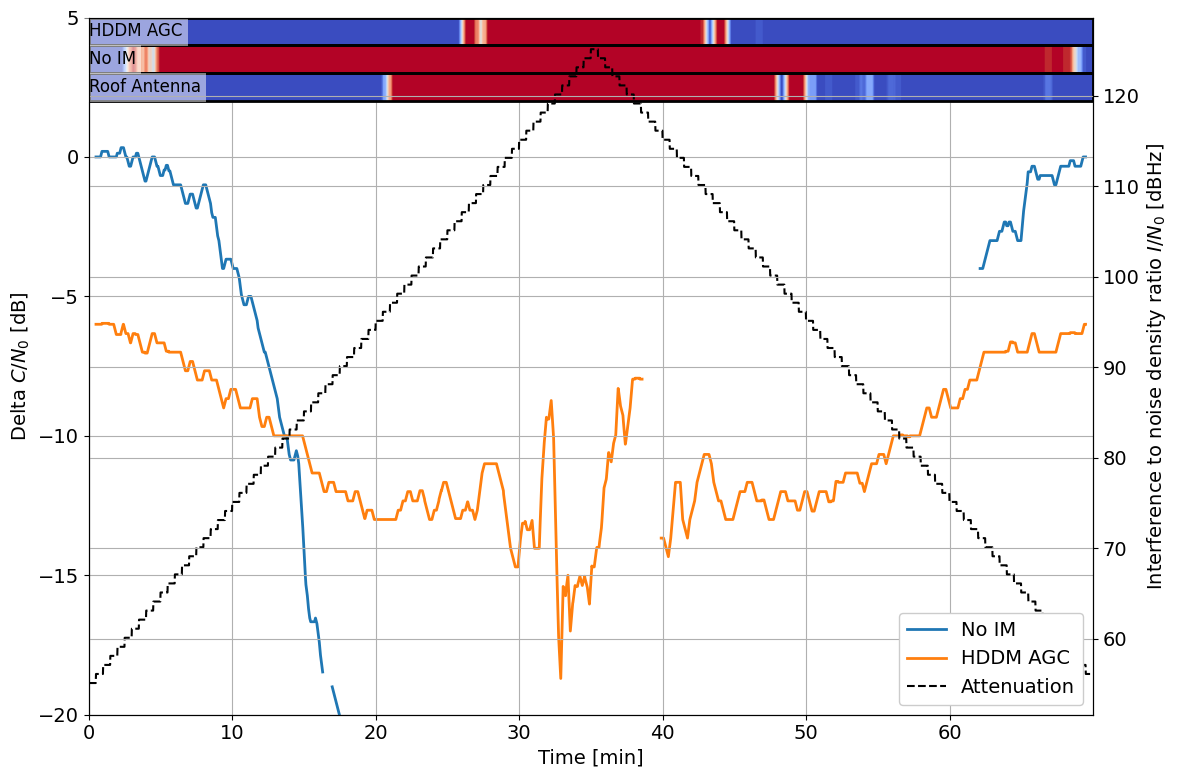

Supplement: Supplementary file 1 [file sensors-22-00679-s001.zip › results/Galileo/E1BC/Chirp_LinearSlow_BW10/LC_Bar_SVID24_DeltaCN0.png]

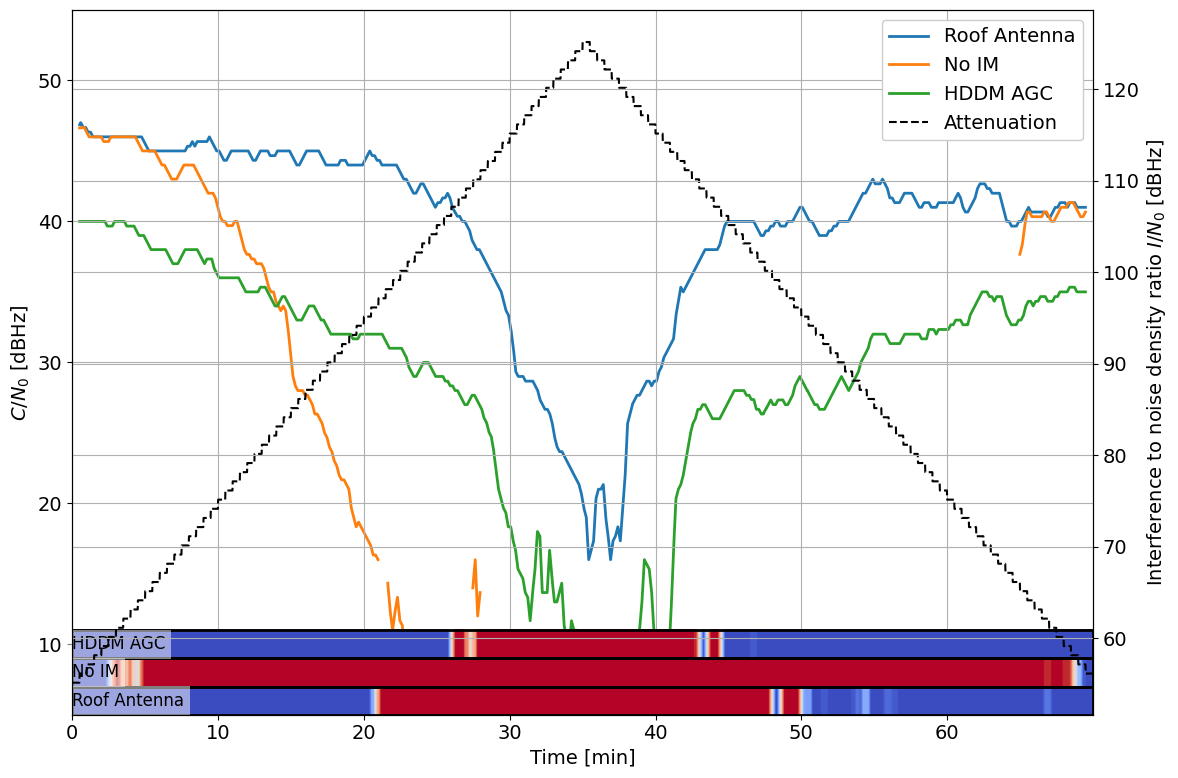

Supplement: Supplementary file 1 [file sensors-22-00679-s001.zip › results/Galileo/E1BC/Chirp_LinearSlow_BW10/LC_Bar_SVID26_CN0.png]

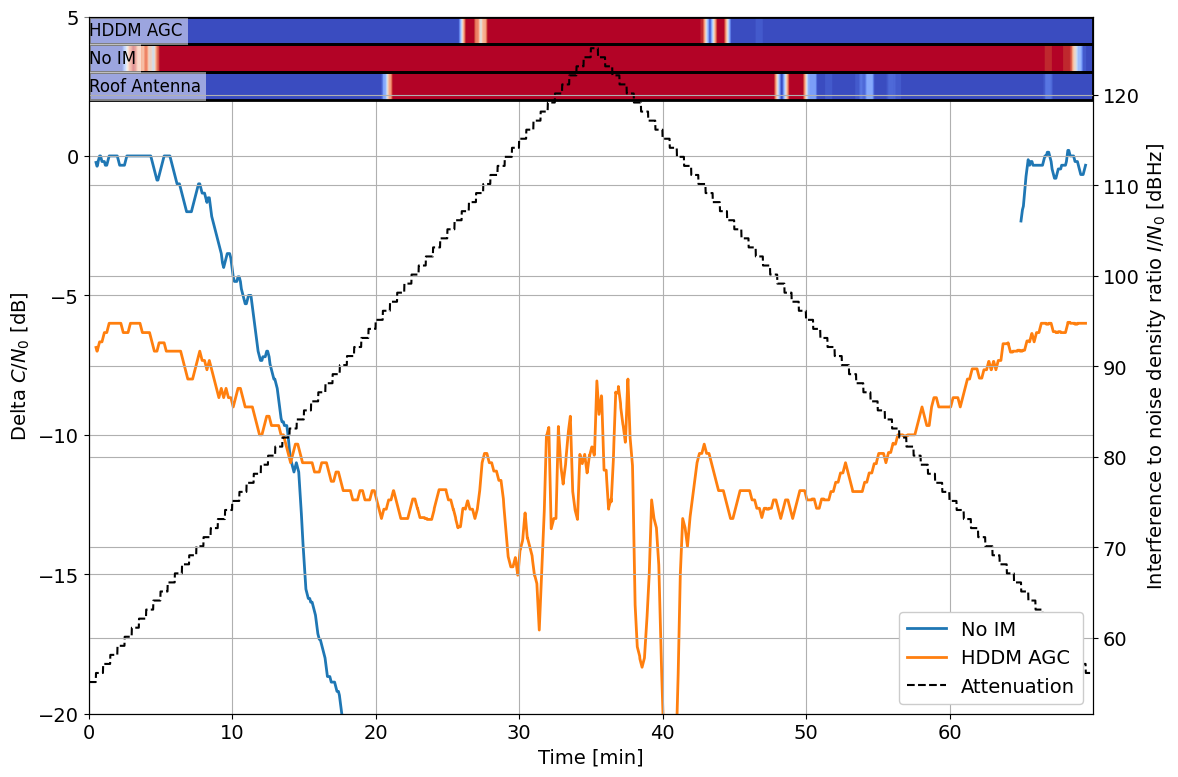

Supplement: Supplementary file 1 [file sensors-22-00679-s001.zip › results/Galileo/E1BC/Chirp_LinearSlow_BW10/LC_Bar_SVID26_DeltaCN0.png]

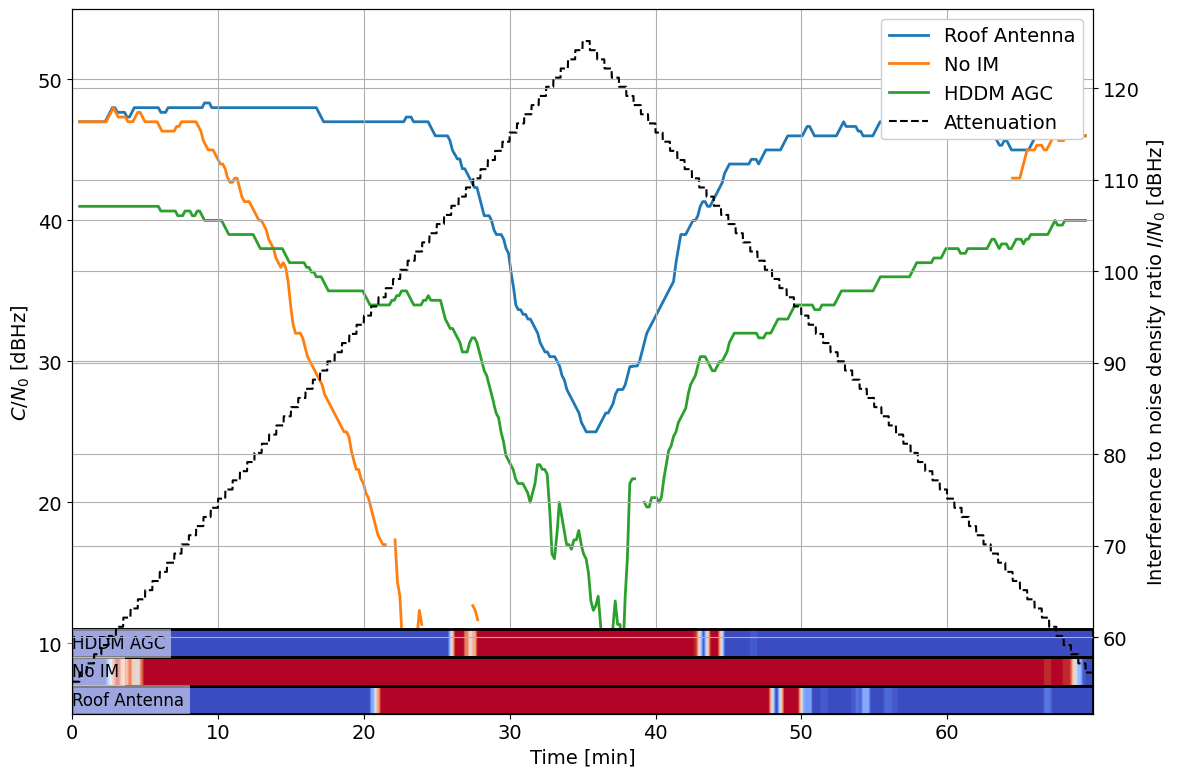

Supplement: Supplementary file 1 [file sensors-22-00679-s001.zip › results/Galileo/E1BC/Chirp_LinearSlow_BW10/LC_Bar_SVID31_CN0.png]

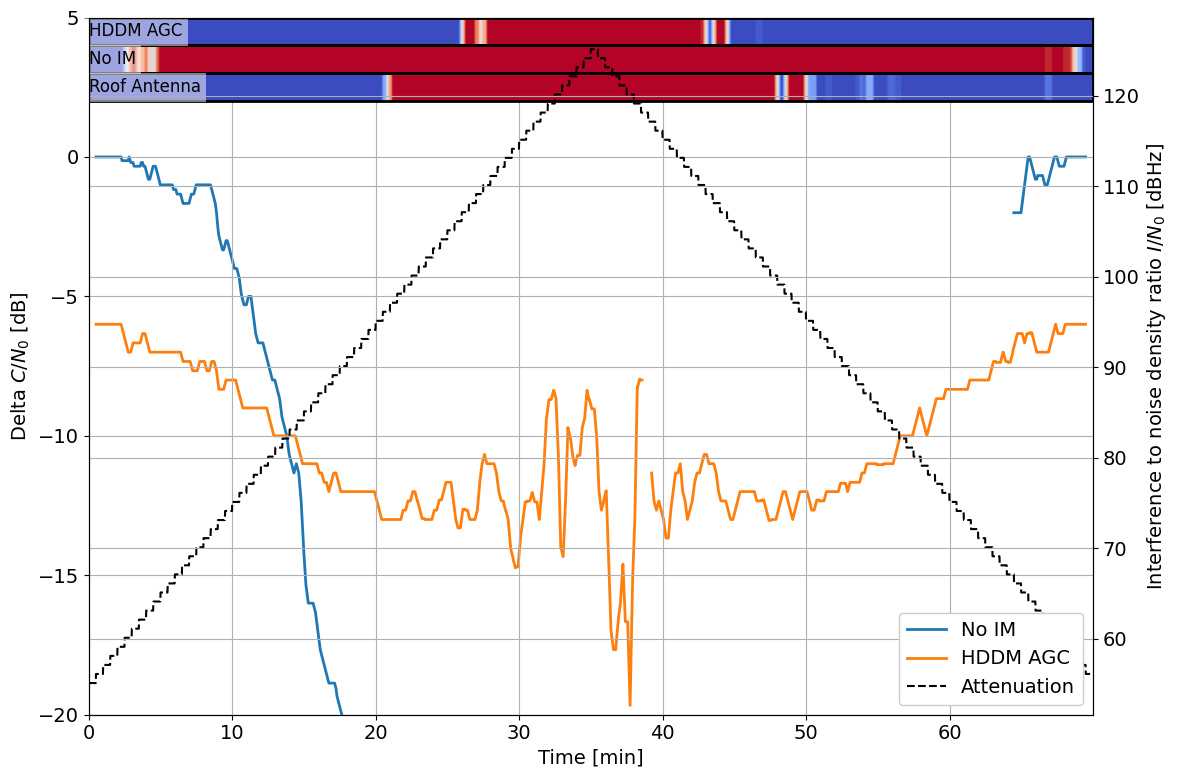

Supplement: Supplementary file 1 [file sensors-22-00679-s001.zip › results/Galileo/E1BC/Chirp_LinearSlow_BW10/LC_Bar_SVID31_DeltaCN0.png]

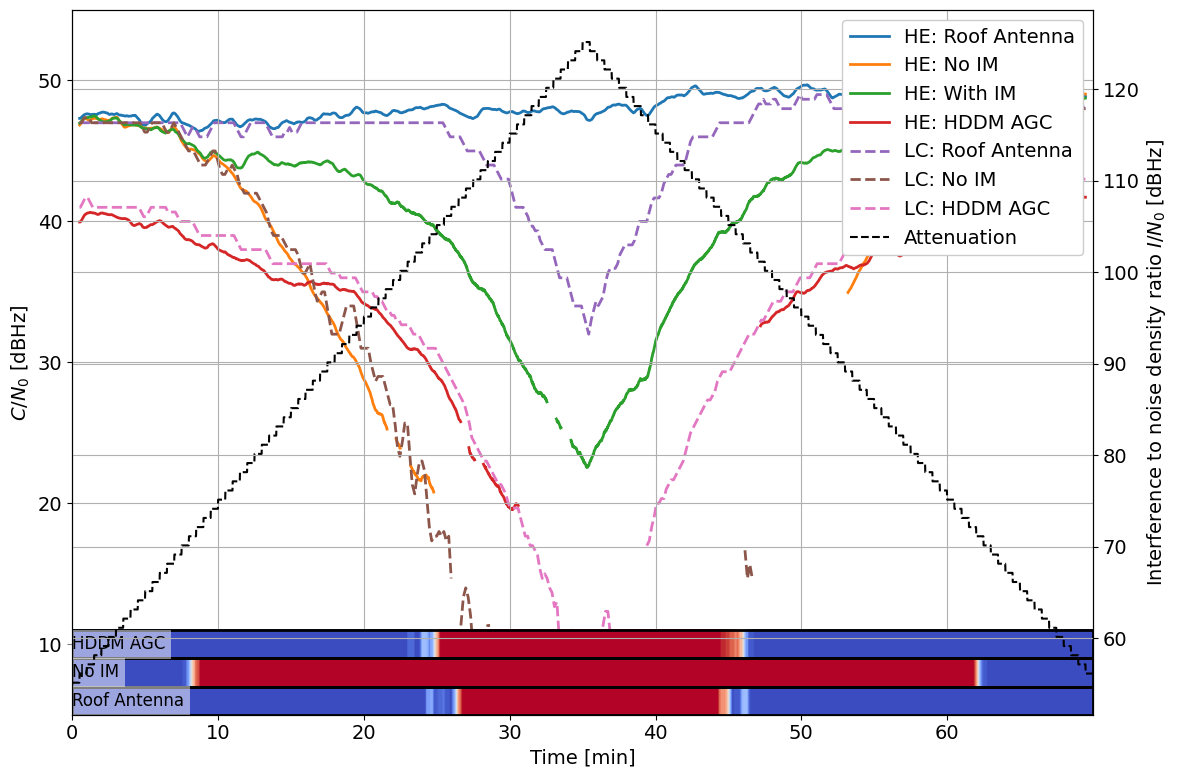

Supplement: Supplementary file 1 [file sensors-22-00679-s001.zip › results/Galileo/E1BC/FreqHopper_BW35MHz_DT100us/HELC_Bar_SVID27_CN0.png]

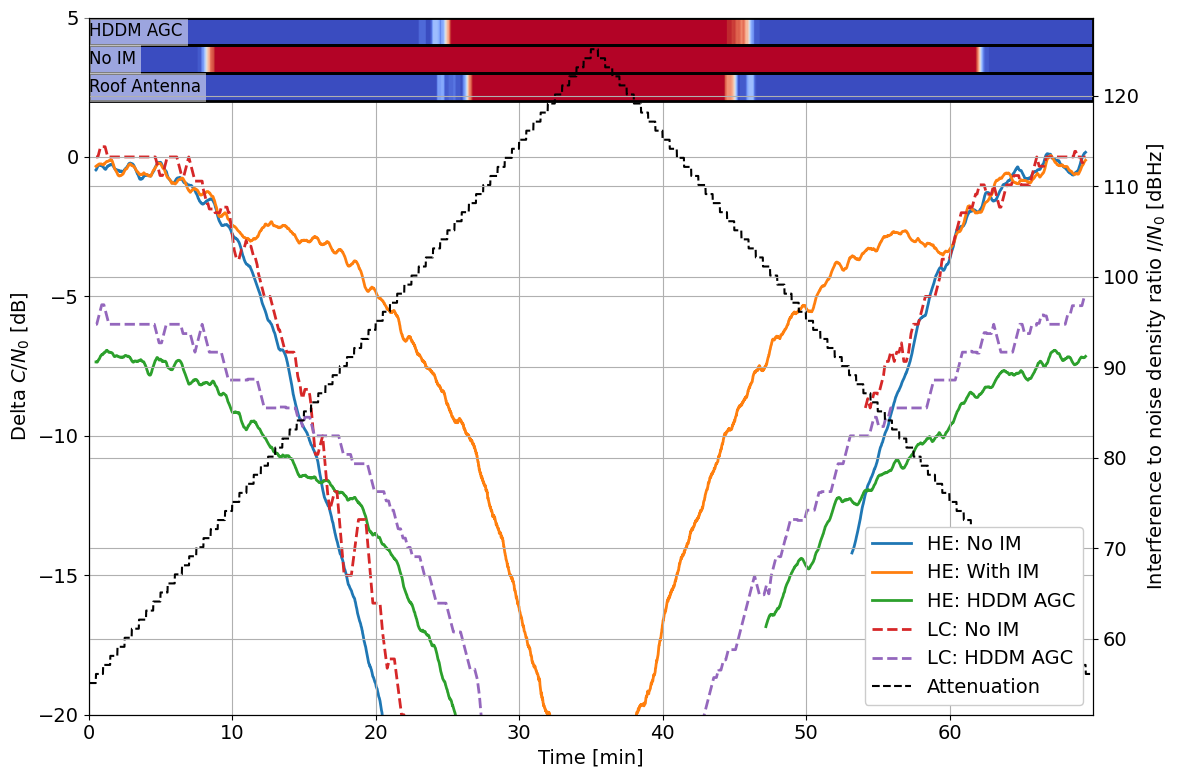

Supplement: Supplementary file 1 [file sensors-22-00679-s001.zip › results/Galileo/E1BC/FreqHopper_BW35MHz_DT100us/HELC_Bar_SVID27_DeltaCN0.png]

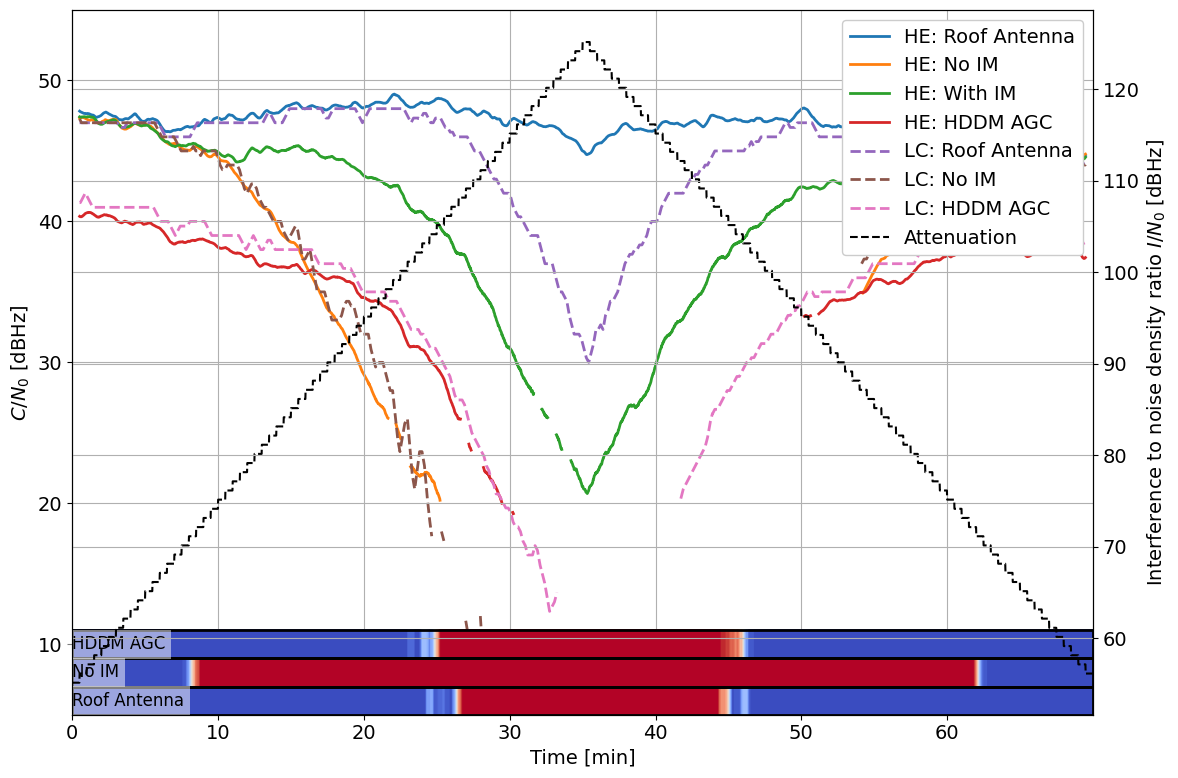

Supplement: Supplementary file 1 [file sensors-22-00679-s001.zip › results/Galileo/E1BC/FreqHopper_BW35MHz_DT100us/HELC_Bar_SVID30_CN0.png]

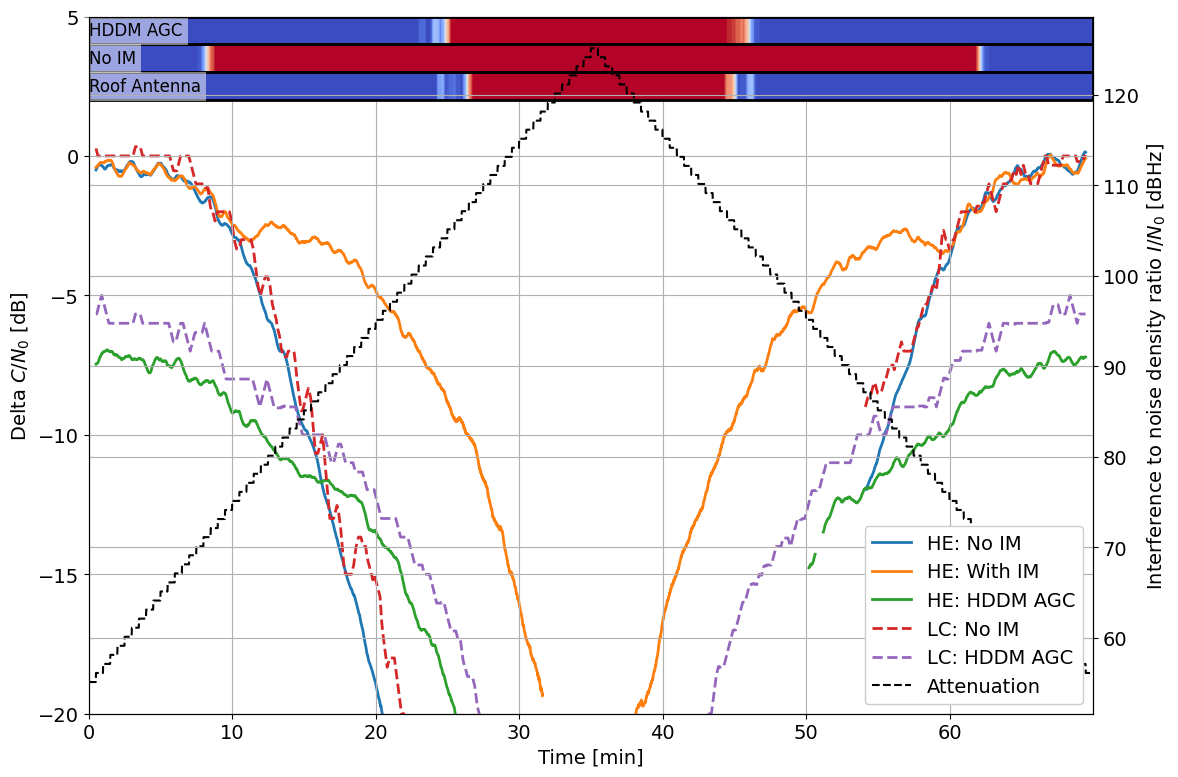

Supplement: Supplementary file 1 [file sensors-22-00679-s001.zip › results/Galileo/E1BC/FreqHopper_BW35MHz_DT100us/HELC_Bar_SVID30_DeltaCN0.png]

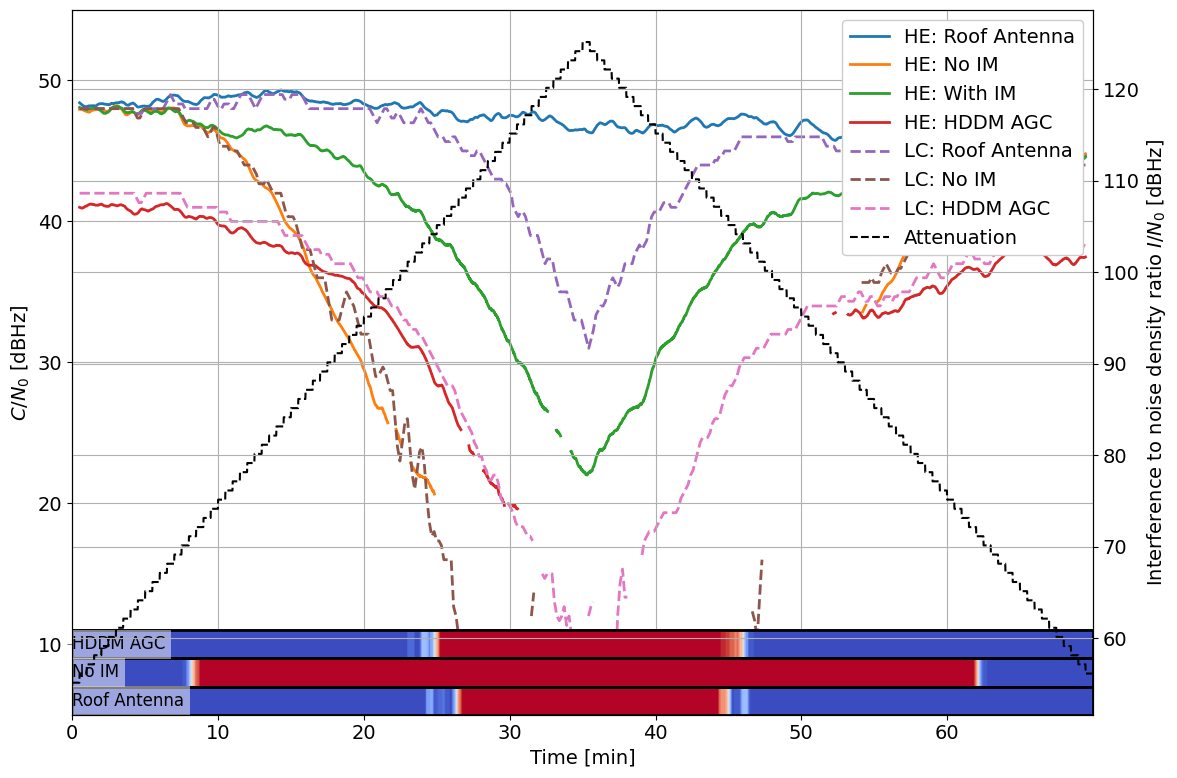

Supplement: Supplementary file 1 [file sensors-22-00679-s001.zip › results/Galileo/E1BC/FreqHopper_BW35MHz_DT100us/HELC_Bar_SVID7_CN0.png]

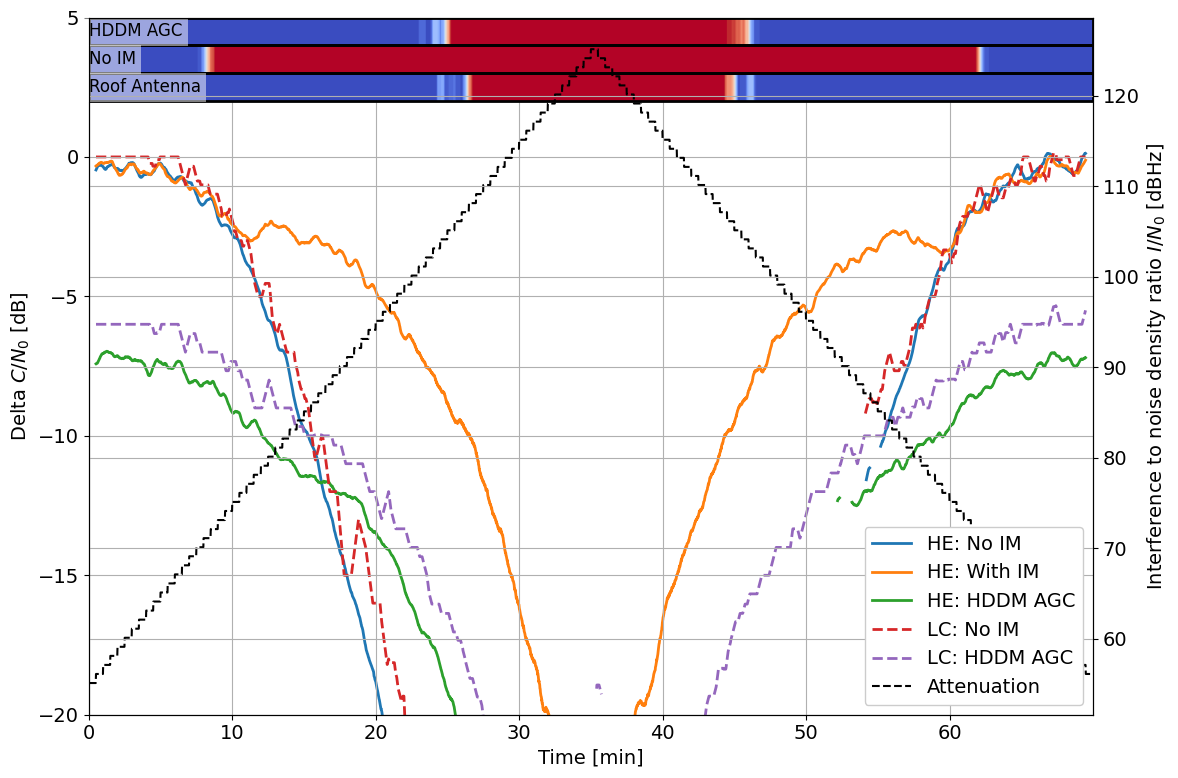

Supplement: Supplementary file 1 [file sensors-22-00679-s001.zip › results/Galileo/E1BC/FreqHopper_BW35MHz_DT100us/HELC_Bar_SVID7_DeltaCN0.png]

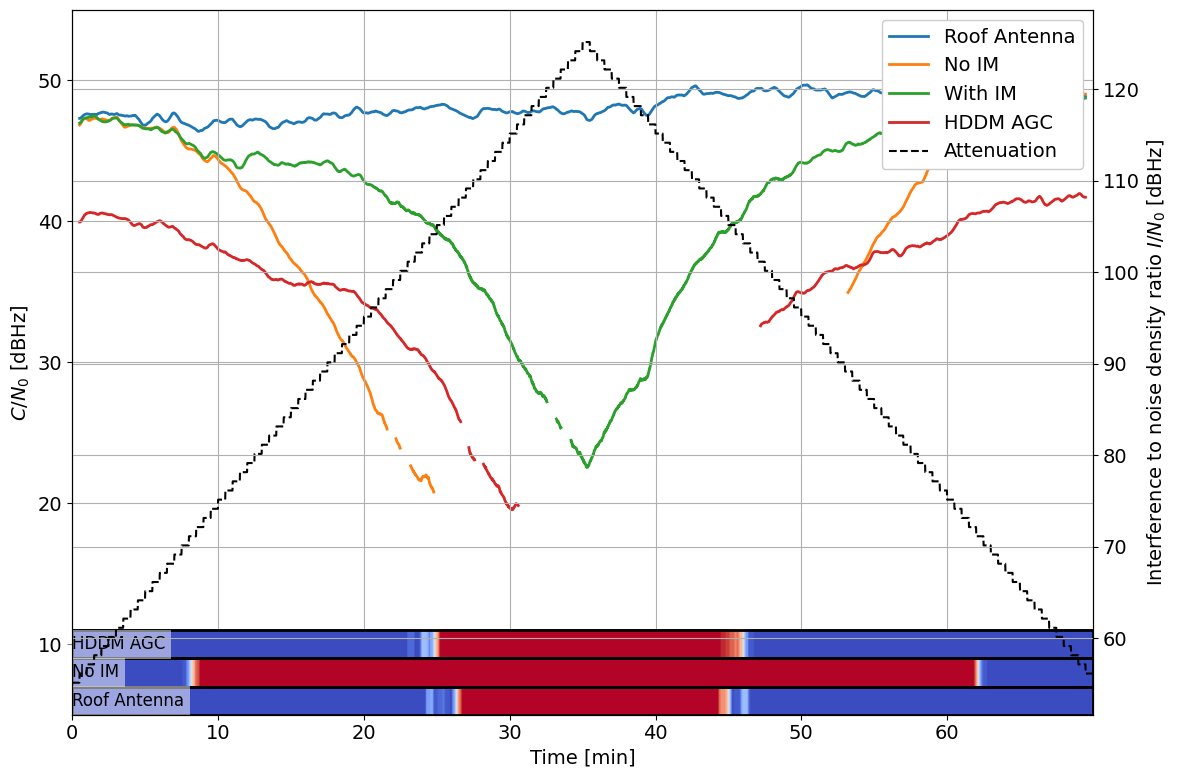

Supplement: Supplementary file 1 [file sensors-22-00679-s001.zip › results/Galileo/E1BC/FreqHopper_BW35MHz_DT100us/HE_Bar_SVID27_CN0.png]

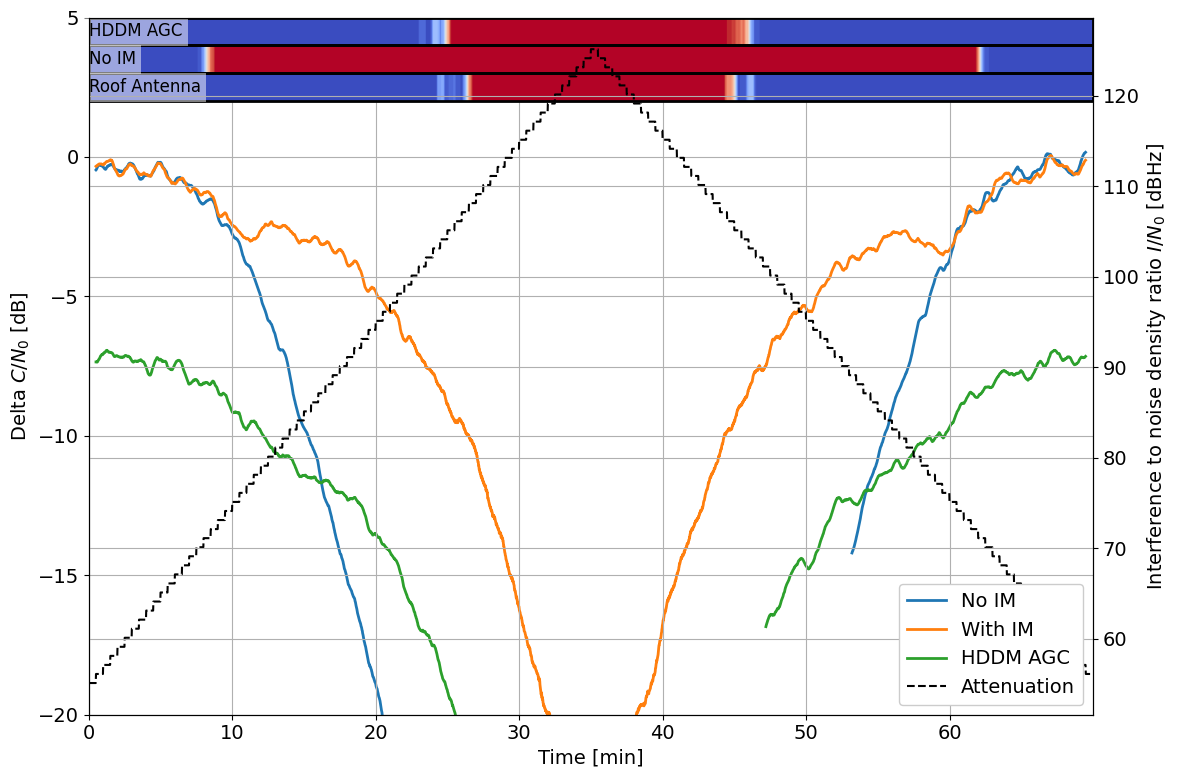

Supplement: Supplementary file 1 [file sensors-22-00679-s001.zip › results/Galileo/E1BC/FreqHopper_BW35MHz_DT100us/HE_Bar_SVID27_DeltaCN0.png]

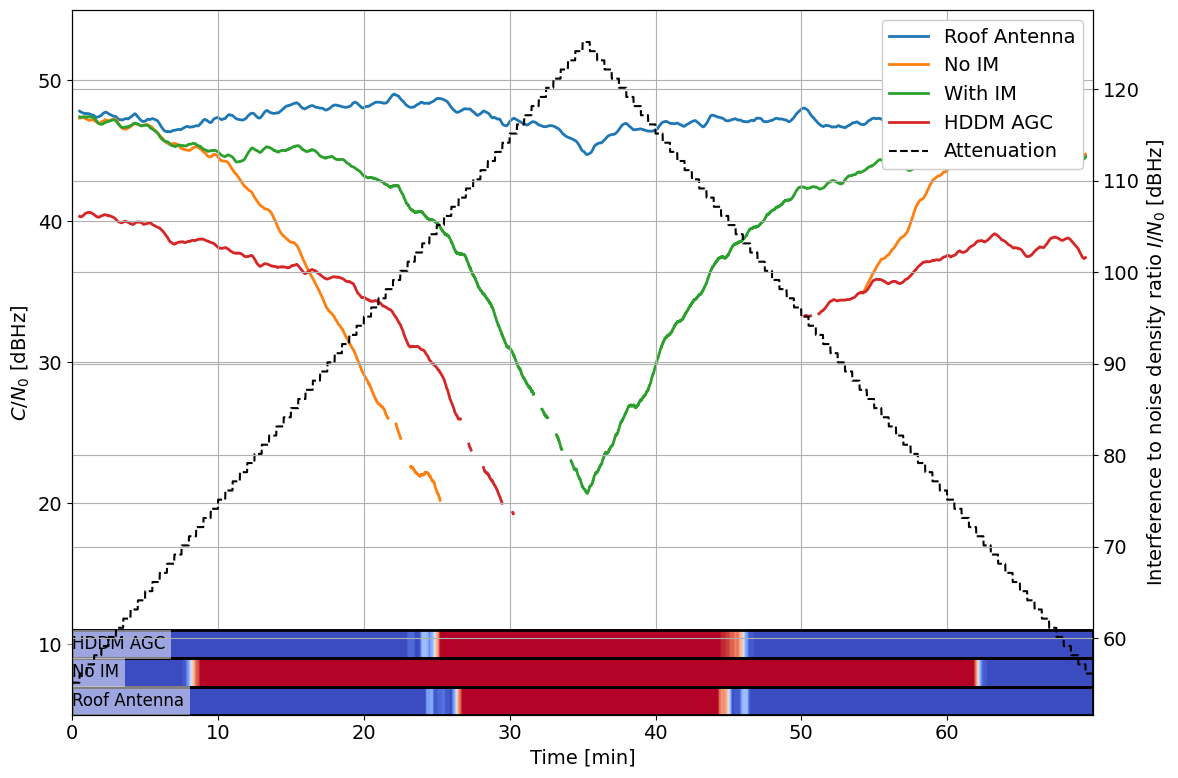

Supplement: Supplementary file 1 [file sensors-22-00679-s001.zip › results/Galileo/E1BC/FreqHopper_BW35MHz_DT100us/HE_Bar_SVID30_CN0.png]

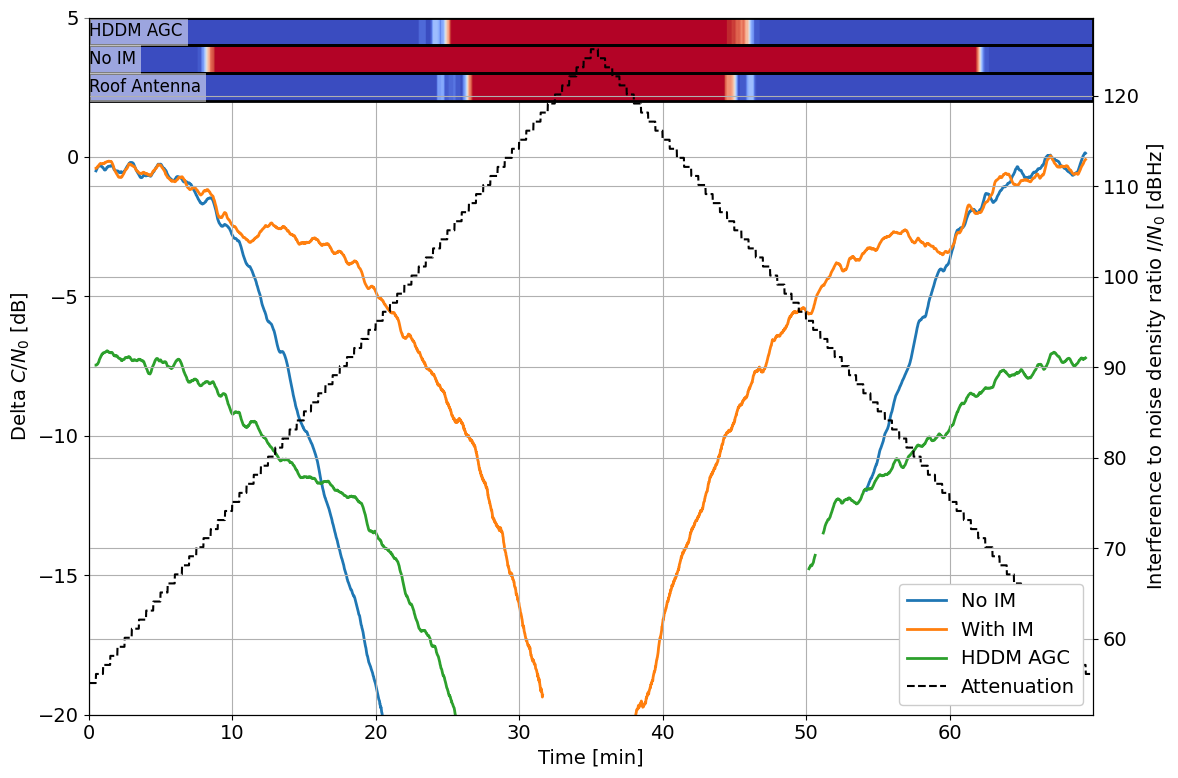

Supplement: Supplementary file 1 [file sensors-22-00679-s001.zip › results/Galileo/E1BC/FreqHopper_BW35MHz_DT100us/HE_Bar_SVID30_DeltaCN0.png]

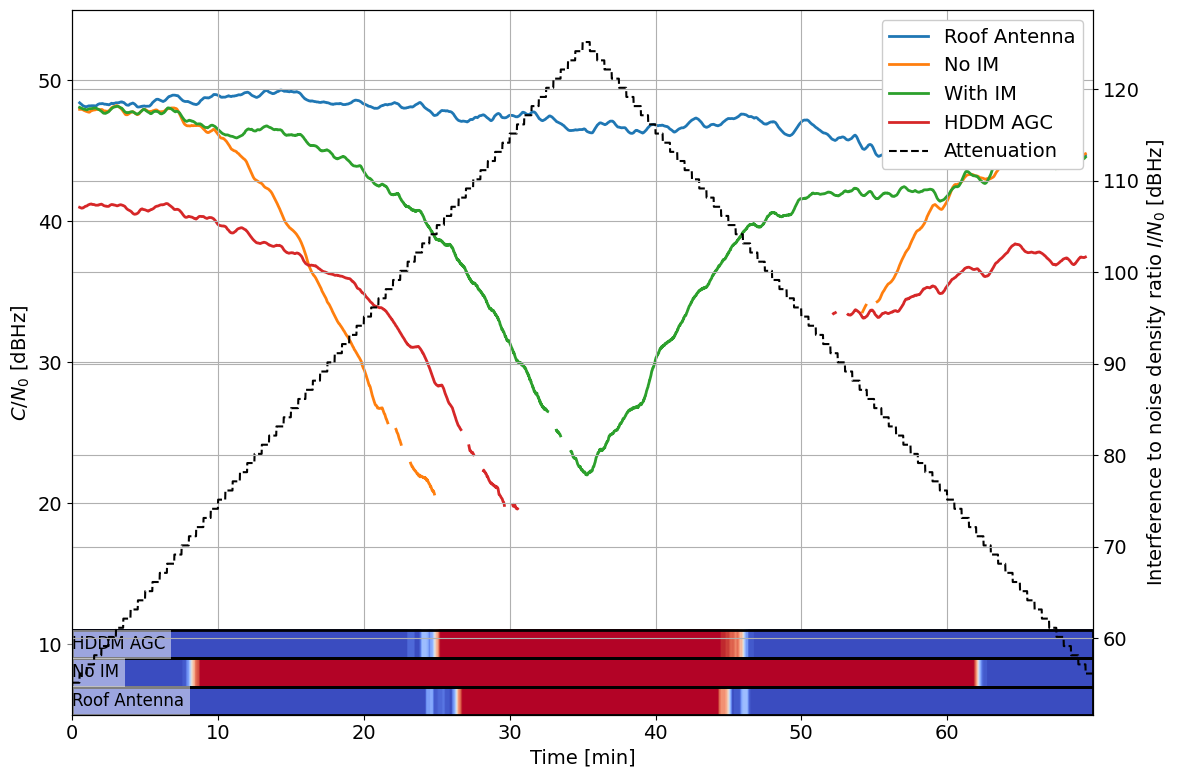

Supplement: Supplementary file 1 [file sensors-22-00679-s001.zip › results/Galileo/E1BC/FreqHopper_BW35MHz_DT100us/HE_Bar_SVID7_CN0.png]

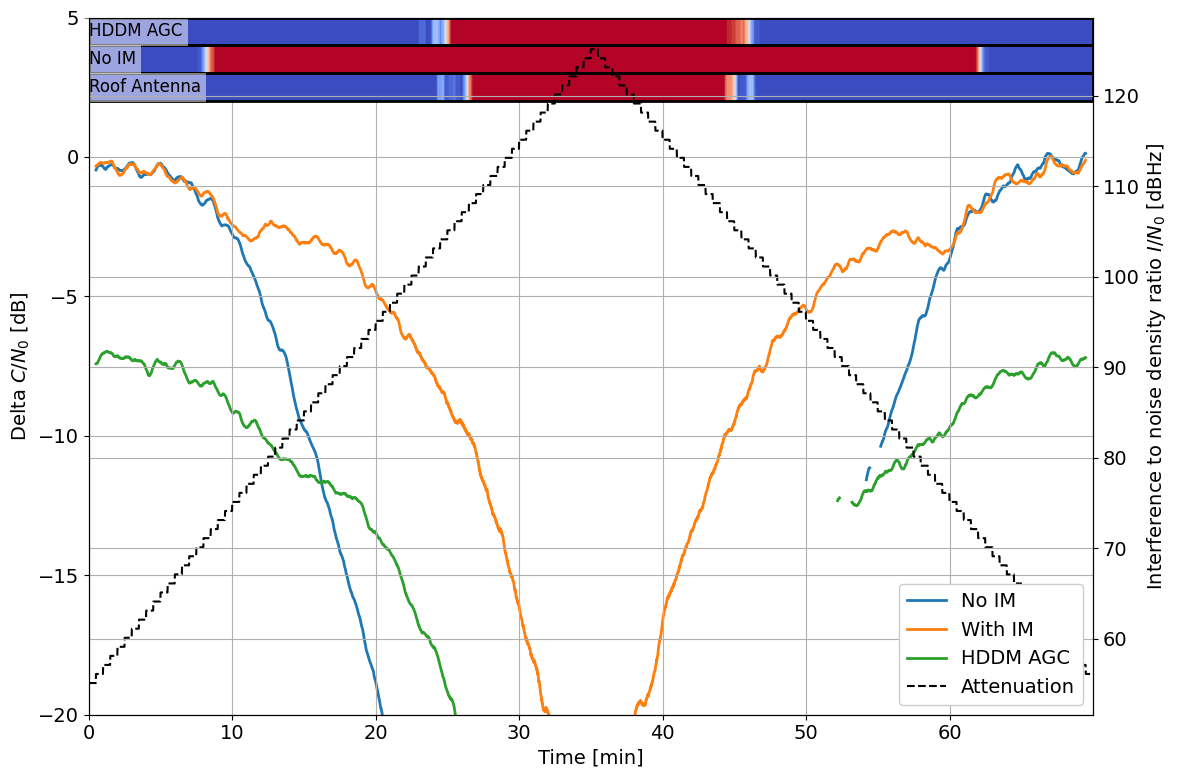

Supplement: Supplementary file 1 [file sensors-22-00679-s001.zip › results/Galileo/E1BC/FreqHopper_BW35MHz_DT100us/HE_Bar_SVID7_DeltaCN0.png]

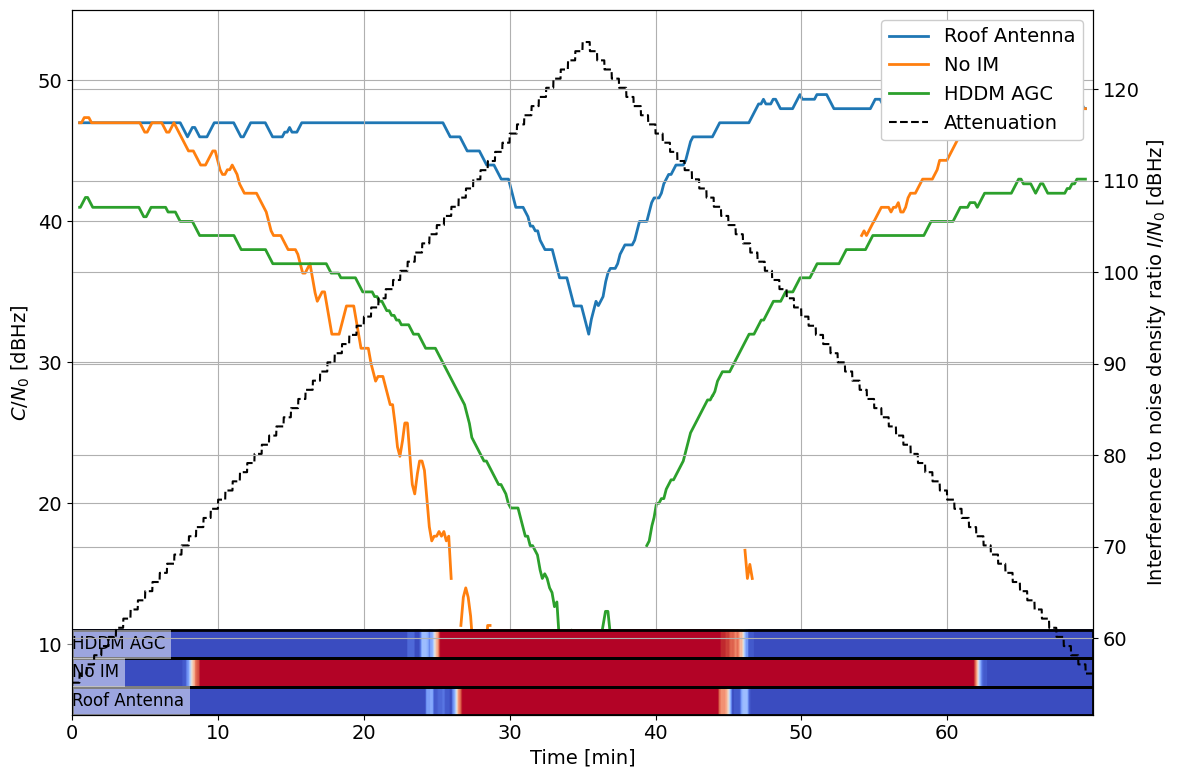

Supplement: Supplementary file 1 [file sensors-22-00679-s001.zip › results/Galileo/E1BC/FreqHopper_BW35MHz_DT100us/LC_Bar_SVID27_CN0.png]

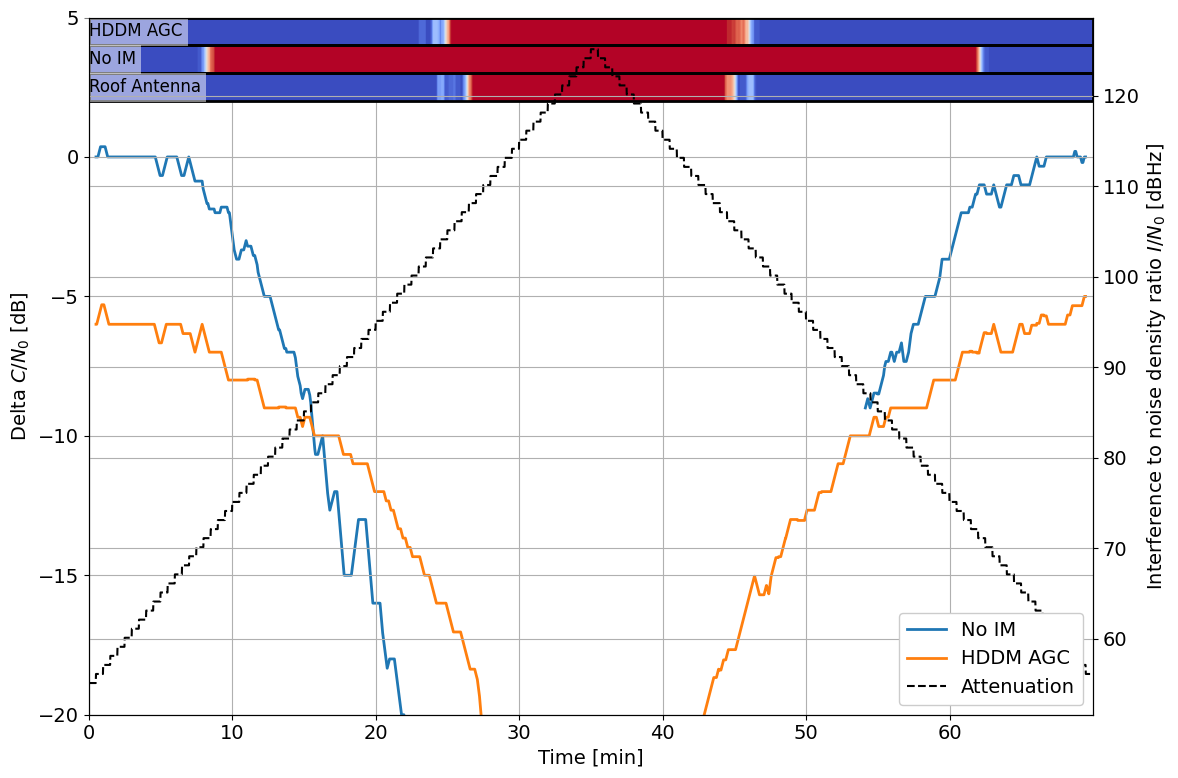

Supplement: Supplementary file 1 [file sensors-22-00679-s001.zip › results/Galileo/E1BC/FreqHopper_BW35MHz_DT100us/LC_Bar_SVID27_DeltaCN0.png]

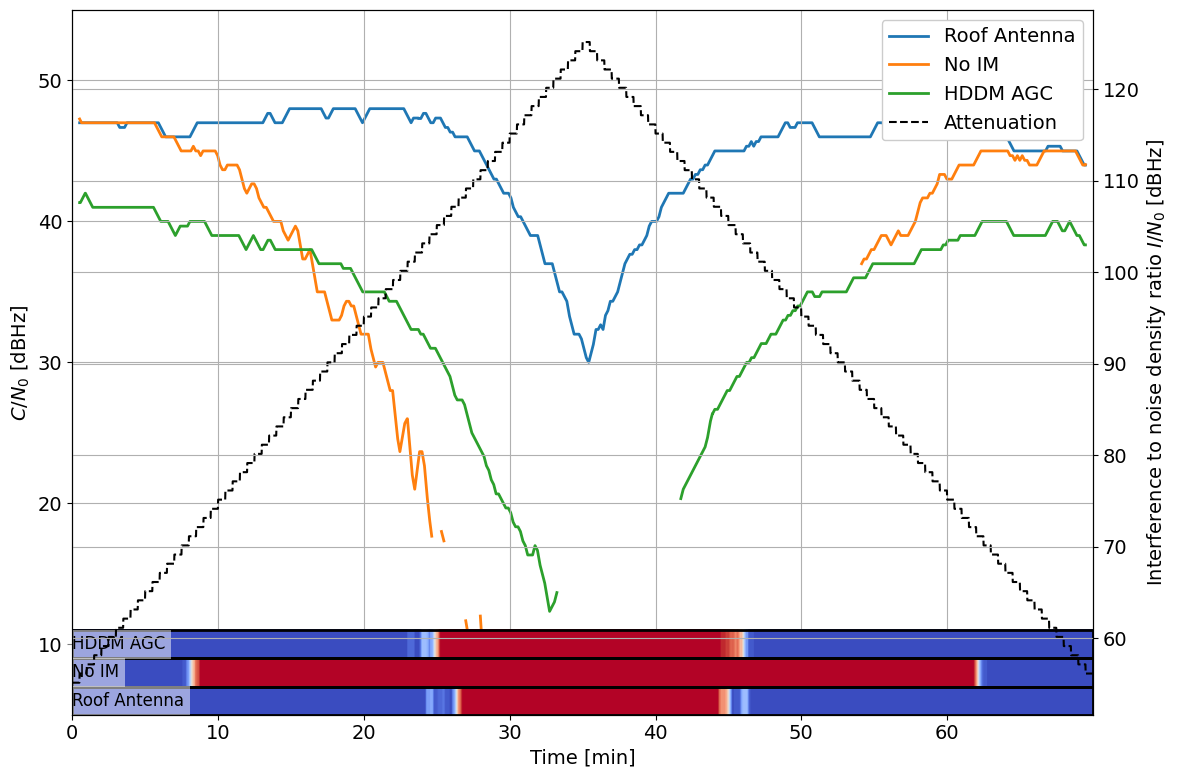

Supplement: Supplementary file 1 [file sensors-22-00679-s001.zip › results/Galileo/E1BC/FreqHopper_BW35MHz_DT100us/LC_Bar_SVID30_CN0.png]

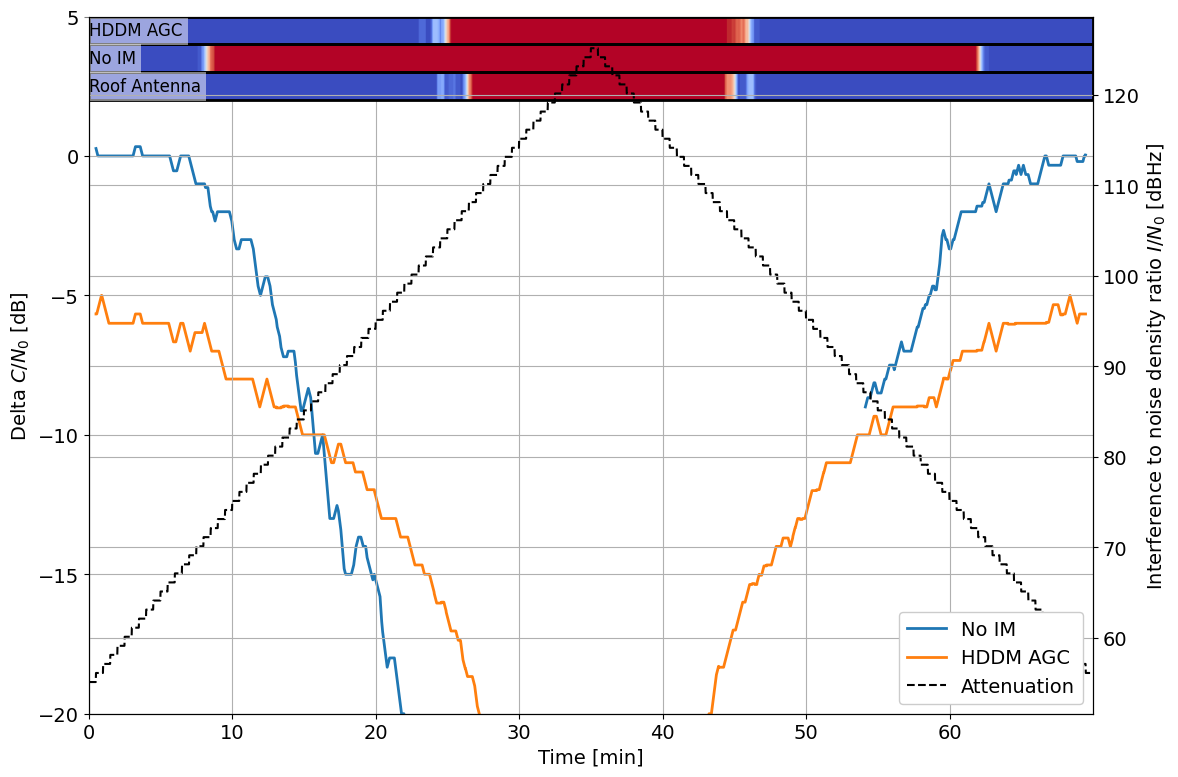

Supplement: Supplementary file 1 [file sensors-22-00679-s001.zip › results/Galileo/E1BC/FreqHopper_BW35MHz_DT100us/LC_Bar_SVID30_DeltaCN0.png]
